# On the monodromy of the deformed cubic oscillator

**DOI:** 10.1007/s00208-021-02337-w

**Published:** 2022-01-04

**Authors:** Tom Bridgeland, Davide Masoero

**Affiliations:** 1 School of Mathematics and Statistics, University of Sheffield, Hicks Building, Hounsfield Road, Sheffield, UK; 2 Department of Mathematics, Faculty of Sciences, Campo Grande, Edifício C6, Lisboa, Portugal

## Abstract

We study a second-order linear differential equation known as the deformed cubic oscillator, whose isomonodromic deformations are controlled by the first Painlevé equation. We use the generalised monodromy map for this equation to give solutions to the Riemann-Hilbert problems of (Bridgeland in Invent Math 216(1):69–124, 2019) arising from the Donaldson-Thomas theory of the A$_2$ quiver. These are the first known solutions to such problems beyond the uncoupled case. The appendix by Davide Masoero contains a WKB analysis of the asymptotics of the monodromy map.

## Introduction

In this paper we study the generalised monodromy map for a second-order linear differential equation known as the deformed cubic oscillator. Our motivation derives from a class of Riemann-Hilbert problems arising naturally in Donaldson-Thomas theory [4], but we hope that our results will be of independent interest. We also suspect that they can be substantially generalized.

### Deformed cubic oscillator

Consider the second-order linear differential equation

1 $$\begin{aligned} y''(x)=Q(x,\hbar ) \cdot y(x), \qquad Q(x,\hbar )=\hbar ^{-2}\cdot {Q_0(x)}+\hbar ^{-1}\cdot Q_1(x) + Q_2(x), \end{aligned}$$

where primes denote differentiation with respect to the complex variable $x\in \mathbb {C}$, and the terms in the potential $Q(x,\hbar )$ are

2 $$\begin{aligned} Q_0(x)= & {} x^3+ax+b, \qquad Q_1(x)=\frac{p}{x-q}+r, \nonumber \\ Q_2(x)= & {} \frac{3}{4(x-q)^2}+\frac{r}{2p(x-q)}+\frac{r^2}{4p^2}. \end{aligned}$$

We view the Eq. (1) as being specified by a point of the complex manifold

3 $$\begin{aligned} M=\bigg \{(a,b,q,p,r)\in \mathbb {C}^5: p^2=q^3+aq+b\text { and } 4a^3+27b^2 \ne 0, \ p\ne 0\bigg \},\nonumber \\ \end{aligned}$$

together with a nonzero complex number $\hbar \in \mathbb {C}^*$ which for now we will consider to be fixed. We also introduce the complex manifold

4 $$\begin{aligned} S=\big \{(a,b)\in \mathbb {C}^2: 4a^3+27b^2\ne 0\big \}, \end{aligned}$$

and the obvious projection map $\pi :M\rightarrow S$.

#### Remark 1.1

The author’s interest in this topic stems from the study of a class of Riemann-Hilbert problems arising in Donaldson-Thomas theory [4, 5]. These problems are specified by a stability condition on a CY$_3$ triangulated category, and involve maps from the complex plane to an algebraic torus with prescribed discontinuities along a collection of rays. In this context the space *S* arises as (a discrete quotient of) the space of stability conditions on the CY$_3$ triangulated category associated to the A$_2$ quiver [6]. As we explain below, the monodromy map for the Eq. (1) gives solutions to the corresponding Riemann-Hilbert problems. These are the first examples of such Riemann-Hilbert problems (beyond the uncoupled case) for which a complete solution is known.

The expression $Q_2(x)$ appearing in (2) is chosen to ensure that the point $x=q$ is an apparent singularity of the Eq. (1): analytically continuing any solution around this point changes its sign. Thus the generalised monodromy of the equation consists only of the Stokes data at the irregular singularity $x=\infty$. As we recall below, this defines a point of the quotient space

5 $$\begin{aligned} V=\Big \{\psi :\mathbb {Z}/5\mathbb {Z}\rightarrow \mathbb {P}^1: \psi (i+1)\ne \psi (i)\text { for all }i\in \mathbb {Z}/5\mathbb {Z}\Big \} \Big / {\text {PGL}}_2, \end{aligned}$$

which is easily seen to be a two-dimensional complex manifold. We thus obtain a holomorphic monodromy map

6 $$\begin{aligned} F(\hbar ):M\rightarrow V. \end{aligned}$$

More precisely, this map depends on a labelling of the Stokes sectors for the equation (1), which in concrete terms amounts to a choice of fifth root of $\hbar ^2$.

#### Remark 1.2

Note that the two points of the space $M$

$$\begin{aligned} (a,b,q,p,r), \qquad \left( a,b+r\hbar +\frac{r^2\hbar ^2}{4p^2},q,p+\frac{r\hbar }{2p},0\right) , \end{aligned}$$

determine the same Eq. (1). Thus for many purposes we can reduce to the situation when $r=0$. In that case (1) coincides, up to trivial changes of variables, with an equation which has been studied in connection with the first Painlevé equation for many years (see [22, Chapter 4] and [28] for references). Nonetheless, it will be important in what follows to consider the full form (2) of the potential, so that the fibres of the map $\pi :M\rightarrow S$ are half-dimensional, and have the same dimension as the monodromy manifold *V*.

Each point $s=(a,b)\in S$ determines a meromorphic quadratic differential on $\mathbb {P}^1$

7 $$\begin{aligned} Q_0(x) \, dx^{\otimes 2} = (x^3+ax+b) \, dx^{\otimes 2} \end{aligned}$$

with a single pole of order seven at $x=\infty$. There is a corresponding branched double cover

8 $$\begin{aligned} p:X_s\rightarrow \mathbb {P}^1, \end{aligned}$$

which is the projectivization of the non-singular plane cubic

9 $$\begin{aligned} X_{s}^\circ =\big \{(x,y)\in \mathbb {C}^2: y^2=x^3+ax+b\big \}. \end{aligned}$$

We also introduce the associated homology groups

10 $$\begin{aligned} \Gamma _s=H_1(X_s,\mathbb {Z})\cong \mathbb {Z}^{\oplus 2}, \end{aligned}$$

which we equip with the standard skew-symmetric intersection form $\langle -,-\rangle$.

#### Remark 1.3

Given an integer $g\ge 0$, and a non-empty collection of integers $m=\{m_1,\cdots ,m_d\}$, with each $m_i\ge 2$, there is a complex orbifold ${\text {Quad}}(g,m)$ parameterizing equivalence-classes of pairs $(S,\phi )$, where *S* is a compact Riemann surface of genus *g*, and $\phi$ is a meromorphic quadratic differential on *S*, having simple zeroes, and poles of the given orders $m_i$. It is shown in [7] that to such data (*g*, *m*) there is naturally associated a CY$_3$ triangulated category $\mathcal {D}(g,m)$, and that the space ${\text {Quad}}(g,m)$ arises as a discrete quotient of the space of stability conditions on $\mathcal {D}(g,m)$.^1^ We expect that the story we describe here (which corresponds to the case $g=0$, $m=\{7\}$) extends to this more general situation, although we do not yet understand the full details of this.

Since the dimensions of the spaces $M$ and *V* are four and two respectively, we might expect the derivative of the monodromy map (6) to have a two-dimensional kernel, and indeed in Sect. 2 we show that the map $F(\hbar )$ is invariant under the two flows

11 $$\begin{aligned}&-\frac{1}{\hbar }\frac{\partial }{\partial r}+\bigg (\frac{\partial }{\partial b}+\frac{1}{2p}\frac{\partial }{\partial p} +\frac{r}{2p^2} \frac{\partial }{\partial r}\bigg ),\end{aligned}$$

12 $$\begin{aligned}&-\frac{2p}{\hbar } \frac{\partial }{\partial q}-\frac{3q^2+a}{\hbar }\frac{\partial }{\partial p}+\bigg (\frac{\partial }{\partial a}-q\frac{\partial }{\partial b}-\frac{r}{p}\frac{\partial }{\partial q}- \frac{r(3q^2+a)}{2p^2}\frac{\partial }{\partial p}-\frac{r^2}{2p^3} (3q^2+a) \frac{\partial }{\partial r}\bigg ).\nonumber \\ \end{aligned}$$

Since the sub-bundle of the tangent bundle spanned by these flows is everywhere transverse to the fibres of the map $\pi :M\rightarrow S$, it defines an Ehresmann connection on this map, which we will refer to as the isomonodromy connection.

It follows from the existence of the isomonodromy connection that the monodromy map $F(\hbar )$ restricts to give local isomorphisms

13 $$\begin{aligned} F(\hbar ):M_s\rightarrow V, \end{aligned}$$

between the fibres $M_s=\pi ^{-1}(s)$ of the projection $\pi :M\rightarrow S$, and the monodromy manifold *V*. What is interesting for us is that, as we will explain below, both sides of the map (13) can be more-or-less identified with the algebraic torus

14 $$\begin{aligned} \mathbb {T}_s=H^1(X_s,\mathbb {C}^*)\cong {\text {Hom}}_\mathbb {Z}(\Gamma _s,\mathbb {C}^*)\cong (\mathbb {C}^*)^2. \end{aligned}$$

Using these identifications allows us to do two things:

(i) We can view the isomonodromy connection as an Ehresmann connection on the bundle over *S* whose fibres are the algebraic tori $\mathbb {T}_s$. We give a Hamiltonian form for this connection in Theorem 1.4, and show that it gives an example of a Joyce structure in the sense of [5]. This structure then induces a flat, torsion-free connection on the tangent bundle of *S*, which is described by Theorem 1.5.
(ii) For each point $s\in S$, we can view the monodromy map (13) as giving a partially-defined automorphism of the algebraic torus $\mathbb {T}_s$, depending in a piecewise holomorphic way on the parameter $\hbar \in \mathbb {C}^*$. This allows us in to solve a family of Riemann-Hilbert problems of the type discussed in [4, 5]. A precise summary of this claim appears as Theorem 1.6 below.

In the next two subsections we will explain these two points in more detail.

### Isomonodromy flows

The homology groups (10) form a local system of lattices over *S*, which induces the Gauss-Manin connection on the vector bundle on *S* whose fibres are the spaces $H_1(X_s,\mathbb {C})$. In concrete terms, we can construct a basis of homology classes by taking inverse images under the double cover (8) of paths in $\mathbb {C}$ connecting the zeroes of $Q_0(x)$. The Gauss-Manin connection is then obtained by keeping these paths locally constant as $Q_0(x)$ varies.

Let us choose a basis $(\gamma _1,\gamma _2)\subset \Gamma _s$ at some point $s\in S$, and extend it to nearby fibres using the Gauss-Manin connection. A particular case of a general result of [7] shows that the expressions

15 $$\begin{aligned} z_i=\int _{\gamma _i} \sqrt{Q_0(x)}\, dx=\int _{\gamma _i} y \, dx, \end{aligned}$$

define a local system of co-ordinates $(z_1,z_2)$ on the manifold *S*.

Consider the bundle $\pi :\mathbb {T}\rightarrow S$ whose fibres are the tori (14). There are obvious local co-ordinates $(\theta _1,\theta _2)$ on the fibres $\mathbb {T}_s$ obtained by writing

$$\begin{aligned} \xi (\gamma _i)=\xi _i=\exp ( \theta _i)\in \mathbb {C}^* \qquad \big (\xi :\Gamma _s\rightarrow \mathbb {C}^*\big )\in \mathbb {T}_s, \end{aligned}$$

and we therefore obtain local co-ordinates $(z_1,z_2,\theta _2,\theta _2)$ on the total space $\mathbb {T}$.

In Sect. 3 we introduce a holomorphic map $\Theta :M\rightarrow \mathbb {T}$, commuting with the two projections to *S*, and given in local co-ordinates (up to multiples of ${\pi } i$) by

16 $$\begin{aligned} \theta _i= -\int _{\gamma _i}\frac{Q_1(x)\, dx}{2\sqrt{Q_0(x)}}=-\int _{\gamma _i} \bigg (\frac{p}{x-q} + r\bigg ) \frac{dx}{2y}. \end{aligned}$$

This expression is familiar in WKB analysis as the constant term in the expansion of the Voros symbols (see Sect. 7.4 below).

In Sect. 3 we give a more geometric description of the map $\Theta$. For each point $s\in S$ we show that there is a natural embedding of the fibre $M_s=\pi ^{-1}(s)$ into the space of pairs $(L,\nabla )$ consisting of a holomorphic line bundle *L* on the elliptic curve $X_s$, equipped with a holomorphic connection $\nabla$. The map $\Theta$ then sends such a pair $(L,\nabla )$ to its holonomy, viewed as an element of $\mathbb {T}_s$.

We shall refer to the map $\Theta$ as the abelian holonomy map. It follows from the above description that it is an open embedding. We can use it to push forward the isomonodromy flows (11)–(12). This gives an Ehresmann connection on a dense open subset of the bundle $\pi :\mathbb {T}\rightarrow S$. The following result shows that this connection has precisely the form considered in [5].

#### Theorem 1.4

When written in the co-ordinates $(z_1,z_2,\theta _1,\theta _2)$, the push-forward of the isomonodromy flows (11)–(12) along the map $\Theta :M\rightarrow \mathbb {T}$ take the Hamiltonian form

17 $$\begin{aligned} \frac{\partial }{\partial z_i} + \frac{1}{\hbar } \cdot \frac{\partial }{\partial \theta _i} +\frac{\partial ^2 J}{\partial \theta _i\partial \theta _1}\cdot \frac{\partial }{\partial \theta _2}-\frac{\partial ^2 J}{\partial \theta _i\partial \theta _2}\cdot \frac{\partial }{\partial \theta _1}, \end{aligned}$$

where $J:\mathbb {T}\rightarrow \mathbb {C}$ is a meromorphic function with no poles on the locus $\theta _1=\theta _2=0$. When pulled-back to *M* using the abelian holonomy map it is given by the expression

$$\begin{aligned} \frac{1}{2{\pi } i}\cdot (J\circ \Theta )= -\frac{ 2ap^2+3p(3b-2aq)r+(6aq^2-9bq+4a^2)r^2 - 2apr^3}{4(4a^3+27b^2)p}. \end{aligned}$$

The pencil of flat non-linear connections (17) defines a geometric structure on the space *S* which is studied in detail in [5] and called there a Joyce structure.^2^ The author expects such structures to exist on spaces of stability conditions of CY$_3$ triangulated categories in much greater generality, and Theorem 1.4 provides an interesting first example. We call the function *J* the Joyce function; some of its basic properties are discussed in Sect. 4.4 below.

The Joyce function $J=J(z_1,z_2,\theta _1,\theta _2)$ is easily seen to be odd in the variables $\theta _1,\theta _2$, and it follows that the flows (17) preserve the section of the bundle $\pi :\mathbb {T}\rightarrow S$ defined by setting $\theta _1=\theta _2=0$. They therefore induce a linear connection on the normal bundle to this section, which can in turn be identified with the tangent bundle to *S* via the map

$$\begin{aligned} \frac{\partial }{\partial \theta _i}\mapsto \frac{\partial }{\partial z_i}. \end{aligned}$$

In this way we obtain a linear, flat, torsion-free connection on the tangent bundle of *S*, given explicitly by the formula

$$\begin{aligned} \nabla _{\frac{\partial }{\partial z_i}}\Big (\frac{\partial }{\partial z_j}\Big )= \frac{\partial ^3 J}{\partial \theta _i \, \partial \theta _j \, \partial \theta _2}\Big |_{\theta =0} \cdot \frac{\partial }{\partial z_1}-\frac{\partial ^3 J}{\partial \theta _i \, \partial \theta _j \, \partial \theta _1}\Big |_{\theta =0} \cdot \frac{\partial }{\partial z_2}. \end{aligned}$$

We call it the linear Joyce connection. In Sect. 4 we prove

#### Theorem 1.5

The functions (*a*, *b*) are flat co-ordinates for the linear Joyce connection.

We will comment on the significance of this result after the statement of Theorem 1.6 below.

### Riemann-Hilbert problem

Consider now the right-hand side of the monodromy map (13). It is well known that the manifold *V* has a system of birational co-ordinate systems

18 $$\begin{aligned} X_T:V\dashrightarrow (\mathbb {C}^*)^2, \end{aligned}$$

indexed by the triangulations *T* of a regular pentagon. These co-ordinate systems are usually called Fock-Goncharov co-ordinates, since they appear in a much more general context in [14]. We recall their definition in Sect. 7. The co-ordinates corresponding to different triangulations are related by post-composition with explicit birational automorphisms of $(\mathbb {C}^*)^2$.

Let us fix a point $(a,b,q,p,r)\in M$. For generic $\hbar \in \mathbb {C}^*$, the horizontal trajectory structure of the quadratic differential

19 $$\begin{aligned} \hbar ^{-2}\cdot Q_0(x) \, dx^{\otimes 2}= \hbar ^{-2}\cdot (x^3+ax+b) \, dx^{\otimes 2}\end{aligned}$$

determines a triangulation $T(\hbar )$ of a regular pentagon. This triangulation is well-defined when $\hbar \in \mathbb {C}^*$ lies in the complement of the finitely-many rays on which the quadratic differential (19) has a finite-length horizontal trajectory. Following [17] we refer to it as the WKB triangulation.

When $T=T(\hbar )$ is a WKB triangulation, the algebraic torus appearing on the right-hand side of (18) is naturally identified with the torus $\mathbb {T}_s$ associated to the point $s=(a,b)\in S$. Keeping the point $(a,b,q,p,r)\in M$ fixed, let us now consider the map

20 $$\begin{aligned} X :\mathbb {C}^*\rightarrow \mathbb {T}_s,\qquad X(\hbar )=X_{T(\hbar )}\big (F(\hbar )(a,b,q,p,r)\big ), \end{aligned}$$

which sends a point $\hbar \in \mathbb {C}^*$ to the Fock-Goncharov co-ordinates of the monodromy of the Eq. (1) with respect to the WKB triangulation $T(\hbar )$. Using our chosen basis $(\gamma _1,\gamma _2)$ of $\Gamma _s$ we can identify $\mathbb {T}_s$ with $(\mathbb {C}^*)^2$ and decompose $X(\hbar )$ into its components

$$\begin{aligned} X(\hbar )=(x_1(\hbar ),x_2(\hbar ))\in (\mathbb {C}^*)^2, \qquad x_i(\hbar )=X(\hbar )(\gamma _i)\in \mathbb {C}^*. \end{aligned}$$

The map (20) has three important properties, which we explain in detail in Sect. 7:

(i) As $\hbar \in \mathbb {C}^*$ crosses a ray where the differential (19) has a finite-length horizontal trajectory, the WKB triangulation $T(\hbar )$ changes, and the map $X(\hbar )$ undergoes a discontinuous jump obtained by post-composing with an explicit birational transformation of the torus $\mathbb {T}_s$.
(ii) The WKB approximation can be used to show that as $\hbar \rightarrow 0$ along a ray in $\mathbb {C}^*$
  $$\begin{aligned} x_i(\hbar )\cdot \exp \Big (\frac{z_i}{\hbar }-\theta _i\Big )\rightarrow 1 \end{aligned}$$
  where the $\theta _i$ are given by (16). This statement is proved in the Appendix.
(iii) A homogeneity property of the potential (2) allows us to conclude that as $\hbar \rightarrow \infty$ the functions $x_i(\hbar )$ have a well-defined limit.

These properties are exactly the conditions required for the map $X(\hbar )$ to give a solution to one of the Riemann-Hilbert problems defined in [4]. To state this more precisely, recall first the definition of a finite BPS structure $(\Gamma ,Z,\Omega )$ from [4]. It consists of

- A finite-rank free abelian group $\Gamma \cong \mathbb {Z}^{\oplus n}$, equipped with a skew-symmetric form
  $$\begin{aligned} \langle -,-\rangle :\Gamma \times \Gamma \rightarrow \mathbb {Z}, \end{aligned}$$
- A homomorphism of abelian groups $Z:\Gamma \rightarrow \mathbb {C}$,
- A map of sets $\Omega :\Gamma \rightarrow \mathbb {Q}$ such that $\Omega (\gamma )=0$ for all but finitely-many elements $\gamma \in \Gamma$, and satisfying the symmetry property $\Omega (-\gamma )=\Omega (\gamma )$.

The group $\Gamma$ is called the charge lattice, and the homomorphism *Z* the central charge. The rational numbers $\Omega (\gamma )$ are called the BPS invariants.

As we explain in Sect. 6, each point $s\in S$ determines such a BPS structure $(\Gamma _s,Z_s,\Omega _s)$. The charge lattice is the homology group $\Gamma _s=H_1(X_s,\mathbb {Z})$ equipped with its intersection form $\langle -,-\rangle$. The central charge $Z_s:\Gamma _s\rightarrow \mathbb {C}$ is defined by the formula

$$\begin{aligned} Z_s(\gamma )=\int _{\gamma } \sqrt{Q_0(x)} \, dx\in \mathbb {C}. \end{aligned}$$

Assuming that the point $s\in S$ is generic, in the sense that the image of $Z_s$ is not contained in a line, the BPS invariants $\Omega _s(\gamma )\in \mathbb {Z}$ count the number of finite-length trajectories of the differential (7) whose lifts to $X_s$ define the given class $\gamma \in \Gamma$.

It is explained in [4] how to associate a Riemann-Hilbert problem to a finite BPS structure. This problem involves piecewise holomorphic (or meromorphic) maps into the associated algebraic torus $\mathbb {T}$, and depends on an element $\xi \in \mathbb {T}$ called the constant term. Our final result is

#### Theorem 1.6

Take a point $(a,b,q,p,r)\in M$ and let $(\Gamma _s,Z_s,\Omega _s)$ be the BPS structure determined by the corresponding point $(a,b)\in S$. Then the map (20) gives a meromorphic solution to the Riemann-Hilbert problem for this BPS structure, with constant term $\xi \in \mathbb {T}_s$ defined by (16).

Let us return to the abstract context of Remark 1.1, where the space *S* appears as a discrete quotient of the space of stability conditions on the CY$_3$ triangulated category associated to the A$_2$ quiver. The BPS structures $(\Gamma _s,Z_s,\Omega _s)$ considered above then coincide with those defined by the Donaldson-Thomas theory of these stability conditions. Thus Theorem 1.6 gives solutions to the Riemann-Hilbert problems defined by the A$_2$ quiver. It is worth noting in this context that the space *V* also has a natural representation-theoretic meaning, since it coincides with the cluster Poisson variety.

When viewed from this abstract point of view, the only natural local co-ordinates on the stability space *S* are the central charge co-ordinates $(z_1,z_2)$. The point of Theorem 1.5 is that it gives a way to derive the flat structure on *S* whose co-ordinates are (*a*, *b*) from purely abstract considerations: one first solves the Riemann-Hilbert problem defined by the Donaldson-Thomas invariants to obtain the pencil of non-linear connections of Theorem 1.4, and then differentiates to obtain the linear connection of Theorem 1.5. Unfortunately there is one crucial missing link in this chain of reasoning: we currently have no characterisation or uniqueness result for the solution of Theorem 1.6.

#### Remark 1.7

The statement of Theorem 1.6 takes direct inspiration from the work of Gaiotto, Moore and Neitzke [16, 17]. In particular, the use of the Fock-Goncharov co-ordinates for the WKB triangulation, and the resulting discontinuities in the map (20) are exactly as described in [17, Section 7]. It is important to note however that the picture described here is strictly different to that of [17]. Although Gaiotto, Moore and Neitzke start with the same data of a BPS structure, they consider a somewhat different Riemann-Hilbert problem, which has non-holomorphic dependence on the central charge *Z*. Instead of our monodromy map *F*, they solve their Riemann-Hilbert problem using a $C^{\infty }$ isomorphism between the moduli spaces of irregular Higgs bundles and the wild character variety *V*. In physical terms what we are considering here is the conformal limit [15] of their story.

#### Remark 1.8

The constructions of this paper are closely related to the ODE/IM correspondence. The author is unfortunately not qualified to describe this link in any detail. It is explained in [15] and [16, Appendix E] that the Riemann-Hilbert problems considered here can be solved, at least formally, by an integral equation known in the integrable systems literature as the Thermodynamic Bethe Ansatz (TBA). The fact that these TBA equations also appear in the analysis of Stokes data of ordinary differential equations goes back in some form to work of Sibuya and Voros, but was made more precise in the work of Dorey, Dunning, Tateo and others. We refer the reader to [10] for a review of the ODE/TBA correspondence, and to [25, 26] for more recent papers which deal specifically with the cubic oscillator.

### Plan of the paper

We begin in Sect. 2 by describing the monodromy of the deformed cubic oscillator (1) and deriving the isomonodromy flows (11)–(12). Section 3 discusses the abelian holonomy map (16) and derives an explicit formula in terms of Weierstrass elliptic functions. In Sect. 4 we compute the push-forward of the the isomonodromy connection via the abelian holonomy map, which leads to a formula for the Joyce function, and a proof of Theorem 1.4.

The second half of the paper begins in Sect. 5 with abstract material on BPS structures and their associated Riemann-Hilbert problems. This is mostly taken from [4], although the exposition can be considerably simplified in the special case considered here. Section 6 describes the quadratic differentials that are parameterised by the space *S*, and the BPS structures defined by their finite-length horizontal trajectories. The corresponding Riemann-Hilbert problems are solved in Sect. 7 using the monodromy map for the deformed cubic oscillator (1). A crucial aspect of this is the WKB analysis used to describe the behaviour of the monodromy map as $\hbar \rightarrow 0$, which is explained in detail in an Appendix written by Davide Masoero.

## The deformed cubic oscillator

In this section we discuss the generalised monodromy data of the deformed cubic oscillator equation (1). We explain why this consists entirely of the Stokes data at $x=\infty$ and recall how this is parameterised by collections of subdominant solutions. We then derive the isomonodromy flow in the form (11)–(12). This section contains only very minor extensions of previously known results. Similar material can be found for example in [27, 28].

### Apparent singularity

The first claim is that for any $\hbar \in \mathbb {C}^*$ and $(a,b,q,p,r)\in M$ the Eq. (1) has an apparent singularity at $x=q$. By this we mean that the analytic continuation of any solution around this point has the effect of multiplying it by $\pm 1$ (and in our case the sign is $-1$). This statement follows immediately from the identity

$$\begin{aligned} \bigg (\frac{p}{\hbar }+\frac{r}{2p}\bigg )^2=\frac{q^3 + aq + b}{\hbar ^2}+\frac{r}{\hbar } +\frac{r^2}{4p^2}, \end{aligned}$$

and the following well-known Lemma.

#### Lemma 2.1

Fix a point $q\in \mathbb {C}$ and suppose that *Q*(*x*) is a meromorphic function having a pole at $x=q$. Suppose further that the Laurent expansion of *Q*(*x*) at this point takes the form

$$\begin{aligned} Q(x)=\frac{3}{4(x-q)^2} +\frac{u}{x-q}+v +O(x-q) . \end{aligned}$$

Then the differential equation

21 $$\begin{aligned} y''(x)=Q(x)\cdot y(x) \end{aligned}$$

has an apparent singularity at $x=q$ precisely if the relation $u^2=v$ holds.

#### Proof

This is a standard calculation using the Frobenius method, and we just give a sketch. We look for a solution to (21) of the form

22 $$\begin{aligned} y(x)=\sum _{i=0}^\infty c_i (x-q)^{\uplambda +i},\qquad c_i\in \mathbb {C}, \end{aligned}$$

with $c_0\ne 0$ and $\uplambda \in \mathbb {C}$. This leads to a recurrence relation

23 $$\begin{aligned} (\uplambda +i)(\uplambda +i-1) c_i =\tfrac{3}{4} c_i + u c_{i-1} + v c_{i-2} + \cdots \end{aligned}$$

which is valid for all $i\ge 0$ if we define $c_i=0$ for $i<0$. In particular, taking $i=0,1,2$ we obtain the relations

24 $$\begin{aligned} (\uplambda ^2-\uplambda - \tfrac{3}{4})c_0=0, \qquad (\uplambda ^2+\uplambda - \tfrac{3}{4}) c_1=u c_0, \qquad (\uplambda ^2+3\uplambda + \tfrac{5}{4})c_2=uc_1+vc_0.\nonumber \\ \end{aligned}$$

The first of these gives the indicial equation, whose roots are $\uplambda =\tfrac{3}{2}$ and $\uplambda =-\tfrac{1}{2}$. When $\uplambda =\tfrac{3}{2}$ it is easy to see that the recursion (23) has a unique solution for each choice of $c_0$, and standard theory then shows that (22) defines a double-valued solution to (21) near $x=q$.

When $\uplambda =-\tfrac{1}{2}$ the second equation of (24) gives $c_1=-uc_0$, and the third equation then implies the stated condition $u^2=v$. Assuming this, the recursion again has a unique solution for each choice of $c_0$, and we obtain another double-valued solution to (21) near $x=q$. The form of these two solutions shows that (21) has an apparent singularity. If the relation $u^2=v$ does not hold, standard theory shows that the second solution to (21) has a logarithmic term, and the solutions then exhibit non-trivial monodromy around the point $x=q$, which is therefore not an apparent singularity. $\square$

### Stokes data

The analysis of the last section shows that the monodromy data of the Eq. (1) consists only of the Stokes data at the irregular singularity $x=\infty$. We now briefly recall how this is defined. A more detailed exposition of this material can be found for example in [2, Section 5]. The Stokes sectors are the sectors in $\mathbb {C}$ bounded by the asymptotic vertical directions of the quadratic differential

$$\begin{aligned} \hbar ^{-2}\cdot Q_0(x) dx^{\otimes 2}, \end{aligned}$$

which are easily seen to be the rays passing through the fifth roots of $-\hbar ^{2}$. General theory [29] shows that in each Stokes sector there is a unique subdominant solution to (1) up to scale, with the defining property that it exhibits exponential decay as $x\rightarrow \infty$ in the sector. Moreover, the subdominant solutions in neighbouring sectors are linearly independent.

Since the space of solutions to the Eq. (1) is a two-dimensional complex vector space, the subdominant solutions define a collection of five points of $\mathbb {P}^1$, well-defined up to the diagonal action of ${\text {PGL}}_2$, with the property that each consecutive pair of points is distinct. These points are naturally indexed by the Stokes sectors of the equation, and hence by the fifth roots of $\hbar ^2$. Choosing one such root we can identify this set with $\mathbb {Z}/5\mathbb {Z}$ and so obtain a point in the quotient space

25 $$\begin{aligned} V=\Big \{\psi :\mathbb {Z}/5\mathbb {Z}\rightarrow \mathbb {P}^1: \psi (i+1)\ne \psi (i)\text { for all }i\in \mathbb {Z}/5\mathbb {Z}\Big \} \Big / {\text {PGL}}_2, \end{aligned}$$

which is easily seen to be a two-dimensional complex manifold [18]. We call the resulting map

$$\begin{aligned} F(\hbar ):M\rightarrow V \end{aligned}$$

the monodromy map. Note however that this is a mild abuse of notation since $F(\hbar )$ really depends on a choice of fifth root of $\hbar ^2$. The map $F(\hbar )$ is holomorphic because the subdominant solutions vary holomorphically with parameters [19, 29].

#### Remark 2.2

There is an obvious action of the group $\mathbb {Z}/5\mathbb {Z}$ on the space *V* obtained by precomposing the map $\psi$ in (25) with the translations $i\mapsto i+j$ of $\mathbb {Z}/5\mathbb {Z}$. It is easy to check that it has exactly two fixed points, represented by the cyclically-ordered 5-tuples of points of $\mathbb {P}^1$ of the form $(0,1,\infty ,x,x+1)$, with $x\in \mathbb {C}$ a solution to the golden ratio equation $x^2+x-1=0$. One way to avoid the choice of fifth root of $\hbar ^2$ when defining the monodromy map $F(\hbar )$ is to consider it as taking values in the complex orbifold obtained by quotienting *V* by this action.

### Isomonodromy flow

The following result gives a pair of flows on the four-dimensional manifold $M$ along which the monodromy map $F(\hbar )$ is constant.

#### Proposition 2.3

For a fixed $\hbar \in \mathbb {C}^*$ the monodromy map $F(\hbar )$ is preserved by the flows

26 $$\begin{aligned}&-\frac{1}{\hbar }\frac{\partial }{\partial r}+\bigg (\frac{\partial }{\partial b}+\frac{1}{2p}\frac{\partial }{\partial p} +\frac{r}{2p^2} \frac{\partial }{\partial r}\bigg ),\end{aligned}$$

27 $$\begin{aligned}&\quad -\frac{2p}{\hbar } \frac{\partial }{\partial q}-\frac{3q^2+a}{\hbar }\frac{\partial }{\partial p}+\bigg (\frac{\partial }{\partial a}-q\frac{\partial }{\partial b}-\frac{r}{p}\frac{\partial }{\partial q}- \frac{r(3q^2+a)}{2p^2}\frac{\partial }{\partial p}-\frac{r^2}{2p^3} (3q^2+a) \frac{\partial }{\partial r}\bigg ).\nonumber \\ \end{aligned}$$

#### Proof

A straightforward calculation which we leave to the reader shows that the first flow (26) preserves the potential $Q(x,\hbar )$, and hence the Eq. (1). We defer the proof that the second flow preserves the monodromy map to the next subsection. $\square$

Note that the flows of Proposition 2.3 span a two-dimensional sub-bundle of the tangent bundle of $M$, which is everywhere transverse to the kernel of the derivative of the projection map $\pi :M\rightarrow S$. This is the condition that the sub-bundle defines an Ehresmann connection on this map. We call it the isomonodromy connection.

#### Remark 2.4

When $r=0$ the Eq. (1) reduces to the deformed cubic oscillator of [28], and the flow (27) becomes

28 $$\begin{aligned} \frac{da}{dt}=1, \qquad \frac{db}{dt}=-q, \qquad \frac{dq}{dt}=-\frac{2p}{\hbar }, \qquad \frac{dp}{dt}=-\frac{3q^2+a}{\hbar }. \end{aligned}$$

Let us briefly recall the well-known Hamiltonian description of this flow, and the link with Painlevé equations. Fix the parameter $\hbar \in \mathbb {C}^*$, and consider the space $\mathbb {C}^4$ with co-ordinates (*a*, *b*, *q*, *p*) equipped with the symplectic form

$$\begin{aligned} \omega =da\wedge db +\hbar \cdot dq\wedge dp. \end{aligned}$$

Then (28) is the flow defined by the Hamiltonian

$$\begin{aligned}H(a,b,q,p)=q^3+aq+b-p^2. \end{aligned}$$

Since $da/dt=1$ we can set $t=a$. The flow (28) then implies that

$$\begin{aligned} \hbar ^2 \cdot \frac{d^2q}{dt^2}=-2 \hbar \cdot \frac{dp}{dt}=6q^2+2t, \end{aligned}$$

which, after rescaling, becomes the first Painlevé equation.

### Proof of the isomondromy property

Let us complete the proof of Proposition 2.3. We must just show that the second flow (27) preserves the Stokes data.

#### Proof

Let us fix $\hbar \in \mathbb {C}^*$ and consider the potential $Q=Q(x)$ to be also a function of a variable $t\in \mathbb {C}$, in such a way that the derivative with respect to *t* gives the flow (27). The condition for the Stokes data to be constant [31] is the existence of an extended flat connection of the form

29 $$\begin{aligned} \nabla =d-\begin{pmatrix}0&{}\quad 1\\ Q(x,t)&{}\quad 0\end{pmatrix} dx - B(x,t) dt, \end{aligned}$$

with *B*(*x*, *t*) a meromorphic matrix-valued function. Let us make the ansatz

$$\begin{aligned} B(x,t)=\begin{pmatrix}-\tfrac{1}{2}A'&{}\quad A\\ A Q-\tfrac{1}{2}A''&{}\quad \tfrac{1}{2}A'\end{pmatrix}, \end{aligned}$$

for some function $A=A(x,t)$, where primes denote derivatives with respect to *x*. The flatness condition for the connection (29) then becomes

30 $$\begin{aligned} \frac{\partial ^3 A}{\partial x^3}-4Q \frac{\partial A}{\partial x}- 2 \frac{\partial Q}{\partial x} A + 2 \frac{\partial Q}{\partial t}=0, \end{aligned}$$

an equation which goes back at least to Fuchs. We now take $A=(x-q)^{-1}$. Writing out Eq. (30) gives

$$\begin{aligned} \frac{4}{(x-q)^2} Q(x) - \frac{2}{x-q} Q'(x) + 2{\dot{Q}}(x)-\frac{6}{(x-q)^4} =0, \end{aligned}$$

where dots denote differentiation with respect to *t*. In detail this is

$$\begin{aligned}&\frac{4}{\hbar ^2(x-q)^2} (x^3+ax+b) - \frac{2}{\hbar ^2 (x-q)} (3x^2+a) +\frac{2}{\hbar ^2}({\dot{a}}x+{\dot{b}}) +\frac{4}{\hbar (x-q)^2}\Big ( \frac{p}{x-q}+ r\Big ) \\&+\frac{2p}{\hbar (x-q)^3}+\frac{2}{\hbar }\Big ( \frac{{\dot{p}}}{x-q}+ {\dot{r}}\Big )+ \frac{2p{\dot{q}}}{\hbar (x-q)^2} +\frac{6}{(x-q)^4} +\frac{3{\dot{q}}}{(x-q)^3}-\frac{6}{(x-q)^4} \\&+\frac{3r}{p(x-q)^3}+\frac{r^2}{p^2(x-q)^2} -\frac{r{\dot{p}}}{p^2(x-q)}+\frac{{\dot{r}}}{p(x-q)}+\frac{r{\dot{q}}}{p(x-q)^2} -\frac{r^2 {\dot{p}}}{p^3}+\frac{r {\dot{r}}}{p^2}=0. \end{aligned}$$

The expression on the left-hand side of this equation is a rational function of *x*, with possible poles only at $x=q$ and $x=\infty$. To show that it is zero we consider the terms in the Laurent expansion at each of these points, which are

$$\begin{aligned}&(x-q)^{-3}: \quad \frac{4p}{\hbar }+\frac{2p}{\hbar }+3{\dot{q}}+\frac{3r}{p},\\&(x-q)^{-2}: \quad \frac{4}{\hbar ^2} (q^3+aq+b)+ \frac{4r}{\hbar } +\frac{2p {\dot{q}}}{\hbar }+\frac{r^2}{p^2} +\frac{r{\dot{q}}}{p}, \\&(x-q)^{-1}: \quad \frac{4}{\hbar ^2} (3q^2+a)-\frac{2}{\hbar ^2} (3q^2+a)+\frac{2{\dot{p}}}{\hbar }-\frac{r {\dot{p}}}{p^2} +\frac{{\dot{r}}}{p},\\&x^1 : \quad \frac{4}{\hbar ^2} -\frac{6}{\hbar ^2}+\frac{2}{\hbar ^2}{\dot{a}},\qquad x^0: \quad \frac{8q}{\hbar ^2}-\frac{6q}{\hbar ^2} + \frac{2}{\hbar ^2} {\dot{b}}+\frac{2{\dot{r}}}{\hbar }-\frac{r^2 {\dot{p}}}{p^3}+\frac{r {\dot{r}}}{p^2}. \end{aligned}$$

These are all easily checked to vanish under the given flow

$$\begin{aligned}&{\dot{a}}=1,\quad {\dot{b}}=-q,\quad {\dot{q}}=-\frac{2p}{\hbar }-\frac{r}{p}, \quad {\dot{p}}=-\frac{3q^2+a}{\hbar } -\frac{r(3q^2+a)}{2p^2},\\&{\dot{r}}=-\frac{r^2}{2p^3} (3q^2+a), \end{aligned}$$

which completes the proof. $\square$

## Periods and the abelian holonomy map

In this section we first consider the period co-ordinates $(z_1,z_2)$ on the space *S* and the relationship with the affine co-ordinates (*a*, *b*). This is a standard calculation with Weierstrass elliptic functions. We then consider the expression (16) from the introduction and explain its conceptual meaning in terms of the holonomy of abelian connections. The author learnt this interpretation from [24, Section 3].

### Weierstrass elliptic functions

In what follows we shall need some basic and well known properties of the Weierstrass elliptic functions. These functions depend on a choice of lattice

$$\begin{aligned} \Lambda =\mathbb {Z}\omega _1\oplus \mathbb {Z}\omega _2\subset \mathbb {C}. \end{aligned}$$

We assume the generators $\omega _i$ are ordered so that ${\text {Im}}(\omega _2/\omega _1)>0$. Proofs of the following claims can all be found for example in [32, Chapter 20], although the reader should note that the generators of $\Lambda$ are denoted there by $2\omega _i$.

The Weierstrass $\wp$-function is a meromorphic function of $u\in \mathbb {C}$ with double poles at each lattice point $\omega \in \Lambda$. It is even and doubly-periodic

$$\begin{aligned} \wp (-u)=\wp (u), \qquad \wp (u+\omega _i)=\wp (u), \end{aligned}$$

and satisfies the differential equation

$$\begin{aligned} \wp '(u)^2=4\wp (u)-g_2(\Lambda ) \wp (u)-g_3(\Lambda ), \end{aligned}$$

where $g_2(\Lambda ), g_3(\Lambda )\in \mathbb {C}$ are constants depending on the lattice $\Lambda$.

The Weierstrass $\zeta$-function is uniquely characterised by the properties

31 $$\begin{aligned} \zeta '(u) =-\wp (u), \qquad \zeta (-u)=-\zeta (u). \end{aligned}$$

It has simple poles at the lattice points. This function is not quite periodic but satisfies

32 $$\begin{aligned} \zeta (u+\omega _i)-\zeta (u)= \eta _i, \end{aligned}$$

where the quasi-periods $\eta _1,\eta _2\in \mathbb {C}^*$ satisfy the Legendre relation

33 $$\begin{aligned} \omega _2\eta _1-\omega _1 \eta _2 = 2{\pi } i. \end{aligned}$$

There is an addition formula

34 $$\begin{aligned} \zeta (u-v)-\zeta (u)+\zeta (v)=\frac{\wp '(u)+\wp '(v)}{2(\wp (u)-\wp (v))}. \end{aligned}$$

Finally, the Weierstrass $\sigma$-function is uniquely characterised by the relations

$$\begin{aligned} \frac{d}{du} \log \sigma (u)=\zeta (u),\qquad \lim _{u\rightarrow 0} \bigg (\frac{\sigma (u)}{u}\bigg )=1. \end{aligned}$$

It has the quasi-periodicity property

35 $$\begin{aligned} \sigma (u+\omega _i)=-\exp \big (\eta _i (u+\tfrac{1}{2}\omega _i)\big )\cdot \sigma (u), \end{aligned}$$

and has simple poles at the lattice points $\omega \in \Lambda$.

### Period map

Recall from the introduction the family of elliptic curves $X_s$ parameterised by the points $s\in S$. They are the projectivizations of the affine cubics

$$\begin{aligned} X_{s}^\circ =\big \{(x,y)\in \mathbb {C}^2: y^2=x^3+ax+b\big \}. \end{aligned}$$

As before we set $\Gamma _s=H_1(X_s,\mathbb {Z})$, and denote by

$$\begin{aligned} \langle -,-\rangle :\Gamma _s\times \Gamma _s\rightarrow \mathbb {Z}\end{aligned}$$

the skew-symmetric intersection form. We also consider the vector bundle $\pi :T\rightarrow S$ with fibres

$$\begin{aligned} T_s=H^1(X_s,\mathbb {C})= {\text {Hom}}_\mathbb {Z}(\Gamma _s,\mathbb {C})\cong \mathbb {C}^2. \end{aligned}$$

The Gauss-Manin connection defines a flat connection on this bundle. There is a holomorphic section $Z:S\rightarrow T$ defined by sending a class $\gamma \in \Gamma _s$ to

$$\begin{aligned} Z(s)(\gamma )=\int _{\gamma } \sqrt{Q_0(x)}\, dx=\int _{\gamma } y \, dx \in \mathbb {C}, \end{aligned}$$

which we call the period map. We claim that the covariant derivative of *Z* defines an isomorphism

$$\begin{aligned} \nabla (Z):\mathcal {T}_S \rightarrow T, \end{aligned}$$

between the holomorphic tangent bundle of *S* and the bundle *T*.

Let us express all this in co-ordinates. For this purpose, fix a base-point $s_0\in S$, and choose a basis

$$\begin{aligned} \Gamma _{s_0}=\mathbb {Z}\gamma _1\oplus \mathbb {Z}\gamma _2 \end{aligned}$$

satisfying $\langle \gamma _1,\gamma _2\rangle =1$. Extend this basis to nearby fibres $\Gamma _s$ using the Gauss-Manin connection. We obtain a local trivialization of the bundle $\pi :T\rightarrow S$

36 $$\begin{aligned} \big (\theta :\Gamma _s\rightarrow \mathbb {C}\big )\in T_s \mapsto (\theta _1,\theta _2)=\big (\theta (\gamma _1),\theta (\gamma _2)\big )\in \mathbb {C}^2, \end{aligned}$$

and the section *Z* becomes a pair of functions on *S*

37 $$\begin{aligned} z_i= \int _{\gamma _i} \sqrt{x^3+ax+b} \cdot dx. \end{aligned}$$

The claim is equivalent to the statement that these functions form a local system of co-ordinates on *S*. We check this by direct calculation in Lemma 3.1 below.

### Formula for the period map

For each point $s\in S$, we equip the elliptic curve $X_s$ with the global holomorphic one-form $\Omega$ which extends the form *dx*/2*y* on the affine piece $X_s^\circ$. The periods of this form

$$\begin{aligned} \omega _i=\int _{\gamma _i} \Omega =\int _{\gamma _i} \frac{dx}{2y} \in \mathbb {C}^* \end{aligned}$$

span a lattice $\Lambda _s=\mathbb {Z}\omega _1\oplus \mathbb {Z}\omega _2\subset \mathbb {C}$. The condition $\langle \gamma _1,\gamma _2\rangle =1$ ensures that ${\text {Im}}(\omega _2/\omega _1)>0$. The corresponding Weierstrass $\wp$-function defines a map

$$\begin{aligned} \mathbb {C}\setminus \Lambda _s\rightarrow X_s^\circ , \qquad u\mapsto (x,y)=\big (\wp (u), \tfrac{1}{2}\wp '(u)\big ), \end{aligned}$$

which extends to an isomorphism of complex manifolds

38 $$\begin{aligned} \mathbb {C}/\Lambda _s\cong X_s. \end{aligned}$$

Under this identification we have $\Omega =du$.

#### Lemma 3.1

The functions $(z_1,z_2)$ give local co-ordinates on *S*. There are equalities of tangent vectors on *S*

39 $$\begin{aligned}&\frac{\partial }{\partial a}=-\eta _1\frac{\partial }{\partial z_1} -\eta _2\frac{\partial }{\partial z_2},\qquad \frac{\partial }{\partial b}=\omega _1\frac{\partial }{\partial z_1} +\omega _2\frac{\partial }{\partial z_2}.\end{aligned}$$

40 $$\begin{aligned}&2{\pi } i\cdot \frac{\partial }{\partial z_1}=-\omega _2\frac{\partial }{\partial a} -\eta _2\frac{\partial }{\partial b},\qquad 2{\pi } i\cdot \frac{\partial }{\partial z_2}=\omega _1\frac{\partial }{\partial a} +\eta _1\frac{\partial }{\partial b}, \end{aligned}$$

where $\eta _1,\eta _2$ denote the quasi-periods of the Weierstrass $\zeta$-function associated to the lattice $\Lambda _s$.

#### Proof

Differentiating (37) gives

41 $$\begin{aligned}&\frac{\partial z_i}{\partial a} = \int _{\gamma _i} \frac{x\, dx}{2\sqrt{x^3+ax+b}}=\int _{\gamma _i} \frac{x\, dx}{2y}=\int _{\gamma _i} \wp (u) du = -\eta _i,\end{aligned}$$

42 $$\begin{aligned}&\frac{\partial z_i}{\partial b} = \int _{\gamma _i} \frac{dx}{2\sqrt{x^3+ax+b}}=\int _{\gamma _i} \frac{dx}{2y}=\int _{\gamma _i} du = \omega _i, \end{aligned}$$

and hence the relations (39). Inverting these using the Legendre relation (33) gives (40). $\square$

### Abelian holonomy map

Consider a point $(a,b,q,p,r)\in M$ and set $s=(a,b)\in S$. We denote by $w=(q,p)$ the corresponding point of the elliptic curve $X_s$. Using the parameterization (38) of $X_s$ we can write

43 $$\begin{aligned} w=(q,p)=\big (\wp (v), \tfrac{1}{2}\wp '(v)\big ). \end{aligned}$$

for some point $v\in \mathbb {C}+\Lambda _s$. Let us denote by $\infty \in X_s$ the point at infinity on the elliptic curve $X_s$. In terms of the parameterization (38) this corresponds to $0+\Lambda _s$. Let us introduce the meromorphic differential on $X_s$

44 $$\begin{aligned} \varpi (u) du=-\bigg (\frac{y+p}{x-q}+r\bigg )\frac{dx}{2y}=-\bigg (\frac{\wp '(u)+\wp '(v)}{2(\wp (u)-\wp (v))} +r\bigg )du. \end{aligned}$$

A simple calculation shows that $\varpi (u) du$ has simple poles at the points $\infty$ and *w*, with residues $+1$ and $-1$ respectively, and no other poles.

Consider the degree zero line bundle $L=\mathcal {O}_{X_s}(w-\infty )$ on $X_s$. In terms of the parameterization (38), the sections of *L* over an open subset are meromorphic functions *f*(*u*) having zeroes at the points $u\in \Lambda _s$, and at worst simple poles at the points $u\in v+\Lambda _s$. Note that for any such function *f*(*u*), the function $f'(u)-\varpi (u)f(u)$ has the same property. It follows that the formula

$$\begin{aligned} \nabla =d- \varpi (u) du \end{aligned}$$

defines a holomorphic connection on *L*. Computing the flat sections of $\nabla$ shows that the holonomy of this connection about a loop $\gamma$ in $X_s$ is given by multiplication by the expression

45 $$\begin{aligned} \xi (\gamma )=\exp \bigg (\int _{\gamma } \varpi (u) du\bigg )\in \mathbb {C}^*. \end{aligned}$$

Consider now the moduli space $\mathcal {M}_s$ of pairs $(L,\nabla )$ consisting of a line bundle *L* on the curve $X_s$, equipped with a holomorphic connection $\nabla$. Then $\mathcal {M}_s$ is an affine bundle over the space of degree zero line bundles ${\text {Pic}}^0(X_s)$ modelled on the vector space $\mathbb {C}=H^0(X_s,\omega _{X_s})$. The Riemann-Roch theorem shows that the line bundles $\mathcal {O}_{X_s}(w-\infty )$ for different points $w\in X_s$ are all distinct, and that all degree 0 line bundles on $X_s$ are of this form. Since these line bundles have only trivial automorphisms, the pairs $(L,\nabla )$ defined by different points (*q*, *p*, *r*) of the fibre $M_s=\pi ^{-1}(s)\subset M$ are all non-isomorphic. It follows that the map

46 $$\begin{aligned} A_s:M_s\rightarrow \mathcal {M}_s, \qquad A:(q,p,r)\mapsto (L,\nabla )=\big (\mathcal {O}_{X_s}(w-\infty ),d-\varpi (u)du\big ),\nonumber \\ \end{aligned}$$

is an open embedding. The condition $p\ne 0$ on the points of $M$ translates into the statement that the associated line bundle $L=\mathcal {O}_{X_s}(w-\infty )$ is not a spin bundle, that is, it does not satisfy $L^2\cong \mathcal {O}_X$. The image of the embedding $A_s$ is therefore precisely the set of pairs $(L,\nabla )$ for which the bundle *L* is non-spin.

For each point $s\in S$, the abelian Riemann-Hilbert correspondence shows that taking holonomy defines an isomorphism of complex manifolds ${\text {Hol}}:\mathcal {M}_s\rightarrow \mathbb {T}_s$. Pre-composing with the open embedding $A_s:M_s\hookrightarrow \mathcal {M}_s$ defines an open embedding $\Theta _s:M_s\hookrightarrow \mathbb {T}_s$ which sends a point $(q,p,r)\in M_s$ to the holonomy (45) of the pair $(L,\nabla )$ appearing in (46). Let us consider, as in the introduction, the bundle $\pi :\mathbb {T}\rightarrow S$ whose fibres are the cohomology groups

$$\begin{aligned} \mathbb {T}_s=H^1(\mathbb {T}_s,\mathbb {C}^*)={\text {Hom}}_\mathbb {Z}(\Gamma _s,\mathbb {C}^*)\cong (\mathbb {C}^*)^2. \end{aligned}$$

Then, taking the union of the maps $\Theta _s$ defines an open embedding $\Theta$, which fits into the diagramand induces the open embeddings $\Theta _s:M_s\hookrightarrow \mathbb {T}_s$ on the fibres. We call this map $\Theta$ the abelian holonomy map.

### Explicit formula

The bundle of tori $\pi :\mathbb {T}\rightarrow S$ is the quotient of the vector bundle $\pi :T\rightarrow S$ by the local system of lattices

47 $$\begin{aligned} \Gamma _s^\vee ={\text {Hom}}_\mathbb {Z}(\Gamma _s,\mathbb {Z})\subset T_s. \end{aligned}$$

Choosing a covariantly constant basis for the lattices $\Gamma _s$ as in Sect. 3.2 gives a local trivialisation

$$\begin{aligned}\big (\xi :\Gamma _s\rightarrow \mathbb {C}^*\big )\in \mathbb {T}_s \mapsto (\xi _1,\xi _2)=\big (\xi (\gamma _1),\xi (\gamma _2)\big )\in (\mathbb {C}^*)^2. \end{aligned}$$

The quotient map $p:T\rightarrow \mathbb {T}$ is expressed in co-ordinates by writing $\xi _i=\exp (\theta _i)$. Thus the pair $(\theta _1,\theta _2)$ of (36) can also be viewed as local co-ordinates on the bundle $\mathbb {T}$.

On the space $M$ we can take local co-ordinates (*a*, *b*, *q*, *r*). We can also express the co-ordinate *q* in terms of *v* using the parameterization (43) as before. Of course the Weierstrass function $\wp (v)$ depends implicitly on the lattice $\Lambda _s$, and hence on the variables (*a*, *b*).

#### Lemma 3.2

In the above co-ordinates the abelian holonomy map $\Theta$ is given by

48 $$\begin{aligned} \xi _i=\exp \big ( \eta _i v-r \omega _i-\omega _i \zeta (v)\big ), \end{aligned}$$

where $\zeta (v)$ denotes the Weierstrass zeta-function for the lattice $\Lambda _s$.

#### Proof

This is a direct computation which the author learnt from [24, Section 3]:

$$\begin{aligned} \theta _i=\log (\xi _i)=\int _{\gamma _i} \varpi (u) du = -\bigg [\log \frac{\sigma (u-v)}{\sigma (u)} + u\zeta (v)+ur\bigg ]_{\gamma _i}=\eta _i v- \omega _i(\zeta (v)+r), \end{aligned}$$

where we used the addition formula (34), and the quasi-periodicity property (35). Note that by construction the differential $\varpi (u) \, du$ has simple poles with integer residues, so the expression for $\theta _i$ is well-defined up to multiples of $2{\pi } i$, and the quantity $\xi _i=\exp (\theta _i)$ is therefore well-defined. $\square$

It will be convenient in what follows to introduce alternative local co-ordinates $(\theta _a,\theta _b)$ on the torus $\mathbb {T}_s$ by setting

49 $$\begin{aligned} \theta _i=-\eta _i\theta _a+\omega _i \theta _b. \end{aligned}$$

Using the Legendre relation (33), the inverse transformation is

50 $$\begin{aligned} 2{\pi } i \cdot \theta _a=-\omega _2\theta _1+\omega _1\theta _2, \qquad 2{\pi } i \cdot \theta _b=-\eta _2\theta _1+\eta _1\theta _2. \end{aligned}$$

In these co-ordinates (48) takes the simple form

51 $$\begin{aligned} \theta _a=-v=-\frac{1}{4}\int ^{(q,p)}_{(q,-p)} \frac{dx}{y}, \qquad \theta _b=-\zeta (v)-r=\frac{1}{4}\int ^{(q,p)}_{(q,-p)} \frac{x dx}{y}-r. \end{aligned}$$

It is easy to see that the integrals in (51) are well-defined providing we take an integration path which is invariant under the covering involution of $p:X_s\rightarrow \mathbb {P}^1$ defined by $(x,+y)\leftrightarrow (x,-y)$.

### Further remarks

We record here a few further comments on the abelian holonomy map which will be useful later.

#### Remark 3.3

It follows from the discussion in Sect. 3.4 that the complement of the image of the embedding $\Theta _s:M_s\hookrightarrow \mathbb {T}_s$ consists precisely of the holonomy of holomorphic connections on the four spin bundles on $X_s$. These correspond to the half-lattice points

$$\begin{aligned} \Big \{0,\tfrac{1}{2}\omega _1, \tfrac{1}{2}\omega _2, \tfrac{1}{2}(\omega _1+\omega _2)\Big \}\in v+\Lambda _s. \end{aligned}$$

Direct calculations shows that the resulting points of $\mathbb {T}_s$ have co-ordinates $\xi _i=\pm \exp ({r\omega _i})$, for some $r\in \mathbb {C}$, with the four possible choices of pairs of signs corresponding to the four spin bundles. For the non-trivial spin bundles this follows from (48) using the Legendre relation (33) and the identities

$$\begin{aligned} \zeta \big (\tfrac{1}{2}\omega _1\big )=\tfrac{1}{2}\eta _1, \qquad \zeta \big (\tfrac{1}{2}\omega _2\big )=\tfrac{1}{2}\eta _2, \qquad \zeta \Big (\tfrac{1}{2}(\omega _1+\omega _2)\Big )=\tfrac{1}{2}(\eta _1+\eta _2), \end{aligned}$$

which are easily derived from (31)–(32). On the other hand, a holomorphic connection on the trivial bundle $\mathcal {O}_{X_s}$ takes the form $d-r\, du$, where *d* denotes the trivial connection. The holonomy around the cycles $\gamma _i\in \Gamma _s$ is then given by multiplication by $\xi _i=\exp ({r\omega _i})$.

#### Remark 3.4

In the introduction we defined the map $\Theta$ by an expression

52 $$\begin{aligned} \xi (\gamma )=\exp \bigg (\int _{\gamma }\frac{-Q_1(x)\, dx}{2\sqrt{Q_0(x)}}\bigg ). \end{aligned}$$

The meromorphic differential on $X_s$ being integrated here

53 $$\begin{aligned} -\frac{Q_1(x)\, dx}{2\sqrt{Q_0(x)}}=-\bigg (\frac{p}{x-q} + r\bigg ) \frac{dx}{2y}=-\bigg (\frac{\wp '(v)}{2(\wp (u)-\wp (v))} + r\bigg ) du, \end{aligned}$$

has simple poles at the points $\pm v+\Lambda _s$ with residues ${\mp } \tfrac{1}{2}$. It follows that the integral of (53) against any homology class is well-defined only up to integer multiples of ${\pi }i$, and that the exponential (52) is therefore only well-defined up to sign. The difference between (53) and (44) is given by the form

$$\begin{aligned} \frac{dx}{2(x-q)}=\frac{\wp '(u)\, du}{2(\wp (u)-\wp (v))}. \end{aligned}$$

Since this differential is pulled back from ${\mathbb {P}}^1$ via the double cover $p{:} {X_{s}}\rightarrow {\mathbb {P}}^1$, its integral around any cycle (which is only well-defined up to integer multiples of ${\pi } i$) must in fact be an integer multiple of ${\pi } i$. Thus the expressions (45) and (52) agree up to sign.

#### Remark 3.5

There are two group actions on the space *M* which will be important later, and which are respected by the abelian holonomy map.

- There are involutions of the spaces *M* and $\mathbb {T}$ defined in local co-ordinates by
  $$\begin{aligned} (a,b,q,p,r)\leftrightarrow (a,b,q,-p,-r), \qquad (z_1,z_2,\theta _1,\theta _2)\leftrightarrow (z_1,z_2,-\theta _1,-\theta _2). \end{aligned}$$
  It follows from (51) and (49) that these are intertwined by the map $\Theta$.
- Consider the action of $\mathbb {C}^*$ on the space *M* for which the co-ordinates (*a*, *b*, *q*, *p*, *r*) are homogeneous of weights (4, 6, 2, 3, 1) respectively. Rescaling also the co-ordinate *x* on $\mathbb {C}\subset \mathbb {P}^1$ with weight 2, the formula (37) shows that the co-ordinates $(z_1,z_2)$ have weight 5, and formulae (44)–(45) that the co-ordinates $(\theta _1,\theta _2)$ have weight 0. The formulae (41)–(42) then show that $(\omega _i,\eta _i)$ have weight $(-1,1)$ respectively, and thus by (50) the co-ordinates $(\theta _a,\theta _b)$ have weights $(-1,1)$.

We shall need the following formula for the derivative of the map $\Theta$.

#### Lemma 3.6

The derivative of the abelian holonomy map with respect to the local co-ordinates (*a*, *b*, *q*, *r*) on $M$ and $(a,b,\theta _a,\theta _b)$ on $\mathbb {T}$ is given by

54 $$\begin{aligned}&\Phi _*\bigg (\frac{\partial }{\partial q}\bigg )= -\frac{1}{2p} \frac{\partial }{\partial \theta _a}+\frac{q}{2p}\frac{\partial }{\partial \theta _b}, \qquad \Phi _*\bigg (\frac{\partial }{\partial r}\bigg )= -\frac{\partial }{\partial \theta _b},\end{aligned}$$

55 $$\begin{aligned}&\Phi _*\bigg (\frac{\partial }{\partial a}\bigg )= \frac{\partial }{\partial a}+\kappa _1(v) \frac{\partial }{\partial \theta _a}-\kappa _2(v) \frac{\partial }{\partial \theta _b},\nonumber \\&\Phi _*\bigg (\frac{\partial }{\partial b}\bigg )= \frac{\partial }{\partial b}+\kappa _0(v) \frac{\partial }{\partial \theta _a}- \kappa _1(v) \frac{\partial }{\partial \theta _b},\end{aligned}$$

where we introduced the functions

56 $$\begin{aligned} \kappa _i(v)=\frac{1}{8}\int ^{(q,p)}_{(q,-p)}\frac{x^i dx}{y^3}=\int _{-v}^v \frac{\wp (u)^i\, du}{\wp '(u)^2}, \qquad i=0,1,2. \end{aligned}$$

#### Proof

The abelian holonomy map is given by the formulae (51), which can be viewed as integrals of multi-valued 1-forms on $\mathbb {P}^1$. Differentiating these gives the result. $\square$

#### Remark 3.7

As with (51), the integrals in (56) are well-defined providing the path of integration is invariant under $(x,+y)\leftrightarrow (x,-y)$. This results in meromorphic functions which are uniquely defined by the properties

$$\begin{aligned} \kappa '_i(v)=\frac{2\wp (v)^i}{\wp '(v)^2}, \qquad \kappa _i(-v)=-\kappa _i(v). \end{aligned}$$

By computing the derivative of $(\alpha +\beta \wp (v)+\gamma \wp ^2(v))/\wp '(v)$ as in (63) below, and comparing constants, it is not hard to write $\kappa _i(v)$ explicitly in terms of $\wp (v)$, $\zeta (v)$ and *v*. Since we will make no use of the resulting expressions, we refrain from writing out the details.

## The isomonodromy connection

In this section we combine the material from the previous sections to give proofs of Theorems 1.4 and 1.5. We first use the abelian holonomy map to transfer the pencil of isomonodromy connections to the bundle $\pi :\mathbb {T}\rightarrow S$. We then write the transferred pencil of non-linear connections in the natural co-ordinate system $(z_i,\theta _j)$. The resulting expressions show that these connections define what is called a Joyce structure in [5]. We then discuss the induced linear Joyce connection on *S*, and prove that its flat co-ordinates are (*a*, *b*).

### Rewriting the isomonodromy flow

We proved in the last section that the abelian holonomy map $\Theta :M\hookrightarrow \mathbb {T}$ is an open embedding, commuting with the projections to *S*. We can therefore use it to push-forward the isomonodromy connection of Proposition 2.3. The following result gives a Hamiltonian description of the resulting meromorphic Ehresmann connection.

#### Theorem 4.1

The push-forward of the isomonodromy connection along the open embedding $\Theta :M\hookrightarrow \mathbb {T}$ is spanned by vector fields of the form

57 $$\begin{aligned}&\frac{\partial }{\partial a} +\frac{1}{\hbar } \cdot \frac{\partial }{\partial \theta _a} +\frac{1}{2{\pi } i}\cdot \frac{\partial ^2 K}{\partial \theta _a \partial \theta _b}\cdot \frac{\partial }{\partial \theta _a}-\frac{1}{2\pi i}\cdot \frac{\partial ^2 K}{\partial \theta _a \partial \theta _a }\cdot \frac{\partial }{\partial \theta _b}, \end{aligned}$$

58 $$\begin{aligned}&\frac{\partial }{\partial b} + \frac{1}{\hbar } \cdot \frac{\partial }{\partial \theta _b} +\frac{1}{2\pi i}\cdot \frac{\partial ^2 K}{\partial \theta _b \partial \theta _b }\cdot \frac{\partial }{\partial \theta _a}-\frac{1}{2\pi i}\cdot \frac{\partial ^2 K}{\partial \theta _a \partial \theta _b }\cdot \frac{\partial }{\partial \theta _b}, \end{aligned}$$

with *K* a holomorphic function defined on the image of $\Theta$.

#### Proof

Making a trivial linear combination of the flows of Proposition 2.3, and leaving the variation of $p=p(a,b,q)$ implicit, the isomonodromy connection is generated by the vector fields

59 $$\begin{aligned}&-\frac{2p}{\hbar } \frac{\partial }{\partial q} -\frac{q}{\hbar } \frac{\partial }{\partial r} +\bigg (\frac{\partial }{\partial a}-\frac{r}{p}\frac{\partial }{\partial q} -\frac{r^2(3q^2+a)-qpr}{2p^3} \frac{\partial }{\partial r}\bigg ),\nonumber \\&-\frac{1}{\hbar }\frac{\partial }{\partial r}+\bigg (\frac{\partial }{\partial b} +\frac{r}{2p^2} \frac{\partial }{\partial r}\bigg ). \end{aligned}$$

Applying the derivative of $\Theta$ computed in Lemma 3.6 these become

$$\begin{aligned} \frac{1}{\hbar } \frac{\partial }{\partial \theta _a} + \frac{\partial }{\partial a}+ \mu \frac{\partial }{\partial \theta _a}- \nu \frac{\partial }{\partial \theta _b},\qquad \frac{1}{\hbar } \frac{\partial }{\partial \theta _b} + \frac{\partial }{\partial b}+ \uplambda \frac{\partial }{\partial \theta _a}- \mu \frac{\partial }{\partial \theta _b}, \end{aligned}$$

where the functions $\uplambda ,\mu ,\nu$ are defined on the image of $\Theta$ by

60 $$\begin{aligned} \Theta ^*(\uplambda )= \kappa _0(v), \quad \Theta ^*(\mu )=\kappa _1(v)+\frac{r}{2p^2}, \quad \Theta ^*(\nu )= \kappa _2(v)+\frac{qr}{p^2} -\frac{(3q^2+a)r^2}{2p^3}.\nonumber \\ \end{aligned}$$

Note that the inverse to the derivative in Lemma 3.6 satisfies

61 $$\begin{aligned} \Theta _*^{-1}\Big (\frac{\partial }{\partial \theta _a}\Big )= -2p \frac{\partial }{\partial q}-q\frac{\partial }{\partial r}, \qquad \Theta _*^{-1}\Big (\frac{\partial }{\partial \theta _b}\Big )= -\frac{\partial }{\partial r}. \end{aligned}$$

Define a holomorphic function *J* on the image of the open inclusion $\Theta :M\hookrightarrow \mathbb {T}$ by

62 $$\begin{aligned} \frac{1}{2{\pi } i} \cdot \Theta ^*(J)=-\frac{1}{4\Delta p} \big ( 2ap^2+3p(3b-2aq)r+(6aq^2-9bq+4a^2)r^2 - 2apr^3\big ),\nonumber \\ \end{aligned}$$

where we set $\Delta =4a^3+27b^2$. The defining relation $p^2=q^3+aq+b$ implies that

63 $$\begin{aligned} 2p\frac{d}{dq}\Big (\frac{\alpha q^2 +\beta q+\gamma }{p}\Big )=\alpha q-\beta +\frac{1}{p^2} \Big ( (2a\alpha -3\gamma )q^2+ (2a\beta +3 b\alpha )q+(3b\beta -a\gamma )\Big ).\nonumber \\ \end{aligned}$$

A slightly painful caluclation using (61) and the relation (63) repeatedly gives

$$\begin{aligned}&\frac{1}{2{\pi } i} \cdot \Theta ^*\Big (\frac{\partial J}{\partial \theta _a}\Big )=\frac{1}{\Delta }\bigg ( \frac{a^2}{2} +\frac{9bq}{4}- \frac{\big (9bq^2+2a^2q+6ab\big )r}{2p} +\frac{9br^2}{4}\bigg ) -\frac{r^2}{4p^2},\\&\frac{1}{2{\pi } i} \cdot \Theta ^*\Big (\frac{\partial ^2 J}{\partial \theta _a^2}\Big )=\frac{-1}{\Delta }\bigg (\frac{-2a^2q^2+3abq+9b^2}{2p} + a^2r\bigg )+\frac{qr}{p^2} - \frac{(3q^2+a)r^2}{2p^3},\\&\frac{1}{2{\pi } i} \cdot \Theta ^*\Big (\frac{\partial ^3 J}{\partial \theta _a^3}\Big )=-\frac{3ab}{2\Delta }-\frac{3q^2}{2p^2}-\frac{2r}{p} + \frac{3q(3q^2+a)r}{p^3}+\frac{6q r^2}{p^2}-\frac{3(3q^2+a)^2r^2}{2p^4}. \end{aligned}$$

On the other hand, applying the operators (61) to the expressions (60), and noting the overlap with the previous calculation, we easily obtain

64 $$\begin{aligned}&\Theta ^*\bigg (\frac{\partial \nu }{\partial \theta _a}\bigg )=-\frac{3q^2}{2p^2}- \frac{2r}{p}+\frac{3q(3q^2+a)r}{p^3}+\frac{6qr^2}{p^2}-\frac{3(3q^2+a)^2 r^2}{2p^4},\qquad \frac{\partial \uplambda }{\partial \theta _b }=0,\end{aligned}$$

65 $$\begin{aligned}&\Theta ^*\bigg (\frac{\partial \mu }{\partial \theta _a }\bigg )=-\frac{q}{p^2}+\frac{(3q^2+a)r}{p^3}=\Theta ^*\bigg (\frac{\partial \nu }{\partial \theta _b }\bigg ),\qquad \Theta ^*\bigg (\frac{\partial \uplambda }{\partial \theta _a }\bigg )=-\frac{1}{2p^2}=\Theta ^*\bigg (\frac{\partial \mu }{\partial \theta _b }\bigg ).\nonumber \\ \end{aligned}$$

Let us now define $K=J+C$, where

66 $$\begin{aligned} \frac{1}{2{\pi } i} \cdot C=\frac{1}{4\Delta } \big (ab\theta _a^3-2a^2\theta _a^2\theta _b-9b\theta _a\theta _b^2 +2a\theta _b^3\big ). \end{aligned}$$

Comparing with (64)–(65) we see that

67 $$\begin{aligned} 2{\pi } i \uplambda =\frac{\partial ^2 K}{\partial \theta _b^2}, \qquad 2{\pi } i \mu =\frac{\partial ^2 K}{\partial \theta _a\partial \theta _b}, \qquad 2{\pi } i \nu =\frac{\partial ^2 K}{\partial \theta _a^2}, \end{aligned}$$

up to the addition of functions independent of $\theta _a,\theta _b$. But these constants of integration must vanish because, by Remark 3.5(a), both sides of (67) are odd functions of the $\theta _a,\theta _b$ co-ordinates. $\square$

### Co-ordinate change

To pass from the statement of Theorem 4.1 to that of Theorem 1.4 we need to apply a change of variables. For this purpose, let us consider the following abstract problem. Suppose that *S* is a complex manifold equipped with a complex symplectic form $\omega$. Take a local co-ordinate system $(z_1,\cdots ,z_n)$ on *S* in which this form is constant, so that we can write

68 $$\begin{aligned} \omega =\sum _{i,j} \omega _{ij} \cdot dz_i \wedge dz_j, \end{aligned}$$

for some constant skew-symmetric matrix $\omega _{ij}$. The induced Poisson bracket on *S* is given in these co-ordinates by the inverse matrix

69 $$\begin{aligned} \{z_i,z_j\}=\epsilon _{ij}, \qquad \sum _j \epsilon _{ij}\cdot \omega _{jk}=\delta _{ik}. \end{aligned}$$

There is a natural co-ordinate system $(z_1,\ldots ,z_n,\theta _1,\ldots ,\theta _n)$ on the total space of the holomorphic tangent bundle $\mathcal {T}_S$ obtained by writing a tangent vector in the form $\sum _i \theta _i \cdot \frac{\partial }{\partial z_i}$. We are interested in systems of flows on $\mathcal {T}_S$ of the form

70 $$\begin{aligned} \frac{\partial }{\partial z_i}+ \frac{1}{\hbar } \frac{\partial }{\partial \theta _i} + \sum _{j,k} \epsilon _{jk} \frac{\partial ^2 J}{\partial \theta _i \partial \theta _j} \frac{\partial }{\partial \theta _k}. \end{aligned}$$

where $J:\mathcal {T}_S\rightarrow \mathbb {C}$ is some fixed holomorphic function.

Consider now some new co-ordinate system $(w_1,\ldots ,w_n)$ on *S* which is related to the first by a symplectomorphism, so that $\omega$ takes the same form (68). In the same way as before, we can consider the induced co-ordinates $(w_1,\ldots , w_n,\phi _1,\ldots ,\phi _n)$ on the tangent bundle $\mathcal {T}_S$. Given a holomorphic function $K:\mathcal {T}_S\rightarrow \mathbb {C}$ we can then consider the system of flows

71 $$\begin{aligned} \frac{\partial }{\partial w_i}+ \frac{1}{\hbar } \frac{\partial }{\partial \phi _i} + \sum _{j,k} \epsilon _{jk} \frac{\partial ^2 K}{\partial \phi _i \partial \phi _j} \frac{\partial }{\partial \phi _k}. \end{aligned}$$

We would like to know when the two flows (70) and (71) on the space $\mathcal {T}_S$ are equivalent, in the sense that they generate the same sub-bundle of the tangent bundle.

Let $\nabla$ denotes the flat, torsion-free, linear connection on the tangent bundle $\mathcal {T}_S$ whose flat co-ordinates are $(w_1,\ldots ,w_n)$, and define

72 $$\begin{aligned} C_{pqr}(z)=\omega \bigg (\nabla _{\frac{\partial }{\partial z_p}} \Big (\frac{\partial }{\partial z_q}\Big ),\frac{\partial }{\partial z_r}\bigg ). \end{aligned}$$

Note that the expression (72) is completely symmetric under permutation of the indices *p*, *q*, *r*. Indeed, the assumption that the symplectic form $\omega$ is constant in the co-ordinates $z_i$ and $w_j$, and hence is preserved by $\nabla$ gives

$$\begin{aligned} C_{pqr}(z)-C_{prq}(z)= & {} \omega \bigg (\nabla _{\frac{\partial }{\partial z_p}} \Big (\frac{\partial }{\partial z_q}\Big ),\frac{\partial }{\partial z_r}\bigg )+ \omega \bigg (\frac{\partial }{\partial z_q}, \nabla _{\frac{\partial }{\partial z_p}} \Big (\frac{\partial }{\partial z_r}\Big )\bigg )\\= & {} \frac{\partial }{\partial z_p}\omega \Big ( \frac{\partial }{\partial z_q},\frac{\partial }{\partial z_r}\Big )=0. \end{aligned}$$

On the other hand, the fact that $\nabla$ is torsion-free gives $C_{pqr}(z)=C_{qpr}(z)$.

#### Proposition 4.2

The two flows (70) and (71) define the same Ehresmann connection on the bundle $\pi :\mathcal {T}_S\rightarrow S$ precisely if

73 $$\begin{aligned} J(z_i,\theta _j)=K(w_i,\phi _j)-\frac{1}{6}\cdot \sum _{p,q,r}C_{pqr}(z_i) \cdot \theta _p \theta _q \theta _r. \end{aligned}$$

#### Proof

Take a point in the total space $\mathcal {T}_S$ with co-ordinates $(z_i,\theta _j)$ and $(w_i,\phi _j)$. Then

$$\begin{aligned} \sum _i \theta _i \frac{\partial }{\partial z_i}=\sum _j \phi _j \frac{\partial }{\partial w_j} \implies \phi _j=\sum _i \theta _i \frac{\partial w_j}{\partial z_i}. \end{aligned}$$

Changing co-ordinates on the space $\mathcal {T}_S$ from $(z_i,\theta _i)$ to $(w_j,\phi _j)$ therefore gives

74 $$\begin{aligned} \frac{\partial }{\partial z_i} = \sum _j \frac{\partial w_j}{\partial z_i} \frac{\partial }{\partial w_j} +\sum _{j,k}\theta _k \frac{\partial ^2 w_j}{\partial z_i \partial z_k}\frac{\partial }{\partial \phi _j}, \qquad \frac{\partial }{\partial \theta _i}=\sum _j \frac{\partial w_j}{\partial z_i} \frac{\partial }{\partial \phi _j}. \end{aligned}$$

Consider the following linear combination of the flows (71)

$$\begin{aligned} \sum _p \frac{\partial w_p}{\partial z_i} \frac{\partial }{\partial w_p}+ \frac{1}{\hbar } \sum _p \frac{\partial w_p}{\partial z_i}\frac{\partial }{\partial \phi _p} + \sum _{p, j,k} \epsilon _{jk} \frac{\partial ^2 K}{\partial \phi _p \partial \phi _j} \frac{\partial w_p}{\partial z_i} \frac{\partial }{\partial \phi _k} \end{aligned}$$

75 $$\begin{aligned} =\frac{\partial }{\partial z_i}-\sum _{j,k}\theta _k \frac{\partial ^2 w_j}{\partial z_i \partial z_k}\frac{\partial }{\partial \phi _j}+\frac{1}{\hbar }\frac{\partial }{\partial \theta _i} +\sum _{j,k} \epsilon _{jk} \frac{\partial ^2 K}{\partial \theta _i \partial \theta _j} \frac{\partial }{\partial \theta _k}, \end{aligned}$$

where we used the assumption that the change of co-ordinates from $z_i$ to $w_j$ is symplectic, which, using the second relation of (74), implies that for any function $f:\mathcal {T}_S\rightarrow \mathbb {C}$

$$\begin{aligned} \sum _{j,k} \epsilon _{jk} \frac{\partial f}{\partial \theta _j}\frac{\partial }{\partial \theta _k}= \sum _{j,k} \epsilon _{jk} \frac{\partial f}{\partial \phi _j}\frac{\partial }{\partial \phi _k}. \end{aligned}$$

The expressions (75) agree with (70) provided that

76 $$\begin{aligned} \sum _{j,k,q} \epsilon _{jq}C_{ijk}(z) \theta _k\frac{\partial }{\partial \theta _q}= \sum _{j,k} \theta _k \frac{\partial ^2 w_j}{\partial z_i \partial z_k} \frac{\partial }{\partial \phi _j} , \end{aligned}$$

for all indices *i*. But now we compute

$$\begin{aligned} C_{ijk}(z)=\omega \Bigg (\nabla _{\frac{\partial }{\partial z_i}} \Big (\frac{\partial }{\partial z_k}\Big ), \frac{\partial }{\partial z_j}\Bigg )=\omega \Bigg (\sum _{p} \frac{\partial ^2 w_p}{\partial z_i \partial z_k} \frac{\partial }{\partial w_p}, \frac{\partial }{\partial z_j}\Bigg )=\sum _{p,r} \omega _{rj} \frac{\partial ^2 w_p}{\partial z_i \partial z_k} \frac{\partial z_r}{\partial w_p}, \end{aligned}$$

and the identity (76) follows using the fact (69) that $\epsilon$ and $\omega$ are inverse matrices.$\square$

### The Joyce function

We can now use Proposition 4.2 to rewrite the flows of Theorem 4.1 in the co-ordinates $(z_i,\theta _j)$. This leads to the following statement.

#### Theorem 4.3

When written in the co-ordinates $(z_1,z_2,\theta _1,\theta _2)$, the push-forward of the isomonodromy flows (11)–(12) along the map $\Theta :M\rightarrow \mathbb {T}$ take the Hamiltonian form

77 $$\begin{aligned} \frac{\partial }{\partial z_i} + \frac{1}{\hbar } \cdot \frac{\partial }{\partial \theta _i} + \frac{\partial ^2 J}{\partial \theta _i\partial \theta _1}\cdot \frac{\partial }{\partial \theta _2}-\frac{\partial ^2 J}{\partial \theta _i\partial \theta _2}\cdot \frac{\partial }{\partial \theta _1}, \end{aligned}$$

where $J:\mathbb {T}\rightarrow \mathbb {C}$ is a meromorphic function with no poles on the locus $\theta _1=\theta _2=0$. When pulled-back to *M* using the abelian holonomy map it is given by the expression

78 $$\begin{aligned} \frac{1}{2{\pi } i}\cdot J\circ \Theta = -\frac{1}{4\Delta p}\cdot \big (2ap^2+3p(3b-2aq)r+(6aq^2-9bq+4a^2)r^2 - 2apr^3\big ).\nonumber \\ \end{aligned}$$

#### Proof

Let us define the rescaled co-ordinates

$$\begin{aligned} w_1=\sqrt{2{\pi } i}\cdot b , \qquad w_2=\sqrt{2\pi i}\cdot a. \end{aligned}$$

Using the expressions (39), and comparing with (50), the associated fibre co-ordinates of (74) are

$$\begin{aligned} \phi _1=\sqrt{2\pi i}\cdot \theta _b , \qquad \phi _2=\sqrt{2\pi i}\cdot \theta _a. \end{aligned}$$

Since $\epsilon _{12}=\langle \gamma _1,\gamma _2\rangle =1$, the Poisson and symplectic forms on *S* are

$$\begin{aligned} \{z_1,z_2\}=1, \qquad \omega =-dz_1\wedge dz_2. \end{aligned}$$

The relations (39) then ensure that

$$\begin{aligned} \omega =2\pi i \cdot da\wedge db= - dw_1\wedge dw_2. \end{aligned}$$

Making the trivial change of variables from $(a,b,\theta _a,\theta _b)$ to $(w_1,w_2,\phi _1,\phi _2)$ shows that the flows of Theorem 4.1 are given by the Eq. (71). Changing variables as in Proposition 4.2 then gives the flows in the form (77).

The formula (73) shows that on the image of $\Theta :M\hookrightarrow \mathbb {T}$, the required function *J* differs from the function *K* of Theorem 4.1 by an expression which is cubic in the $\theta _i$ co-ordinates. Moreover, by construction, the second derivatives of *J* with respect to the $\theta _i$ are single-valued functions on the image of $\Theta$. These two conditions uniquely determine *J*. Since the expression (62) has both the required properties, it follows that this coincides with the required function *J*.

We can view the space $M$ as an open subset of a larger space $M'$ obtained by dropping the condition $p\ne 0$. By its construction, the abelian holonomy map extends to an open embedding $\Theta :M'\hookrightarrow \mathbb {T}$, and the expression (62) clearly defines a meromorphic function on the open subset $\Theta (M')\subset \mathbb {T}$. As explained in Remark 3.3, the complement of this open subset is precisely the locus where $\xi _i=\exp (c\omega _i)$ for some $c\in \mathbb {C}$. So it remains to understand the behaviour of *J* at these points.

Let us work over a small open subset $U\subset S$. Let $0\in D\subset \mathbb {C}$ be a disc such that the punctured disc $D^\times =D\setminus \{0\}$ contains no points of $\Lambda _s$ for any $s=(a,b)\in U$. Consider the map

$$\begin{aligned} h:U\times D^{\times }\times \mathbb {C}\rightarrow M, \qquad (a,b,v,c)\mapsto \Big (a,b,\wp (v),\tfrac{1}{2}\wp '(v),-\zeta (v)-c\Big ). \end{aligned}$$

Using the formula (48) we see that the composition $g =\Theta \circ h$ sends (*a*, *b*, *v*, *c*) to the point of $\mathbb {T}$ with local co-ordinates $\theta _a=v$ and $\theta _b=c$. This shows that *g*, and hence also *h*, is an open embedding. Moreover *g* clearly extends to an open embedding $g:U\times D\times \mathbb {C}\rightarrow \mathbb {T}$. It will now be enough to show that the pull-back of the third derivatives (64)–(65) via the map *g* extend holomorphically over the locus $v=0$. But indeed, if we fix (*a*, *b*) and send *c* and *v* to 0, we have

$$\begin{aligned} q=\wp (v)=v^{-2} +\alpha v^2+ O(v^4), \qquad r= -v^{-1}-c+ \tfrac{1}{3}\alpha v^3 +O(v^5),\\p=\tfrac{1}{2}\wp '(v)=-v^{-3} + \alpha v +O(v^3), \qquad p^{-1}=-v^3 -\alpha v^7 +O(v^9). \end{aligned}$$

The equation $p^2=q^3+aq+b$ implies that $a+5\alpha =0$. It follows that, ignoring terms of total order at least 2 in *c* and *v*, the Joyce function satisfies

$$\begin{aligned} -\frac{4\Delta }{2\pi i}(J\circ \Theta )= & {} 2a(p-3qr+3q^2 p^{-1} r^2 + 2ap^{-1} r^2-r^3)+9b(r-qr^2 p^{-1}) \\&\sim 2a\big ( -v^{-3}+\alpha v -3(v^{-2}+\alpha v^2)(-v^{-1}-c+\tfrac{1}{3}\alpha v^3) +2a(-v^3)(v^{-2}) \\&+3(v^{-4}+2\alpha )(-v^3-\alpha v^7)(v^{-2}-2cv^{-1}+c^2-\tfrac{2}{3}\alpha v^2)\\&-(-v^{-3}-3cv^{-2}-3c^2 v^{-1} +\alpha v)\big ) \\&+9b\big (-v^{-1}-c-(v^{-2}+\alpha v^2)(v^{-2}+2cv^{-1}-\tfrac{2}{3}\alpha v^2)(-v^{3})\big ) \\&\sim 2a(-5\alpha v-2av)+9bc=2a^2 \theta _a +9b\theta _b. \end{aligned}$$

In particular, *J* is holomorphic along the locus $\theta _a=\theta _b=0$. $\square$

### Properties of the Joyce function

The general theory developed in [5] predicts some properties of the Joyce function, which it is interesting to check explicitly in the example being considered here.

#### Proposition 4.4

The Joyce function $J:\mathbb {T}\rightarrow \mathbb {C}$ of Theorem 4.3 is a meromorphic function with the following properties:

(i) it is an odd function in the $\theta _i$ co-ordinates:
  $$\begin{aligned} J(z_1,z_2,-\theta _1,-\theta _2)=-J(z_1,z_2,\theta _1,\theta _2); \end{aligned}$$
(ii) it is homogeneous of degree $-1$ in the co-ordinates $z_i$: for all $\uplambda \in \mathbb {C}^*$
  $$\begin{aligned} J(\uplambda z_1,\uplambda z_2,\theta _1,\theta _2)=\uplambda ^{-1} \cdot J(z_1,z_2,\theta _1,\theta _2); \end{aligned}$$
(iii) it satisfies the partial differential equation
  79 $$\begin{aligned} \frac{\partial ^2 J}{\partial \theta _i \partial z_j}-\frac{\partial ^2 J}{\partial \theta _j \partial z_i}=\sum _{p,q} \epsilon _{pq} \cdot \frac{\partial ^2 J}{\partial \theta _i \partial \theta _p} \cdot \frac{\partial ^2 J}{\partial \theta _j \partial \theta _q}. \end{aligned}$$

#### Proof

For part (i) consider the involution of Remark 3.5(a). It is immediate from (78) that the Joyce function $J(z_i,\theta _j)$ changes sign under this transformation. For part (ii) we consider the $\mathbb {C}^*$-action of Remark 3.5(b) which rescales the variables (*a*, *b*, *q*, *p*, *r*) with weights (4, 6, 2, 3, 1) respectively. Again, it is immediate from (78) that *J* has weight $-5$ for this action. Since the co-ordinates $z_i$ and $\theta _j$ have weights 5 and 0 respectively, this proves the claim.

For part (iii) note first that the isomonodromy connection on $\pi :M\rightarrow S$ is by definition the pull-back of the trivial connection on the projection map $\pi :V\times S\rightarrow S$ via the map

$$\begin{aligned}(F(\hbar ),\pi ):M\rightarrow V\times S.\end{aligned}$$

In particular it is flat. Writing out the zero curvature condition

$$\begin{aligned} \Bigg [\frac{\partial }{\partial z_1} + \frac{1}{\hbar } \frac{\partial }{\partial \theta _1} +\frac{\partial ^2 J}{\partial \theta _1\partial \theta _1}\frac{\partial }{\partial \theta _2}-\frac{\partial ^2 J}{\partial \theta _1\partial \theta _2}\frac{\partial }{\partial \theta _1}, \frac{\partial }{\partial z_2} + \frac{1}{\hbar } \frac{\partial }{\partial \theta _2} + \frac{\partial ^2 J}{\partial \theta _1\partial \theta _2}\frac{\partial }{\partial \theta _2}-\frac{\partial ^2 J}{\partial \theta _2\partial \theta _2}\frac{\partial }{\partial \theta _1}\Bigg ]=0 \end{aligned}$$

for the flows (77) shows that the partial derivative of (79) with respect to any co-ordinate $\theta _j$ vanishes. So in other words, the difference between the two sides of relation (79) is independent of the co-ordinates $\theta _j$.

To complete the proof it will be enough to show that the two sides of (79) both vanish on the locus $\theta _1=\theta _2=0$. By the calculation given in the proof of Theorem 4.3

$$\begin{aligned} \frac{1}{2\pi i}\cdot \frac{\partial J}{\partial \theta _a}\Big |_{\theta _1=\theta _2=0}= \frac{2a^2}{4\Delta } , \qquad \frac{1}{2\pi i}\cdot \frac{\partial J}{\partial \theta _b}\Big |_{\theta _1=\theta _2=0}=\frac{9b}{4\Delta }, \end{aligned}$$

so we find that

$$\begin{aligned} \frac{\partial ^2 J}{\partial \theta _a \partial b}\Big |_{\theta _1=\theta _2=0}- \frac{\partial ^2 J}{\partial \theta _b \partial a}\Big |_{\theta _1=\theta _2=0}=\frac{2\pi i}{4\Delta ^2} \Big ( 9b\frac{\partial \Delta }{\partial a}-2a^2 \frac{\partial \Delta }{\partial b}\Big )=0. \end{aligned}$$

It follows that the left-hand side of (79) vanishes on the locus $\theta _1=\theta _2=0$. But the right-hand side also vanishes because by part (i) *J* is an odd function of the $\theta _i$. $\square$

### The linear Joyce connection

An interesting output of the general theory developed in [5] is a flat, torsion-free connection on the tangent bundle of the space *S*, which we call the linear Joyce connection. To define it, note that by Theorem 4.3 the function *J* is holomorphic in a neighbourhood of the section of the map $\pi :\mathbb {T}\rightarrow S$ defined by setting $\theta _1=\theta _2=0$. Proposition 4.4(i) implies that the flows

$$\begin{aligned} \frac{\partial }{\partial z_i} + \frac{\partial ^2 J}{\partial \theta _i\partial \theta _1}\frac{\partial }{\partial \theta _2}-\frac{\partial ^2 J}{\partial \theta _i\partial \theta _2}\frac{\partial }{\partial \theta _1}, \end{aligned}$$

preserve this section, and it follows that their derivatives in the fibre directions are the flat sections of a linear connection on its normal bundle. This normal bundle can in turn be identified with the tangent bundle $\mathcal {T}_S$ via the map

$$\begin{aligned} \frac{\partial }{\partial \theta _1}\mapsto \frac{\partial }{\partial z_1}, \qquad \frac{\partial }{\partial \theta _2}\mapsto \frac{\partial }{\partial z_2}. \end{aligned}$$

The resulting connection on $\mathcal {T}_S$ is given explicitly by the formula

80 $$\begin{aligned} \nabla ^J_{\frac{\partial }{\partial z_i}}\Big (\frac{\partial }{\partial z_j}\Big )= -\sum _{k,l}\epsilon _{kl}\cdot \frac{\partial ^3 J}{\partial \theta _i \, \partial \theta _j \, \partial \theta _k}\Big |_{\theta =0} \cdot \frac{\partial }{\partial z_l}. \end{aligned}$$

For more details on the general definition and properties of the linear Joyce connection the reader can consult [5, Section 7]. The next result shows that, at least in the particular context treated in this paper, it is a very natural object.

#### Theorem 4.5

The linear Joyce connection $\nabla ^{ J}$ is the unique connection on *S* for which the co-ordinates (*a*, *b*) are flat.

#### Proof

The same argument used to derive the formula (80) shows that in the alternative co-ordinates $(w_i,\phi _j)$ used in the proof of Theorem 4.3, the linear Joyce connection is given by

81 $$\begin{aligned} \nabla ^J_{\frac{\partial }{\partial w_i}}\Big (\frac{\partial }{\partial w_j}\Big )= -\sum _{k,l}\epsilon _{kl}\cdot \frac{\partial ^3 K}{\partial \phi _i \, \partial \phi _j \, \partial \phi _k}\Big |_{\phi =0} \cdot \frac{\partial }{\partial w_l}. \end{aligned}$$

But applying the limiting argument used in the proof of Theorem 4.3 to the equations (64)–(65) shows that the third derivatives of the function *K* vanish along the locus $\phi _1=\phi _2=0$. Thus the right-hand side of (81) vanishes, and the functions $w_i$ are flat for the linear Joyce connection. $\square$

### The Joyce form

Let us introduce the vector field

$$\begin{aligned} E=z_1\frac{\partial }{\partial z_1}+z_2\frac{\partial }{\partial z_2}, \end{aligned}$$

and consider the endomorphism of $\mathcal {T}_S$ defined by

$$\begin{aligned} V(X)=\nabla ^J_X(E)-X. \end{aligned}$$

General theory developed in [5, Section 7] shows that the bilinear form

$$\begin{aligned} g(X,Y)=\omega \big (X,V(Y)\big ) \end{aligned}$$

is symmetric, and that both this form, and the operator *V*, are covariantly constant with respect to the linear Joyce connection. We call $g(-,-)$ the Joyce form. Note that when the Joyce form is non-degenerate, the resulting complex metric on *S* is necessarily flat, since the associated Levi-Civita connection is the linear Joyce connection $\nabla ^J$.

#### Proposition 4.6

The operator *V* is given by

$$\begin{aligned} V\bigg (\frac{\partial }{\partial a}\bigg )=-\frac{1}{5}\cdot \frac{\partial }{\partial a}, \qquad V\bigg (\frac{\partial }{\partial b}\bigg )=\frac{1}{5}\cdot \frac{\partial }{\partial b}, \end{aligned}$$

and the Joyce form is

$$\begin{aligned} g=\frac{2\pi i}{5} \cdot (da\otimes db+ db\otimes da). \end{aligned}$$

#### Proof

The properties of the $\mathbb {C}^*$-action used in the proof of Proposition 4.4 show that

$$\begin{aligned} E=z_1\frac{\partial }{\partial z_1}+z_2\frac{\partial }{\partial z_2} =\frac{4}{5} \cdot a\frac{\partial }{\partial a} + \frac{6}{5}\cdot b \frac{\partial }{\partial b}. \end{aligned}$$

Using Theorem 4.5 the claims then follow directly from the definitions. $\square$

##  BPS structures

In the last section it was explained that the isomonodromy connection for the family of deformed cubic oscillators gives an example of a Joyce structure in the sense of [5]. The remainder of the paper is devoted to showing how the monodromy map, and hence also the isomonodromy connection, can be derived from much simpler data called a variation of BPS structures, by solving an infinite-dimensional Riemann-Hilbert problem.

In this section we introduce BPS structures and their variations. These axiomatise the wall-crossing properties of Donaldson-Thomas (DT) invariants under deformations of stability parameters. We then introduce the Riemann-Hilbert problem associated to a BPS structure. For more details on the contents of this section we refer the reader to [4].

### BPS structures

The notion of a BPS structure was introduced in [4] to axiomatise the output of unrefined DT theory. It is a special case of Kontsevich and Soibelman’s notion of a stability structure in a graded Lie algebra [23]. In this paper we will only need to consider finite BPS structures, which allows us to make some significant expositional simplifications compared to the general treatment of [4].

#### Definition 5.1

A finite BPS structure consists of

- a finite-rank free abelian group $\Gamma \cong \mathbb {Z}^{\oplus n}$, equipped with a skew-symmetric form
  $$\begin{aligned} \langle -,-\rangle :\Gamma \times \Gamma \rightarrow \mathbb {Z}; \end{aligned}$$
- a homomorphism of abelian groups $Z:\Gamma \rightarrow \mathbb {C}$;
- a map of sets $\Omega :\Gamma \rightarrow \mathbb {Q};$

satisfying the following properties:

(i) $\Omega (-\gamma )=\Omega (\gamma )$ for all $\gamma \in \Gamma$, and $\Omega (0)=0$;
(ii) there are only finitely many classes $\gamma \in \Gamma$ such that $\Omega (\gamma )\ne 0$.^3^

A finite BPS structure $(\Gamma ,Z,\Omega )$ is called non-degenerate if the form $\langle -,-\rangle$ is non-degenerate, and integral if $\Omega (\gamma )\in \mathbb {Z}\subset \mathbb {Q}$ for all $\gamma \in \Gamma$.

### The twisted torus

Let $(\Gamma ,Z,\Omega )$ be a finite BPS structure as above. We introduce the algebraic torus

$$\begin{aligned} \mathbb {T}_+={\text {Hom}}_\mathbb {Z}(\Gamma ,\mathbb {C}^*)\cong (\mathbb {C}^*)^n, \end{aligned}$$

whose character lattice is $\Gamma$. We denote its co-ordinate ring by

$$\begin{aligned} \mathbb {C}[\mathbb {T}_+]=\mathbb {C}[\Gamma ]\cong \mathbb {C}[y_1^{\pm 1}, \ldots , y_n^{\pm n}], \end{aligned}$$

and write $y_\gamma \in \mathbb {C}[\mathbb {T}_+]$ for the character of $\mathbb {T}_+$ corresponding to an element $\gamma \in \Gamma$.

We also consider the associated torsor

$$\begin{aligned} \mathbb {T}_-= \big \{g:\Gamma \rightarrow \mathbb {C}^*: g(\gamma _1+\gamma _2)=(-1)^{\langle \gamma _1,\gamma _2\rangle } g(\gamma _1)\cdot g(\gamma _2)\big \}, \end{aligned}$$

called the twisted torus. The difference between $\mathbb {T}_+$ and $\mathbb {T}_-$ just has the effect of introducing signs into various formulae, and can safely be ignored at first reading.

The co-ordinate ring of $\mathbb {T}_-$ is spanned as a vector space by the functions

$$\begin{aligned} x_\gamma :\mathbb {T}_-\rightarrow \mathbb {C}^*, \qquad x_\gamma (g)=g(\gamma )\in \mathbb {C}^*, \end{aligned}$$

which we refer to as twisted characters. Thus

82 $$\begin{aligned} \mathbb {C}[\mathbb {T}_-]=\bigoplus _{\gamma \in \Gamma } \mathbb {C}\cdot x_\gamma , \qquad x_{\gamma _1}\cdot x_{\gamma _2}=(-1)^{\langle \gamma _1,\gamma _2\rangle }\cdot x_{\gamma _1+\gamma _2}. \end{aligned}$$

The torus $\mathbb {T}_+$ acts freely and transitively on the twisted torus $\mathbb {T}_-$ via

$$\begin{aligned} (f\cdot g)(\gamma )=f(\gamma )\cdot g(\gamma )\in \mathbb {C}^*, \quad f\in \mathbb {T}_+, \quad g\in \mathbb {T}_-. \end{aligned}$$

Choosing a base-point $g_0\in \mathbb {T}_-$ therefore gives a bijection

83 $$\begin{aligned} \theta _{g_0}:\mathbb {T}_+\rightarrow \mathbb {T}_-, \qquad f\mapsto f\cdot g_0. \end{aligned}$$

It is often convenient to choose a base-point in the finite subset

$$\begin{aligned} \big \{g:\Gamma \rightarrow \{\pm 1\}: g(\gamma _1+\gamma _2)=(-1)^{\langle \gamma _1,\gamma _2\rangle } g(\gamma _1)\cdot g(\gamma _2)\big \}\subset \mathbb {T}_-, \end{aligned}$$

whose points are called quadratic refinements of the form $\langle -,-\rangle$.

A class $\gamma \in \Gamma$ is called active if the corresponding BPS invariant $\Omega (\gamma )$ is nonzero. A ray $\mathbb {R}_{>0}\cdot z\subset \mathbb {C}^*$ is called active if it contains a point of the form $Z(\gamma )$ with $\gamma \in \Gamma$ an active class. Given a finite and integral BPS structure, we define for each ray $\ell =\mathbb {R}_{>0}\cdot z\subset \mathbb {C}^*$ a birational automorphism of the twisted torus $\mathbb {T}_-$ by the formula

84 $$\begin{aligned} \mathbb {S}(\ell )^*(x_\beta )=x_\beta \cdot \prod _{Z(\gamma )\in \ell } (1-x_{\gamma })^{\Omega (\gamma )\langle \gamma ,\beta \rangle }. \end{aligned}$$

The product is over all active classes $\gamma \in \Gamma$ such that $Z(\gamma )\in \ell$. The assumption that the BPS structure is finite ensures that this is a finite set.

### Variation of BPS structures

The behaviour of DT invariants under changes in stability parameters is controlled by the Kontsevich-Soibelman wall-crossing formula, which forms the main ingredient in the notion of a variation of BPS structures [4]. The condition that a family of BPS structures defines a variation is quite tricky to write down for general BPS structures, and the finiteness condition of Definition 5.1(ii) will not usually be preserved under wall-crossing. Nonetheless, for the very special class of BPS structures considered in this paper, it is possible to give a straightforward formulation of the wall-crossing formula using birational automorphisms of the twisted torus $\mathbb {T}_-$.

#### Definition 5.2

Let *S* be a complex manifold. A collection of finite, integral and non-degenerate BPS structures $(\Gamma _s,Z_s,\Omega _s)$ indexed by the points $s\in S$ forms a variation of BPS structures if

- the charge lattices $\Gamma _s$ form a local system of abelian groups, and the intersection forms $\langle -,-\rangle _s$ are covariantly constant;
- for any covariantly constant family of elements $\gamma _s\in \Gamma _s$, the central charges $Z_s(\gamma _s)\in \mathbb {C}$ vary holomorphically;
- consider an acute closed subsector $\Delta \subset \mathbb {C}^*$, and for each $s\in S$ define the anti-clockwise composition over active rays in $\Delta$
  85 $$\begin{aligned} \mathbb {S}_s(\Delta )=\prod _{\ell \subset \Delta } \mathbb {S}_s(\ell ); \end{aligned}$$
  then if $s\in S$ varies in such a way that the boundary rays of $\Delta$ are never active, the birational automorphism $\mathbb {S}_s(\Delta )$ of the twisted torus $\mathbb {T}_{s,-}$ is covariantly constant.

For part (c) note that the flat connection on the family of lattices $\Gamma _s$ induces an Ehresmann connection on the family of associated twisted tori $\mathbb {T}_{s,-}$, and we are asking that the birational automorphism $\mathbb {S}_s(\Delta )$ is constant with respect to this.

### Riemann-Hilbert problem

Let $(\Gamma , Z, \Omega )$ be a finite BPS structure with associated twisted torus $\mathbb {T}_-$. Given a ray $r\subset \mathbb {C}^*$ we consider the corresponding half-plane

86 $$\begin{aligned} \mathbb {H}_r=\{\hbar \in \mathbb {C}^*:\hbar =z\cdot v \text { with } z\in r\text { and }{\text {Re}}(v)>0\}\subset \mathbb {C}^*. \end{aligned}$$

We shall be dealing with meromorphic functions

$$\begin{aligned} X_r:\mathbb {H}_r\rightarrow \mathbb {T}_-. \end{aligned}$$

Composing with the twisted characters of $\mathbb {T}_-$ we can equivalently consider functions

$$\begin{aligned} X_{r,\gamma }:\mathbb {H}_r\rightarrow \mathbb {C}^*,\qquad X_{r,\gamma }(t)=x_\gamma (X_r(t)). \end{aligned}$$

The Riemann-Hilbert problem associated to the BPS structure $(\Gamma , Z, \Omega )$ depends on the additional choice of element $\xi \in \mathbb {T}_-$, which we refer to as the constant term. It reads as follows:

#### Problem 5.3

For each non-active ray $r\subset \mathbb {C}^*$ we seek a meromorphic function

$$\begin{aligned} X_r:\mathbb {H}_r\rightarrow \mathbb {T}_-, \end{aligned}$$

such that the following three conditions are satisfied:

1. if two non-active rays $r_1,r_2\subset \mathbb {C}^*$ form the boundary rays of a convex sector $\Delta \subset \mathbb {C}^*$ taken in clockwise order then
  $$\begin{aligned} X_{r_2}(\hbar )= \mathbb {S}(\Delta )( X_{r_1}(\hbar )), \end{aligned}$$
  as meromorphic functions of $\hbar \in \mathbb {H}_{r_-}\cap \mathbb {H}_{r_+}$, where $\mathbb {S}(\Delta )$ is as in (85);
2. for each non-active ray $r\subset \mathbb {C}^*$, and each class $\gamma \in \Gamma$, we have
  $$\begin{aligned} \exp (Z(\gamma )/\hbar )\cdot X_{r,\gamma }(\hbar ) \rightarrow \xi (\gamma ) \end{aligned}$$
  as $\hbar \rightarrow 0$ in the half-plane $\mathbb {H}_r$;
3. for each non-active ray $r\subset \mathbb {C}^*$, and each class $\gamma \in \Gamma$, there exists $k>0$ such that
  $$\begin{aligned} |\hbar |^{-k}< |X_{r,\gamma } (\hbar )|<|\hbar |^k, \end{aligned}$$
  for $$\hbar \in \mathbb {H}_r$$ satisfying $$|\hbar |\gg 0$$.

Note that in constrast to the treatment in [4] we have here allowed the functions $X_r$ to be meromorphic. The necessity of doing this was explained in [3]. It has the unfortunate effect that we lose any hope to prove uniqueness of solutions. It would be interesting to find a natural characterisation of the solutions to the Riemann-Hilbert problem constructed in this paper.

## Quadratic differentials

In this section we explain how the trajectory structure of the meromorphic quadratic differentials (7) define a variation of BPS structures on the space *S*. This can be described completely explicitly and corresponds to the Donaldson-Thomas theory of the A$_2$ quiver. We also discuss the WKB triangulation defined by a saddle-free quadratic differential. For more details on meromorphic quadratic differentials on Riemann surfaces we refer the reader to [7].

### Quadratic differentials

Let us consider a meromorphic quadratic differential

$$\begin{aligned} \phi (x)=\varphi (x) dx^{\otimes 2} \end{aligned}$$

on the Riemann surface $\mathbb {P}^1$ having a single pole of order 7 at the point $x=\infty$, and three simple zeroes. It is easy to see [7, Section 12.1] that any meromorphic quadratic differential of this type can be put in the form

87 $$\begin{aligned} \phi (x)=(x^3 + ax +b) dx^{\otimes 2} \end{aligned}$$

by applying an automorphism of $\mathbb {P}^1$. However it will not always be convenient to do this in what follows. Note also that care is required, since rescaling *x* by a fifth root of unity preserves the form of (87) but changes the pair (*a*, *b*).

Away from the zeroes and poles of $\phi (x)$ there is a distinguished local co-ordinate on $\mathbb {P}^1$

88 $$\begin{aligned} w(x)=\pm \int _*^{x} \sqrt{\varphi (u)} \, du \end{aligned}$$

in terms of which $\phi (x)$ takes the form $dw^{\otimes 2}$. Such a co-ordinate is uniquely determined up to transformations of the form $w\mapsto \pm w + c$. By definition, the horizontal foliation determined by $\phi (x)$ then consists of the arcs ${\text {Im}}(w)={\text {constant}}$. This foliation has singularities at the zeroes and poles of $\phi (x)$. Local computations [30] summarised in [7, Section 3.3] show that

(i) there are three horizontal arcs emanating from each of the three simple zeroes;
(ii) there are five tangent distinguished directions at the pole $x=\infty$, and an open neighbourhood $\infty \in U\subset \mathbb {P}^1$ such that all horizontal trajectories entering *U* approach $\infty$ along one of the distinguished directions.

Following [7, Section 6] we take the real oriented blow-up of the surface $\mathbb {P}^1$ at the point $\infty$ which is the unique pole of the quadratic differential $\phi (x)$. Topologically the resulting surface $\mathbb {S}$ is a disc. The distinguished directions at the pole determine a subset of five points $\mathbb {M}\subset \partial \mathbb {S}$ of the boundary of this disc; the pair $(\mathbb {S},\mathbb {M})$ is an example of a marked bordered surface. The horizontal foliation of $\mathbb {P}^1$ lifts to a foliation on the surface $\mathbb {S}$, with singularities at the points $\mathbb {M}\subset \partial \mathbb {S}$ and the zeroes of $\phi (x)$.

### Periods and saddle connections

Let us associate to a point $s\in S$ the quadratic differential

89 $$\begin{aligned} \phi _s(x)= Q_0(x) dx^{\otimes 2} = (x^3 + ax +b) dx^{\otimes 2}. \end{aligned}$$

There is a canonically associated double cover

90 $$\begin{aligned} p:X_s\rightarrow \mathbb {P}^1, \end{aligned}$$

branched at the zeroes and pole of $\phi _s(x)$, on which there is a well-defined global choice of square-root of $\phi _s(x)$. This is nothing but the projectivisation of the affine elliptic curve

$$\begin{aligned} X_{s}^\circ =\big \{(x,y)\in \mathbb {C}^2: y^2=x^3+ax+b\big \}. \end{aligned}$$

considered before. The square-root is the meromorphic differental *ydx*, which has a single pole at the point at infinity. There is a well-defined group homomorphism

91 $$\begin{aligned} Z_s:H_1(X_s,\mathbb {Z})\rightarrow \mathbb {C}, \qquad Z_s(\gamma )=\int _{\gamma } \sqrt{\phi _s(x)}\in \mathbb {C}. \end{aligned}$$

We shall call a point $s\in S$ generic if the image of $Z_s$ is not contained in a one-dimensional real subspace of $\mathbb {C}$.

A horizontal trajectory of $\phi _s(x)$ is said to be of finite-length if it never approaches the pole $x=\infty$. In our situation any such trajectory necessarily connects two distinct simple zeroes of $\phi _s(x)$, and is known as a saddle connection. The inverse image of a saddle connection under the double cover (90) is a cycle $\gamma$, which can be canonically oriented by insisting that $Z_s(\gamma )\in \mathbb {R}_{>0}$. This gives a well-defined homology class in $H_1(X_s,\mathbb {Z})$. See [7, Section 3.2] for more details.^4^

More generally we can consider trajectories of the differential $\phi _s(x)$ of some phase $\theta \in \mathbb {R}$. By definition these are arcs which make a constant angle $\pi \theta$ with the horizontal foliation. Alternatively one can view them as horizontal trajectories for the rescaled quadratic differential $e^{-2\pi i \theta }\cdot \phi _s(x)$. Once again, these finite-length trajectories $\gamma :[a,b]\rightarrow \mathbb {C}$ define homology classes in $H_1(X_s,\mathbb {Z})$, with the orientation convention being that $Z_s(\gamma )\in \mathbb {R}_{>0}\cdot e^{\pi i \theta }$.

### Walls and chambers

**Fig. 1:**
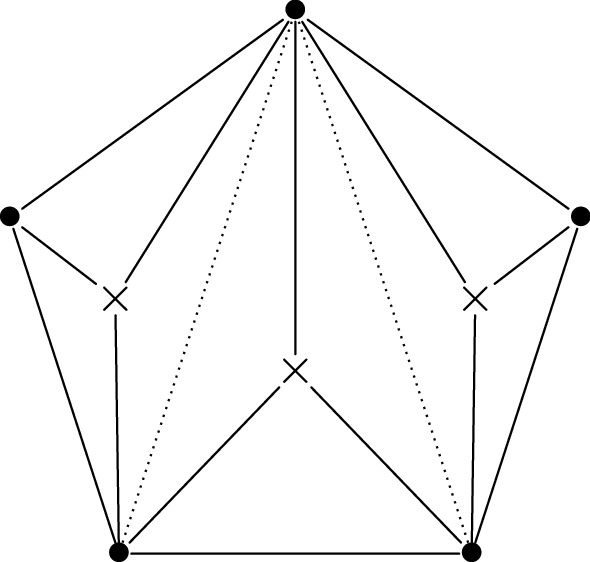
The separating trajectories of a saddle-free differential of the form (89)

Given a point $s\in S$, the quadratic differential $\phi _s(x)$ is said to be saddle-free if it has no finite-length horizontal trajectories. This is an open condition on the space *S*. As explained in [7, Section 3.4], the horizontal foliation of a saddle-free differential splits the surface $\mathbb {P}^1$ into a union of domains called horizontal strips and half-planes. In the present case we obtain five half-planes and two horizontal strips. The resulting trajectory structure on the blown-up surface $\mathbb {S}$ is illustrated in Fig. 1. The crosses denote zeroes of the differential, and the black dots are the points of $\mathbb {M}$.

Taking one trajectory from the interior of each horizontal strip defines a triangulation of the marked bordered surface $(\mathbb {S},\mathbb {M})$ called the WKB triangulation (see [7, Section 10.1] for details). In our case the result is the two dashed edges in Fig. 1. Note that there are exactly two internal edges, and all such triangulations differ by a rotation of the pentagon. As explained in [7, Section 3.6], each of the two horizontal strips contains a unique finite-length trajectory of some phase in the interval (0, 1), and the corresponding classes in $\gamma _i\in H_1(X_s,\mathbb {Z})$ determine a basis, whose elements are therefore indexed by the edges of the WKB triangulation.

### Associated BPS structures

There is a variation of BPS structures over the space *S* naturally associated to the family of quadratic differentials $\phi _s(x)$ defined by (89).

#### Definition 6.1

The BPS structure $(\Gamma _s,Z_s,\Omega _s)$ associated to a generic point $s\in S$ is defined as follows:

- the charge lattice is $\Gamma _s=H_1(X_s,\mathbb {Z})$ with its intersection form $\langle -,-\rangle$;
- the central charge $Z_s:\Gamma _s\rightarrow \mathbb {C}$ is the map (91);
- the BPS invariants $\Omega _s(\gamma )$ are either 0 or 1, with $\Omega _s(\gamma )=1$ precisely if the differential $\phi _s(x)$ has a finite-length trajectory of some phase whose associated homology class is $\gamma \in \Gamma _s$.

#### Remark 6.2

Condition (c) needs modification in the special case that the image of $Z_s$ is contained in a line $\mathbb {R}\cdot z$, and the correct definition of the invariants $\Omega _s(\gamma )$ at such non-generic points is quite subtle (see [20, Section 6.2]). This will play no role in what follows however, since what appears in the Riemann-Hilbert problem are the automorphisms $\mathbb {S}_s(\Delta )$ associated to sectors by the products (85), and by the wall-crossing formula these are locally constant, and hence determined by their values at generic points. See the last paragraph of the proof of Proposition 7.1 below.

Suppose that $s\in S$ corresponds to a saddle-free and generic differential $\phi _s$. As explained in the last subsection, the lattice $\Gamma _s$ then has a distinguished basis $(\gamma _1,\gamma _2)\subset \Gamma _s$, indexed by the edges of the WKB triangulation, which can be canonically ordered by insisting that $\langle \gamma _1,\gamma _2\rangle =1$. Set $z_i=Z(\gamma _i)\in \mathbb {C}$. The orientation conventions discussed above imply that ${\text {Im}}(z_i)>0$, and the genericity assumption is the statement that ${\text {Im}}(z_2/z_1)\ne 0$.

#### Proposition 6.3

Take a point $s\in S$, and let $(\Gamma _s,Z_s,\Omega _s)$ be the corresponding BPS structure. Suppose that the differential $\phi _s$ is saddle-free and generic, and let $(\gamma _1,\gamma _2)\subset \Gamma _s$ be the ordered basis as above. Define $z_i=Z(\gamma _i)\in \mathbb {C}^*$. Then the BPS invariants are as follows:

- if ${\text {Im}}(z_2/z_1)<0$ then $\Omega _s(\pm \gamma _1)=\Omega _s(\pm \gamma _2)=1$ with all others zero;
- if ${\text {Im}}(z_2/z_1)>0$ then $\Omega _s(\pm \gamma _1)=\Omega _s(\pm (\gamma _1+\gamma _2))=\Omega _s(\pm \gamma _2)=1$ with all others zero.

**Fig. 2:**
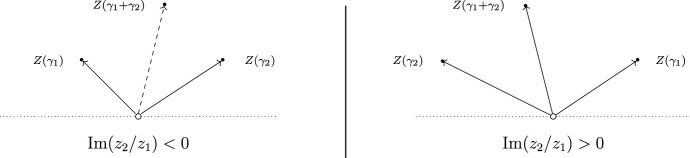
The BPS structures of Proposition 6.3

#### Proof

This could presumably be proved by direct analysis of the trajectory structure of the differentials $\phi _s$. Alternatively, it follows from the results of [7], together with the well-known representation theory of the A$_2$ quiver. In more detail, in the case of the marked bordered surface $(\mathbb {S},\mathbb {M})$ considered above, the CY$_3$ triangulated category $\mathcal {D}(\mathbb {S},\mathbb {M})$ appearing in [7] can be identified with the derived category $\mathcal {D}$ of the Ginzburg algebra of the A$_2$ quiver [7, Section 12.1]. The main result [7, Theorem 1.2] then shows that the differentials (89) define stability conditions on this category, and moreover, by [7, Theorem 1.4], the finite-length trajectories of the differential are in bijection with the stable objects of the associated stability condition. The result therefore follows from the easy and well known classification of stable representations of the A$_2$ quiver. Note that the basis $(\gamma _1,\gamma _2)\subset \Gamma _s$ correspond to the basis of the Grothendieck group $K_0(\mathcal {D})$ given by the classes of the vertex simples. The assumption $\langle \gamma _1,\gamma _2\rangle =1$ then corresponds to a quiver with a single arrow from vertex 2 to vertex 1. $\square$

In the situation of Proposition 6.3 there is a quadratic refinement $g\in \mathbb {T}_{s,-}$, defined by setting

$$\begin{aligned} g(\gamma _1)=g(\gamma _2)=-1, \end{aligned}$$

which is unique with the property that $g(\gamma )=-1$ for every active class $\gamma \in \Gamma _s$. We use this element and the map (83) to identify the twisted torus $\mathbb {T}_{s,-}$ with the standard torus $\mathbb {T}_{s,+}$. Under this identification the birational automorphism (84) becomes the birational automorphism of $\mathbb {T}_{s,+}$ defined by

$$\begin{aligned} \mathbb {S}(\ell )^*(y_\beta )=y_\beta \cdot \prod _{Z(\gamma )\in \ell } (1+y_{\gamma })^{\Omega (\gamma )\langle \gamma ,\beta \rangle }. \end{aligned}$$

Once we have Proposition 6.3, the fact that the BPS structures of Definition 6.1 form a variation of BPS structures comes down to the wall-crossing formula

92 $$\begin{aligned} C_{\gamma _1} \circ C_{\gamma _2} = C_{\gamma _2} \circ C_{\gamma _1+\gamma _2} \circ C_{\gamma _1}, \end{aligned}$$

where for each class $\alpha \in \Gamma _s$ we defined a birational automorphism $C_\alpha :\mathbb {T}_{s,+}\dashrightarrow \mathbb {T}_{s,+}$ by

$$\begin{aligned} C_\gamma ^*(y_\beta )=y_\beta \cdot (1+y_\gamma )^{\langle \gamma ,\beta \rangle }. \end{aligned}$$

This identity is familiar in cluster theory, and can be viewed as the semi-classical limit of the pentagon identity for the quantum dilogarithm.

## The solution to the Riemann-Hilbert problem

In this section we first introduce the Fock-Goncharov co-ordinates on the monodromy space *V*. These are birational maps to the torus $(\mathbb {C}^*)^2$ and depend on a choice of triangulation of the pentagon. We then prove that, when composed with these maps, the monodromy map for the deformed cubic oscillator gives a solution to the Riemann-Hilbert problem associated to the BPS structures of Sect. 6. In particular, this gives a proof of Theorem 1.6 from the introduction. Most of the content of this section is due to Gaiotto, Moore and Neitzke [17, Section 7].

### Fock-Goncharov co-ordinates

Let $(\mathbb {S},\mathbb {M})$ be a marked bordered surface of the kind appearing in Sect. 6, namely a disc with five marked points on the boundary. We call two points $p,q\in \mathbb {M}$ adjacent if they lie in the closure of the same connected component of $\partial \mathbb {S}\setminus \mathbb {M}$. We introduce the space

$$\begin{aligned} \mathcal {V}(\mathbb {S},\mathbb {M})=\big \{\psi :\mathbb {M}\rightarrow \mathbb {P}^1: \psi (p)\ne \psi (q)\text { for all adjacent points }p,q\in \mathbb {M}\big \}. \end{aligned}$$

**Fig. 3:**
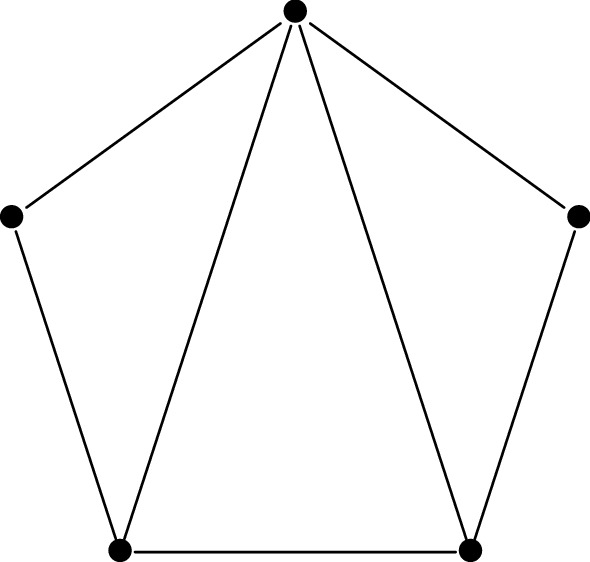
A triangulation of the marked bordered surface $(\mathbb {S},\mathbb {M})$

Let us now choose a triangulation *T* of the surface $(\mathbb {S},\mathbb {M})$ as in Fig. 3. In particular, the vertices of *T* are the points of $\mathbb {M}$. There are precisely five possible choices for *T*, all related by rotations. We denote by *E*(*T*) the set of internal edges of *T*: this set contains exactly two elements. Define

$$\begin{aligned} \mathcal {V}_T(\mathbb {S},\mathbb {M})\subset \mathcal {V}(\mathbb {S},\mathbb {M}) \end{aligned}$$

to be the open subset consisting of those points for which the elements $\psi (p)\in \mathbb {P}^1$ associated to the two ends of any edge of *T* are distinct.

For each internal edge $e\in E(T)$ there is a holomorphic map

93 $$\begin{aligned} X_e:\mathcal {V}_T(\mathbb {S},\mathbb {M})\rightarrow \mathbb {C}^* \end{aligned}$$

obtained by taking the cross-ratio

$$\begin{aligned} X_e={\text {CR}}(a_1,a_2,a_3,a_4)=\frac{(a_1-a_2)(a_3-a_4)}{(a_1-a_4)(a_2-a_3)}, \end{aligned}$$

of the four points $a_i=\psi (i)\in \mathbb {P}^1$ corresponding to the vertices of the two triangles adjoining the edge *e*. More precisely, the points $\psi (i)$ should be taken in anti-clockwise order starting with one of the two ends of *e*: there are two possible such orderings, but the two choices give the same value for the cross-ratio.

Combining the maps $X_e$ associated to the two internal edges of *T* gives a holomorphic map

$$\begin{aligned} X_T:\mathcal {V}_T(\mathbb {S},\mathbb {M})\rightarrow (\mathbb {C}^*)^{E(T)}\cong (\mathbb {C}^*)^2. \end{aligned}$$

The invariance property of the cross-ratio shows that this descends to the quotient space

$$\begin{aligned} V_T(\mathbb {S},\mathbb {M})=\mathcal {V}_T(\mathbb {S},\mathbb {M})/{\text {PGL}}_2\subset V(\mathbb {S},\mathbb {M})=\mathcal {V}(\mathbb {S},\mathbb {M})/PGL_2, \end{aligned}$$

and it is easy to see that the resulting map

94 $$\begin{aligned} X_T:V_T(\mathbb {S},\mathbb {M}) \rightarrow (\mathbb {C}^*)^{E(T)}\cong (\mathbb {C}^*)^2 \end{aligned}$$

is an isomorphism. The components of this map are called the Fock-Goncharov co-ordinates for the triangulation *T*.

### Solution to the Riemann-Hilbert problem

Take a point $s\in S$ and consider the corresponding quadratic differential (89). We would like to solve Problem 5.3 for the associated BPS structure $(\Gamma _s,Z_s,\Omega )$ of Definition 6.1. As explained in Sect. 6.4, there is a distinguished quadratic refinement of the form $\langle -,-\rangle _s$, and we can use the associated map (83) to identify the twisted torus $\mathbb {T}_{s,-}$ with the standard torus $\mathbb {T}_s=\mathbb {T}_{s,+}$. The Riemann-Hilbert problem then depends on a choice of a constant term $\xi \in \mathbb {T}_{s}$, and involves constructing meromorphic maps

95 $$\begin{aligned} X_r:\mathbb {H}_r\rightarrow \mathbb {T}_{s} \end{aligned}$$

for all non-active rays $r\subset \mathbb {C}^*$, where $\mathbb {H}_r$ is the half-plane defined in (86).

Let us assume first that the chosen point $\xi \in \mathbb {T}_{s}$ lies in the image of the abelian holonomy map $\Theta _s:M_s\rightarrow \mathbb {T}_s$, so that we can write $\xi _s=\Theta _s(m)$ for some point $m\in M_s$. We will construct a suitable map (95) by sending $\hbar \in \mathbb {H}_r$ to the Fock-Goncharov co-ordinates of the monodromy of the deformed cubic oscillator (1)–(2) defined by the point $m\in M$. More precisely, we will take the Fock-Goncharov co-ordinates defined by the WKB triangulation of the quadratic differential

96 $$\begin{aligned} \uplambda ^{-2} \cdot Q_0(x) dx^{\otimes 2}= \uplambda ^{-2}\cdot (x^3+ax+b)\cdot dx^{\otimes 2}, \end{aligned}$$

where $\uplambda \in r$ is an arbitrary point of the given ray. Note that the assumption that the ray $r\subset \mathbb {C}^*$ is non-active is equivalent to the statement that the differential (96) is saddle-free for $\uplambda \in r$.

One confusing point requires a little care. For each $\uplambda \in \mathbb {C}^*$ let us denote by $(\mathbb {S}(\uplambda ),\mathbb {M}(\uplambda ))$ the marked bordered surface determined by the rescaled differential (96). We can always take the underlying surface $\mathbb {S}(\uplambda )$ to be the unit disc in $\mathbb {C}$, and the marked points $\mathbb {M}(\uplambda )$ are then positive real multiples of the fifth roots of $\uplambda ^2$ (see for example [2, Section 3.2]). Given a ray $r\subset \mathbb {C}^*$, we will also use the notation $(\mathbb {S}(r),\mathbb {M}(r))$ for the marked bordered surface corresponding to an arbitrary point $\uplambda \in r$. It is important to note that if two rays $r_1,r_2\subset \mathbb {C}^*$ lie in the same half-plane then there is a canonical identification between the two surfaces $(\mathbb {S}(r_i),\mathbb {M}(r_i))$. In concrete terms, this is because the fifth root function is single-valued on any given half-plane.

Returning to our non-active ray $r\subset \mathbb {C}^*$, we can consider the associated WKB triangulation $T(r)$ of the marked bordered surface $(\mathbb {S}(r),\mathbb {M}(r))$. Since the internal edges of $T(r)$ are labelled by basis elements of the group $\Gamma _s$, the map (94) can be interpreted as a birational isomorphism

97 $$\begin{aligned} X_{T(r)}:V(\mathbb {S}(r),\mathbb {M}(r)) \dashrightarrow \mathbb {T}_+. \end{aligned}$$

On the other hand, as in [2, Section 5.3], the Stokes sectors of the Eq. (7) are in natural bijection with the points of $\mathbb {M}(\hbar )$. As discussed above, since $\hbar \in \mathbb {H}_r$, there is a canonical identification between the surfaces $(\mathbb {S}(r),\mathbb {M}(r))$ and $(\mathbb {S}(\hbar ),\mathbb {M}(\hbar ))$. We can then compose the monodromy map

$$\begin{aligned} F:\mathbb {H}_r\rightarrow V(\mathbb {S}(r),\mathbb {M}(r)), \qquad \hbar \mapsto F(\hbar )(m), \end{aligned}$$

with the map (97) to obtain the required map $X_r:\mathbb {H}_r\rightarrow \mathbb {T}_s$. We now proceed to check the conditions (RH1) – (RH3) of Problem 5.3.

### Jumping

Let us start with the jumping condition (RH1). Take a point $s\in S$ and let $\ell \subset \mathbb {C}^*$ be an active ray for the corresponding BPS structure $(\Gamma _s,Z_s,\Omega _s)$. Consider non-active rays $r_-$ and $r_+$ which are small anti-clockwise and clockwise deformations of the ray $\ell$. We can identify the marked bordered surfaces $(\mathbb {S}(r_{\pm }), \mathbb {M}(r_\pm ))$ associated to the rays $r_\pm$ with the surface $(\mathbb {S}(\ell ),\mathbb {M}(\ell ))$ as above, and hence also identify the spaces $V(r_\pm )$ with the fixed space $V(\ell )$. Let $T_\pm =T(r_\pm )$ be the WKB triangulations of the surface $(\mathbb {S}(\ell ),\mathbb {M}(\ell ))$ defined by the non-active rays $r_\pm$, and let $X_{T_\pm }:V(\ell )\dashrightarrow \mathbb {T}$ be the associated Fock-Goncharov co-ordinates.

#### Proposition 7.1

The two systems of co-ordinates are related by

$$\begin{aligned} X_{T_+}=\mathbb {S}(\ell )\circ X_{T_-} . \end{aligned}$$

#### Proof

Suppose first that $s\in S$ is generic. For $\uplambda \in \ell$ the differential (96) has a unique saddle connection, and the WKB triangulations $T_\pm$ for the saddle-free differentials (96) corresponding to $\uplambda _\pm \in r_\pm$ differ by a flip in a single edge. This situation is discussed in detail in [7, Section 10.3].

**Fig. 4:**
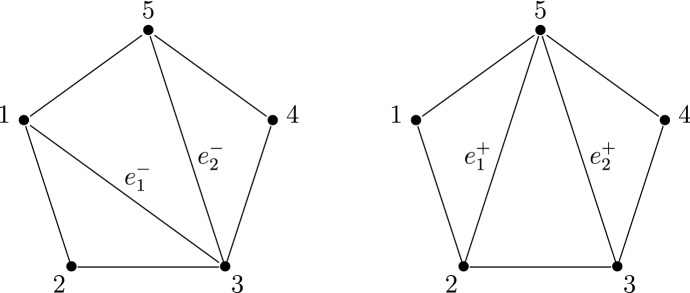
Flipping the triangulation: first case

**Fig. 5:**
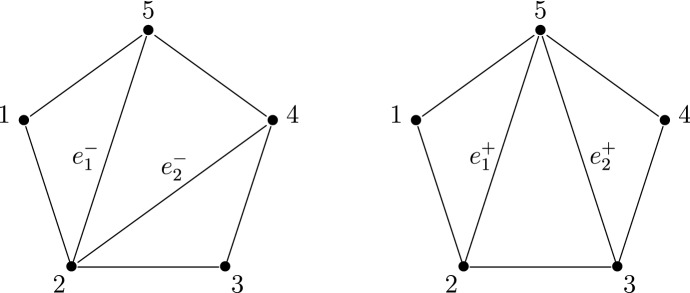
Flipping the triangulation: second case

Without loss of generality we can assume that the triangulation $T_+$ is as in Fig. 3. There are two cases, depending on which edge of the triangulation is being flipped. These are illustrated in Figs. 4 and 5. We label the vertices of the pentagon in clockwise cyclic order as shown. In each case, the left-hand picture illustrates $T_-$, and the right-hand picture is $T_+$. The two edges $e^+_1,e^+_2$ of the triangulation $T_+$ are labelled by classes $\gamma _1,\gamma _2\in \Gamma$. Since $e_1^+,e_2^+$ appear as adjacent edges in clockwise order in the unique triangle of $T_+$ which contains them both, the sign correction to [7, Lemma 10.3] mentioned in the proof of Proposition 6.3 shows that $\langle \gamma _1,\gamma _2\rangle =1$. Let us now consider the two cases in turn.

In the first case, illustrated in Fig. 4, the edge $e_1^+$ is being flipped. According to [7, Proposition 10.4], the edges $e^-_1,e^-_2$ are labelled by the classes $-\gamma _1,\gamma _1+\gamma _2$. The Fock-Goncharov co-ordinates are

$$\begin{aligned} X^+_1=X_{T_+}^*(y_{\gamma _1})={\text {CR}}(a_5,a_1,a_2,a_3), \qquad X^+_2=X_{T_+}^*(y_{\gamma _2})={\text {CR}}(a_5,a_2,a_3,a_4), \end{aligned}$$

on the right, whereas on the left we have

$$\begin{aligned} X^-_1= & {} X_{T_-}^*(y_{-\gamma _1})={\text {CR}}(a_1,a_2,a_3,a_5)=(X^+_1)^{-1}, \\ X^-_2= & {} X_{T_-}^*(y_{\gamma _1+\gamma _2})={\text {CR}}(a_5,a_1,a_3,a_4)=X^+_2\cdot \big (1+(X^+_1)^{-1}\big )^{-1}, \end{aligned}$$

where we used the easily-checked identity

$$\begin{aligned} {\text {CR}}(a_5,a_1,a_3,a_4)={\text {CR}}(a_5,a_2,a_3,a_4)\cdot \big (1+{\text {CR}}(a_5,a_1,a_2,a_3)^{-1}\big )^{-1}. \end{aligned}$$

Thus we have

$$\begin{aligned} X_{T_+}^*(y_{\gamma _1})=X_{T_-}^*(y_{\gamma _1}), \qquad X_{T_+}^*(y_{\gamma _2})=X_{T_-}^*\big (y_{\gamma _2}(1+y_{\gamma _1})\big ). \end{aligned}$$

Consider the central charges $Z_\pm =\uplambda _\pm \cdot Z_s$ with $\uplambda _\pm \in r_\pm$. By definition of the classes $\gamma _i\in \Gamma$ associated to the triangulation $T_+$ we have ${\text {Im}}Z_+(\gamma _1)>0$. Since the rotation from $\uplambda \in r_-$ to $\uplambda \in r_+$ is clockwise, the central charges $\uplambda ^{-1}\cdot Z(\gamma )$ rotate anti-clockwise, and it follows that for $\uplambda \in \ell$ the central charge $\uplambda ^{-1}\cdot Z(\gamma _1)$ lies on the positive real axis. Thus the corresponding wall-crossing automorphism $\mathbb {S}(\ell )=C_{\gamma _1}$ satisfies

$$\begin{aligned} \mathbb {S}(\ell )^*(y_{\gamma _1})=y_{\gamma _1}, \qquad \mathbb {S}(\ell )^*(y_{\gamma _2})=y_{\gamma _2}\cdot (1+y_{\gamma _1}), \end{aligned}$$

and we therefore conclude that $X_{T_+}^*=X_{T_-}^*\circ \mathbb {S}(\ell )^*$ as required.

In the second case, illustrated in Fig. 5, the edge $e_2^+$ is being flipped. This time [7, Proposition 10.4] shows that the edges $e^-_1,e^-_2$ are labelled by the classes $\gamma _1, -\gamma _2$. The Fock-Goncharov co-ordinates on the right are as before. On the left they are

$$\begin{aligned} X^-_1= & {} X_{T_-}^*(y_{\gamma _1})={\text {CR}}(a_5,a_1,a_2,a_4)=X_1^+\cdot (1+(X_2^+)), \\ X^-_2= & {} X_{T_-}^*(y_{-\gamma _2})={\text {CR}}(a_4,a_5,a_2,a_3)=(X_2^+)^{-1}, \end{aligned}$$

where we used

$$\begin{aligned} {\text {CR}}(a_5,a_1,a_2,a_4)={\text {CR}}(a_5,a_1,a_2,a_3)\cdot \big (1+{\text {CR}}(a_5,a_2,a_3,a_4)\big ). \end{aligned}$$

Thus we have

$$\begin{aligned} X_{T_+}^*(y_{\gamma _1})=X_{T_-}^*\big ( y_{\gamma _1}(1+y_{\gamma _2})^{-1}\big ),\qquad X_{T_+}^*(y_{\gamma _2})=X_{T_-}^*(y_{\gamma _2}). \end{aligned}$$

This time the wall-crossing automorphism $\mathbb {S}(\ell )=C_{\gamma _2}$ is given by

$$\begin{aligned} \mathbb {S}(\ell )^*(y_{\gamma _1})=y_{\gamma _1}\cdot (1+y_{\gamma _2})^{-1},\qquad \mathbb {S}(\ell )^*(y_{\gamma _2})=y_{\gamma _2}, \end{aligned}$$

so we again find that $X_{T_+}^*=X_{T_-}^*\circ \mathbb {S}(\ell )^*$.

Consider now the case when $s\in S$ is not generic. The corresponding BPS structure has exactly two active rays $\pm \ell$. Let us deform the point $s\in S$ to a nearby generic point $t\in S$. Under this deformation the ray $\ell$ splits into two or three rays $\ell _i$ as in Fig. 2, but for *t* close enough to *s* these rays $\ell _i$ will be contained in the sector bounded by the non-active rays $r_\pm$. The triangulations associated to the rays $r_\pm$ do not change under the deformation, and the wall-crossing formula (85) shows that the automorphism $\mathbb {S}_s(\ell )$ is the clockwise composition of the automorphisms $\mathbb {S}_t(\ell _i)$. The result for the non-generic point $s\in S$ now follows by applying the same result for the generic point $t\in S$ to each of the rays $\ell _i$. $\square$

### Behaviour as $\hbar \rightarrow 0$

To verify condition (RH2) of Problem 5.3 we must show that the map

$$\begin{aligned} X_r:\mathbb {H}_r\rightarrow \mathbb {T}_s, \qquad X_r(\hbar )=X_{T(r)}(F(\hbar )(m)), \end{aligned}$$

has the correct asymptotics as $\hbar \rightarrow 0$. As explained above, given an edge *e* of the WKB triangulation for the differential (96), there is a corresponding class $\gamma _e\in \Gamma _s$ defined by the saddle connection crossing the associated horizontal strip. The statement we want is that

$$\begin{aligned} X_r(\gamma _e)(\hbar )\sim \exp (-Z(\gamma _e)/\hbar ) \cdot \xi (\gamma _e), \end{aligned}$$

as $\hbar \rightarrow 0$ in the half-plane $\mathbb {H}_r$. To simplify matters a little, we can, by applying the $\mathbb {C}^*$ action on $M$ used in the proof of Proposition 4.4, assume that the ray $r=\mathbb {R}_{>0}$ is the positive real axis, and hence that the differential $\phi _s$ is saddle-free.

Let us then state the required result as concretely as possible. Consider a deformed cubic oscillator of the form (1)–(2), and assume that the corresponding quadratic differential $Q_0(x) dx^{\otimes 2}$ on $\mathbb {C}$ is saddle-free. The horizontal trajectory structure of this differential then defines a WKB triangulation of the regular pentagon with vertices at the fifth roots of unity. Moreover, each of the two edges $e_i$ of this triangulation *T* is naturally labelled by a class $\gamma _{i}$ in the homology group $H_1(X_s,\mathbb {Z})$ of (10). We set

$$\begin{aligned} z_i=\int _{\gamma _i} \sqrt{Q_0(x)} \, dx\in \mathbb {C}, \qquad \xi _i=\exp \bigg (\int _{\gamma _i} \frac{-Q_1(x)dx}{2\sqrt{Q_0(x)}}\bigg )\in \mathbb {C}^*. \end{aligned}$$

By definition of the orientation of the classes $\gamma _i$ we have ${\text {Im}}(z_i)>0$.

When $\hbar \in \mathbb {R}_{>0}$ the Stokes sectors for our Eq. (1) are centered on the rays spanned by the fifth roots of unity. Thus for all ${\text {Re}}(\hbar )>0$ we can continuously identify the Stokes sectors with the vertices of the triangulation *T*. Using this identification, we let $X_i(\hbar )$ denote the Fock-Goncharov co-ordinate corresponding to the edge $e_i$ of the triangulation *T*, for the point of the monodromy manifold defined by the subdominant solutions of the Eq. (1).

#### Theorem 7.2

The Fock-Goncharov co-ordinates $X_i(\hbar )$ satisfy

$$\begin{aligned} \exp (z_i/\hbar )\cdot X_i(\hbar ) \rightarrow \xi _i, \end{aligned}$$

as $\hbar \rightarrow 0$ in any closed subsector of the half-plane ${\text {Re}}(\hbar )>0$.

We defer the proof of this result to the Appendix (written by Davide Masoero).

### Behaviour as $\hbar \rightarrow \infty$

The final step is to check the condition (RH3). In fact we will prove more, namely that, for a fixed point $m\in M$, the point $F(\hbar )(m)$ of the monodromy manifold tends to a well-defined limit point. To see this, we will use the homogeneity of the potential (2) under the $\mathbb {C}^*$ action of Remark 3.5(b).

#### Proposition 7.3

For any point $m\in M$ the monodromy $F(\hbar )(m)\in V$ has a well-defined limit as $\hbar \rightarrow \infty$ in a fixed half-plane. This limit is independent of $m\in M$ and is one of the two fixed points of the $\mathbb {Z}/5\mathbb {Z}$ action of Remark 2.2.

#### Proof

Let us consider the partial compactification

$$\begin{aligned} {\bar{M}}=\big \{(a,b,q,p,r)\in \mathbb {C}^5: p^2=q^3+aq+b\big \} \end{aligned}$$

of the space $M$, obtained by dropping the vanishing discriminant condition. We denote by $0\in {\bar{M}}$ the point where all co-ordinates vanish. For a given $\hbar \in \mathbb {C}^*$ the monodromy map $F(\hbar )$ extends to a holomorphic map

$$\begin{aligned} {\bar{F}}(\hbar ):{\bar{M}}\rightarrow V, \end{aligned}$$

subject to the usual warning that this depends on a choice of fifth root of $\hbar ^2$.

Consider the action of $\mathbb {C}^*$ on ${\bar{M}}$ of Remark 3.5(b) which scales the co-ordinates (*a*, *b*, *q*, *p*, *r*) with weights (4, 6, 2, 3, 1) respectively. Note that if we also rescale $\hbar$ with weight 5, and *x* with weight 2, then the Eq. (1) is unchanged. Thus for all points $(a,b,q,p,r)\in {\bar{M}}$

$$\begin{aligned} {\bar{F}}(\hbar )(a,b,q,p,r)={\bar{F}}(\uplambda ^{5}\hbar )(\uplambda ^{4}a,\uplambda ^{6}b,\uplambda ^{2}q,\uplambda ^{3}p,\uplambda ^1 r). \end{aligned}$$

Taking $\uplambda ^{5}\cdot \hbar =1$, and sending $\hbar \rightarrow \infty$ in a fixed half-plane, it follows that the monodromy of $F(\hbar )(m)$ tends to the finite limit ${\bar{F}}(1)(0)$, which is the monodromy of the equation

$$\begin{aligned} y''(x)=\bigg ({x^3}+ \frac{3}{4x^2}\bigg ) y(x). \end{aligned}$$

For the final claim, note that the above $\mathbb {C}^*$ action induces an action of the fifth roots of unity $\mu _5\subset \mathbb {C}^*$, which leaves $\hbar$ invariant. Since this action rescales *x* by an element of $\mu _5$, the monodromy map $F(\hbar )$ intertwines this action with the $\mathbb {Z}/5\mathbb {Z}$ action on *V* of Remark 2.2. But the special point $0\in {\bar{M}}$ is clearly fixed by the $\mu _5$ action, so its image is also a fixed point. $\square$

## Figures and Tables

**Fig. 6:**
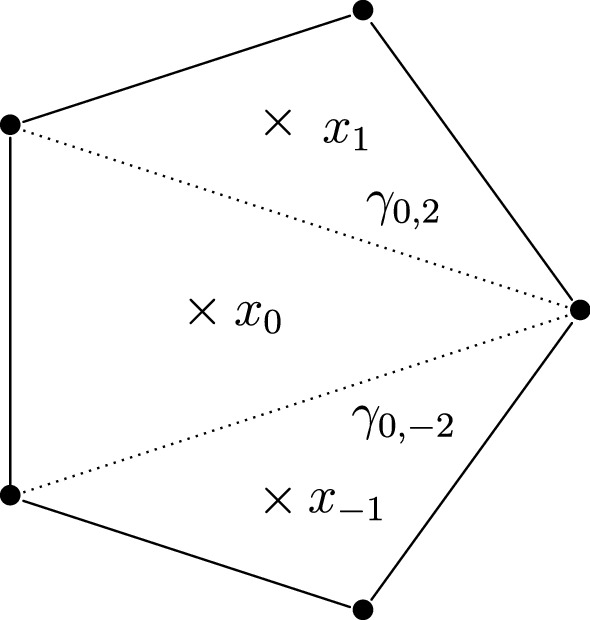
Schematic representation of the trajectories $\gamma _{0,\pm 2}$ and of the roots of $Q_0(x)$

**Fig. 7:**
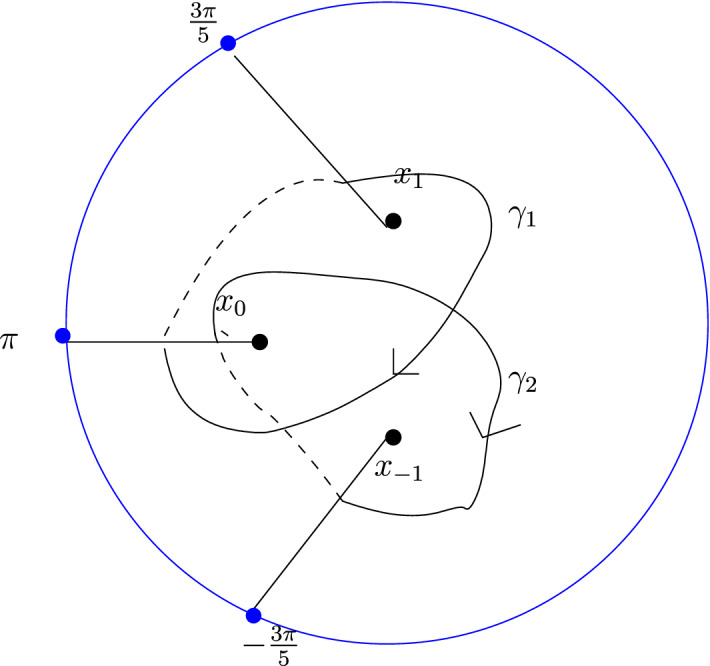
The elliptic curve $X_s$ as a double-sheeted cover, with the cycles $\gamma _{1,2}$ defined by the WKB triangulation

**Fig. 8:**
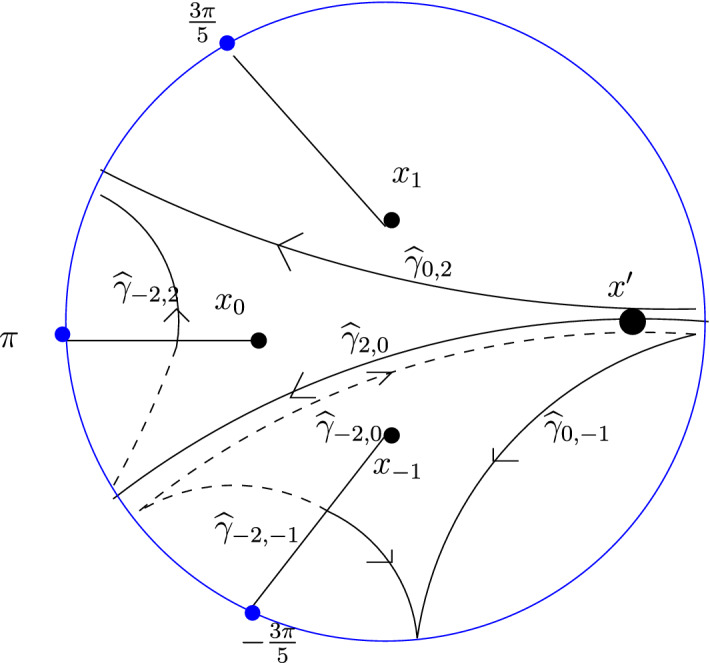
The *Bacalhau (cod) diagram*. Integration paths for the approximate functions $Y_0,Y_{\pm 2}$

**Fig. 9:**
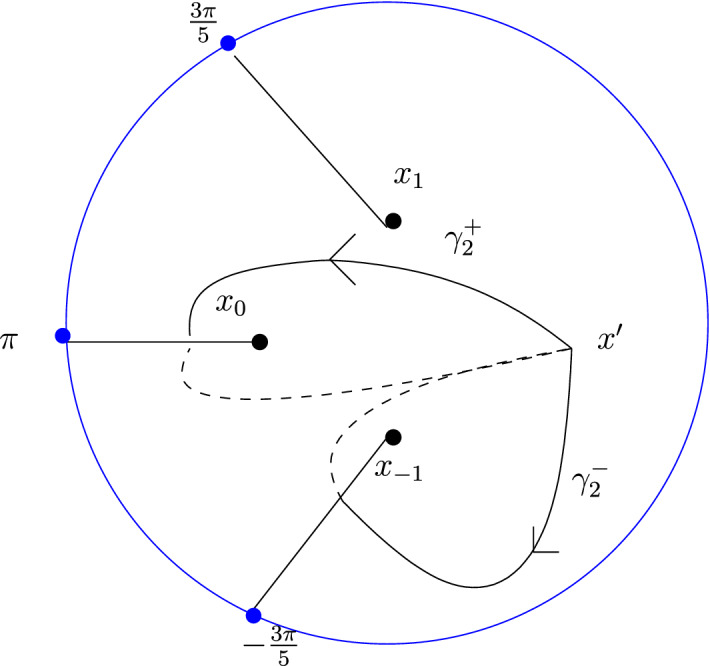
The paths used in formulas (130,131)

## Appendix A: Asymptotic analysis of the functions $X_i(\hbar )$ by Davide Masoero

The Appendix is dedicated to the computation of the full asymptotic expansion of the function $e^{\frac{z_i}{\hbar }} X_i(\hbar ), i=1,2$. As a particular case, we prove Theorem 7.1 of the main text.

The Appendix is organised as follows. In Sect. A.2 we study the asymptotic expansion of solutions of the deformed cubic oscillator according to the Complex WKB method. In Sect. A.5 we lift the formal WKB solutions to the elliptic curve $X_s$ punctured at the branch points. Finally, in Sect. A.6 we prove Theorem A.1. We made this Appendix self-contained. The proofs are lengthy but complete, and the reader experienced in the complex WKB method may want to skip all proofs until the last section.

### Statement of the result

In order to state the our main result, we begin by fixing some notation. Recall that we deal with the small $\hbar$ limit of the deformed cubic oscillator, $y''(x)=Q(x)$, $Q(x)=\hbar ^{-2}Q_0(x)+\hbar ^{-1}Q_1(x)+Q_0(x)$, where

98 $$\begin{aligned} Q_0=x^3+ax+b, \; Q_1(x)=\frac{p}{x-q}+r, \; Q_2(x)=\frac{3}{4(x-q)^2}+\frac{r}{2p(x-q)}+\frac{r^2}{4p^2}.\nonumber \\ \end{aligned}$$

Here *r* is an arbitrary complex number, while the parameters (*q*, *p*) are assumed to belong to the affine elliptic curve $X_s^\circ =\lbrace p^2=Q_0(q) \rbrace$, punctured at the three branch points $p=0$. We call *B* the set of the three branch points. The projectivization of the affine elliptic curve is called $X_s$, and it is endowed with the canonical double cover $p: X_s \rightarrow {\mathbb {P}}^1$, which is branched at *B* and at infinity.

The asymptotic expansion of the Fock-Goncharov co-ordinates is naturally written in terms of complete elliptic integrals over $X_s$. The following meromorphic abelian differentials $\alpha (x)\,dx$ on $X_s$ are relevant to our analysis

99 $$\begin{aligned} \alpha _0(x)= & {} \sqrt{Q_0(x)},\quad \alpha _1(x)= -\frac{Q_0'(x)}{4 Q_0(x)} + \widetilde{\alpha _1}, \text{ with } \widetilde{\alpha _1}=\frac{ Q_1(x)}{2\sqrt{Q_0(x)}}\nonumber \\ \alpha _2(x)= & {} \frac{1}{ 2 \sqrt{Q_0(x)}} \big (Q_2(x)-\alpha _1'(x)-\alpha _1^2(x) \big ) \nonumber \\ \alpha _k(x)= & {} -\frac{1}{ 2 \sqrt{Q_0(x)}} \big (\alpha _{k-1}'(x)+ \sum _{j=1}^{m-1} \alpha _j(x) \alpha _{k-j}(x) \big ) , \; k\ge 3 . \end{aligned}$$

The cycles along which the above differentials are evaluated are the cycles $\gamma _{i}, i=1,2$ defined in the main text.^5^

Finally, we use the following formalism in dealing with asymptotic expansions in sectors of the complex $\hbar$ plane. For every $\theta \in [0,\frac{\pi }{2}[$ and $\hbar _\theta >0$, we let $S_{\theta ,\hbar _\theta }$ denote the sector $\lbrace |\arg \hbar |\le \theta ,0< |\hbar |\le \hbar _\theta \rbrace$. For any formal power series $A =\sum _{k\ge 0} a_k \hbar ^k \in {\mathbb {C}}[[\hbar ]]$, we denote by $A_m:=\sum _{k=0}^m a_k \hbar ^k$ its $m-th$ truncation.

#### Definition A.1

Let *f* be a function on the sector ${\text {Re}}\hbar >0$, and *A* a formal power series. We say that *f* is asymptotic to *A*, and we write $f \approx A$ on ${\text {Re}}\hbar >0$, if for every $\theta \in [0,\frac{\pi }{2}[$ there exists a sequence of positive constants $\hbar _{\theta }$, $C_{\theta ,m}, m\ge 0$ such that $|f(\hbar )-A_m(\hbar )|\le C_{\theta ,m} |\hbar |^{m+1}$ for all $\hbar \in S_{\theta ,\hbar _\theta }$.

#### Theorem A.1

Assume that $Q_0$ is saddle-free. The functions $e^{\frac{z_i}{\hbar }} X_i(\hbar ), i=1,2$ have the following asymptotic expansion

100 $$\begin{aligned} e^{\frac{z_i}{\hbar }} X_i(\hbar ) \approx \xi _i \exp {\left( \sum _{k=1}^{\infty } \hbar ^{k} C_{k,i} \right) } \text{ on } {\text {Re}}\hbar >0, \end{aligned}$$

where

101 $$\begin{aligned} z_i=\int _{\gamma _i}\alpha _0(x) dx, \quad \xi _i= e^{-\int _{\gamma _i}\big ({\widetilde{\alpha }}_1(x)+\frac{1}{2(x-q)} \big )dx}, \quad C_{k,i}= - \int _{\gamma _i} \alpha _{k+1}(x) \, dx , \, k\ge 1.\nonumber \\ \end{aligned}$$

In particular,

$$\begin{aligned} \lim _{\hbar \rightarrow 0} e^{ \frac{z_i}{\hbar }} X_i(\hbar ) = \xi _i \text{ in } \text{ any } \text{ closed } \text{ subsector } \text{ of } {\text {Re}}\hbar >0 . \end{aligned}$$

#### Remark A.2

Before we tackle the proof of the Theorem, we check that all terms in the asymptotic expansions (101) attain the same value for every path $\gamma$ in the homology class $\gamma _i \in H_1(X_s^\circ \setminus B, {\mathbb {Z}})^- ,i=1,2$, even though the forms $\alpha _k$ are possibly singular at the points $(q,\pm p)$.

This is indeed the case. In fact,

$(k=0)$: The only singular point of $\alpha _0(x) dx$ is $\infty$. Furthermore its residue is zero.
$(k=1)$: $e^{-\int _{\gamma _i}\big ({\widetilde{\alpha }}_1(x)+\frac{1}{2(x-q)} \big )dx}$ is well-defined, since all residues of the form $\big ({\widetilde{\alpha }}_1(x)+\frac{1}{2(x-q)} \big )dx$ are integer-valued, as it is shown in the main text after formula (42).
$(k\ge 2)$: For the forms $\alpha _k(x)dx, k \ge 2$, the residue at $(q,\pm p)$ vanishes, see Corollary A.12 below.

### WKB analysis of the deformed cubic oscillator

Our approach is based on transforming a linear ODE of the second order into an integral equation of Volterra type, following [13]: We consider a second order scalar linear ODE of the form

102 $$\begin{aligned} y''(x)=Q(x) y(x), \quad x \in {\mathbb {C}} \end{aligned}$$

where *Q*(*x*) may depend on additional parameters, and a putative approximate solution *Y*(*x*), which we suppose to be of such a form that

103 $$\begin{aligned} u(x)=\frac{y(x)}{Y(x)} \end{aligned}$$

is well-defined and approximately 1 in a certain domain of ${\mathbb {C}}$ to be later specified.

Defining the forcing term

104 $$\begin{aligned} F(x)=Q(x)-\frac{Y''(x)}{Y(x)} \end{aligned}$$

the Eq. (102) for *y*(*x*), when rewritten in terms of the function *u*(*x*) defined by (103), becomes

105 $$\begin{aligned} \frac{d}{dx}\big ( Y^2(x) u'(x) \big )- Y^2(x) F(x) u(x)=0 \end{aligned}$$

We fix a point $x'$ in the Riemann sphere, the boundary conditions $u'(x')=0,u(x')=1$, and a piece-wise smooth integration path $\gamma$ connecting $x'$ to another point $x \in {\mathbb {C}}$. Integrating twice Eq. (105), *u*(*x*) is proven to solve the following integral equation

106 $$\begin{aligned} u(x)=1-\int _{x',\gamma }^x K(x,s) F(s) u(s) d s \,, \qquad K(x,s)=\int _{s,\gamma }^x \frac{Y^2(s)}{Y^2(r)} d r \; , \end{aligned}$$

provided the above integral converges absolutely.

Conversely, given any continuous solution *u*(*x*) of the latter integral equation, the function $y(x):=u(x) Y(x)$ solves (102) and satisfies the (possibly singular) Cauchy problem

$$\begin{aligned} \lim _{x \rightarrow x', x \in \gamma } \frac{y(x)}{Y(x)}=1 , \quad \lim _{x \rightarrow x', x \in \gamma } \frac{y'(x)}{Y'(x)}=1 \end{aligned}$$

#### Remark A.3

Given a solution *u* of (106), the corresponding solution *y* of (102) is a priori only defined on the trajectory of the curve $\gamma$. It can however be analytically extended to any open simply connected domain of analyticity of *Q* which intersects the trajectory of $\gamma$. To be more precise: let $D \subset {\mathbb {C}}$ be an open simply connected domain such that $Q_{|D}$ is analytic, and assume that, for some $t_1<t_2$, $\gamma (t) \in D$ for all $t\in ]t_1,t_2[$; there exists a unique solution ${\widehat{y}}:D \rightarrow {\mathbb {C}}$ such that ${\widehat{y}}(\gamma (t))=u(\gamma (t))$ for all $t \in ]t_1,t_2[$.

### Formal WKB solutions

We are interested in studying the small $\hbar$ limit of the equation $y''(x)=Q(x)$ where $Q(x)=\hbar ^{-2}Q_0(x)+\hbar ^{-1}Q_1(x)+Q_0(x)$, as per (98).

The $m-th$ WKB approximation, with $m\ge 0$, is provided by the function

107 $$\begin{aligned} Y_m(x;x')= \exp \left\{ \hbar ^{-1} \sum _{k=0}^{m+1} \int _{x'}^x \hbar ^k \alpha _k(s) ds \right\} , \end{aligned}$$

where the forms $\alpha _k(x) dx$ are recursively determined by the following requirement on the forcing term

108 $$\begin{aligned} F_m:=Q(x)- \frac{Y_m''(x)}{Y_m(x)}=O(\hbar ^m). \end{aligned}$$

A simple computation shows that the forms $\alpha _k$ are given by Eq. (99), and that

109 $$\begin{aligned} F_0(x)= & {} \alpha _1^2(x)+\alpha _1'(x)-Q_2 , \nonumber \\ F_m(x)= & {} \hbar ^m \left( \alpha '_{m+1}(x)+ \sum _{k=m}^{2m} \hbar ^{k-m } \sum _{l=0}^{2m-k} \alpha _{m+1-l}\alpha _{k+1-m+l} \right) . \end{aligned}$$

The following Lemma will be useful in the proof of the main result.

#### Lemma A.4

The forms $\widetilde{\alpha _1}dx$, $\alpha _k(x) dx, k\ge 2$ and ${\widehat{F}}_m:=\frac{F_{m}(x)}{\sqrt{Q(x)}}dx, m\ge 0$ are holomorphic on $X_s\setminus V$ where $V=B\cup \lbrace (q,p),(q,-p) \rbrace$.

#### Proof

The forms under consideration are well-defined and meromorphic on $X_s$ since they are represented by the formula $\beta (x) dx$ with $\beta (x)=R(x,\sqrt{Q_0(x)})$ for some rational function *R*. Because of formula (99), they are manifestly holomorphic on $X_s$ punctured at *V* and at $\infty$. Hence the thesis is proven if they are shown to be holomorphic at $\infty$.

Recall that a meromorphic form on $X_s$, written as $\beta (x) dx$, is regular at $\infty$ if the degree of $\beta (x)$ at $\infty$ is less or equal than $-\frac{3}{2}$ (in fact a good local parameter at $\infty$ is $\tau =x^{-\frac{1}{2}}$ so that $dx=-2 x^{\frac{3}{2}} d \tau$). Let $d_k$ denote the degree of $\alpha _k(x)$ at $\infty$. After formula (99) we have that $d_1=-1$, $\deg {\widetilde{\alpha }}_1=-\frac{3}{2}$, $d_2=-\frac{3}{2}$; moreover, we recursively obtain $d_{2k+1}=-1-3(2k-1)$, and $d_{2k+2}=d_{2k+1}-\frac{1}{2}$. Let ${\widehat{d}}_m$ denote the degree of $\frac{F_{m}(x)}{\sqrt{Q(x)}}$ at $\infty$. After formula (109), we have that ${\widehat{d}}_0=-\frac{3}{2}$ and, recursively, ${\hat{d}}_k=d_{k+2}$. The thesis is proven. $\square$

### WKB estimates

Our method of analysis of the deformed cubic oscillator is based on the study of the integral Eq. (106), in the case the approximate solution is the formal WKB solution $Y_m(x;x_0)$ and the forcing term is $F_m(x)$, as defined by formulas (107) and (109).

In order to cosntruct a solution of (102) using the integral Eq. (106), we first need to choose an integration path $\gamma$ in such a way that the integral equation admits a solution *u* which converges uniformly to 1, as $\hbar \rightarrow 0$, in all sectors of the form $\hbar \in S_{\theta ,\hbar _{\theta }}$ with $\theta \in [0,\frac{\pi }{2}[$.

The complex WKB method provides such a solution whenever the integration path $\gamma :[0,1] \rightarrow {\mathbb {P}}^1$ satisfies the following inequality in the sector ${\text {Re}}\hbar >0$

110 $$\begin{aligned} \left| \arg \hbar ^{-1}\int _{t'}^{t} \sqrt{Q_0(\gamma (s))}{\dot{\gamma }}(s)ds \right|<\frac{\pi }{2}, \quad \forall t,t'\in ]0,1[ \text{ such } \text{ that } 0<t'<t<1 ,\nonumber \\ \end{aligned}$$

for one of the two choices of $\sqrt{Q_0}$.

It is straightforward to see that the only paths which satisfy inequality (110) are the horizontal trajectory of $Q_0 dx^{\otimes 2}$, since these are the steepest descent paths for the function ${\text {Re}}w$, where $w(x):=\int ^x \sqrt{Q_0(s)} ds$ (equivalently the arcs along which ${\text {Im}}w$ is constant). More precisely, the horizontal trajectories that serve our purposes are those horizontal trajectories that can be prolonged indefinitely without crossing any zero of $Q_0$. These admit a maximal extension to a simple closed Jordan curve $\gamma :=\gamma _{k,k'}:[0,1] \rightarrow {\mathbb {P}}^1$, that satisfies the following three Properties

1. As $t \rightarrow 0$, $\gamma (t)$ is asymptotic to the ray of argument $\frac{2 \pi k}{5}$, for some $k \in {\mathbb {Z}}$.
2. As $t \rightarrow 1$, $\gamma (t)$ is asymptotic to the ray of argument $\frac{2 \pi k'}{5}$, for some $k' \in {\mathbb {Z}}$, $k \not \equiv k' \mod 5$.
3. $\frac{|{\dot{\gamma }}_{k,k'}(t)|}{|\gamma _{k,k'}(t)|}\rightarrow 1$ as $t\rightarrow 0$ or $t \rightarrow 1$, which implies that $\int _0^1 | f(\gamma _{k,k'}(t)){\dot{\gamma }}_{k,k'}(t)| dt$ converges for every function *f* continuous on the support of $\gamma _{k,k'}$, which decays at $\infty$ as $x^{-1-\varepsilon }$ for some $\varepsilon >0$.

Properties (1,2) above were recalled in Section 6.1 of the main text. Property (3) follows rather directly from the expansion $w = \frac{2}{5} x^{\frac{5}{2}}+O(x^{\frac{1}{2}})$; see [30, §7.3] for a proper proof.

We notice that the set of horizontal trajectories is naturally partitioned into subsets of trajectories with the same end points. Hence the following definition is quite natural.

#### Definition A.5

For every $k,k' \in {\mathbb {Z}}$, we denote by $\Gamma _{k,k'}$ the set of oriented horizontal trajectories leaving $\infty$ parallel to the ray of argument $\frac{2\pi k}{5}$ and arriving at $\infty$ parallel to the ray of argument $\frac{2\pi k'}{5}$. Every element of $\Gamma _{k,k'}$ is endowed with a parametrisation $\gamma :[0,1] \rightarrow {\mathbb {P}}^1$, satisfying the properties P(1,2,3) listed above. We denote by the same symbol $\gamma$ an oriented trajectory and its parametrisation.

#### Remarks A.6

(i) For every $k,k'$, the set $\Gamma _{k,k'}\cong \Gamma _{k',k}$ is either empty or diffeomorphic to the real line. The set ${\mathbb {C}} \setminus \cup _{k\ne k'}\lbrace x \in \gamma , \gamma \in \Gamma _{k,k'} \rbrace$ is known as the anti-Stokes complex. It is the union of the roots of $Q_0$ with the horizontal trajectories emanating from them.
(ii) Given a point $q \in {\mathbb {C}}\setminus \lbrace Q_0(x)=0 \rbrace$, it belongs to at most one curve $\gamma \in \Gamma _{k,k'}$. If *q* belongs to a curve $\gamma _q \in \Gamma _{k,k'}$, then this curve separates $\Gamma _{k,k'}\setminus \gamma _q$ into two non-empty disjoint subsets. A curve belonging to one subset is homotopic in ${\mathbb {P}}^1 \setminus \lbrace q \rbrace$ to any other curve in the same subset, and not-homotopic to any curve belonging to the complementary subset.
(iii) If $k'=k \pm 1$, the set $\Gamma _{k,k'}$ is not empty and moreover
  $$\begin{aligned} \inf _{\gamma \in \Gamma _{k,k\pm 1}} \int _0^1 | f(\gamma (t)){\dot{\gamma }}(t)| dt =0 \end{aligned}$$
  for every function *f* that is defined on a neighbourhood of $\infty$, which decays as $x^{-1-\varepsilon }$ for some $\varepsilon >0$.
(iv) The condition $Q_0(x) dx^{\otimes 2}$ is saddle free can be rephrased as follows: there exists a *k* such that both $\Gamma _{k,k+2}$ and $\Gamma _{k,k-2}$ are not empty.

In order to prove our main result, we need to relax inequality (110) to allow for slightly more general integration curves.

#### Definition A.7

For every $k,k' \in {\mathbb {Z}}$ and any $\theta \in [0,\frac{\pi }{2}[$, we denote by $\Gamma ^{\theta }_{k,k'}$ the set of curves $\gamma :[0,1] \rightarrow {\mathbb {P}}^1$, satisfying the properties (P1,P2,P3) of the horizontal trajectories, and moreover such that there exists an $\varepsilon _{\gamma }>0$ such that

111 $$\begin{aligned} \left| \arg \int _{t'}^{t} \sqrt{Q_0(\gamma (s))}{\dot{\gamma }}(s) ds \right| \le \frac{\pi }{2}-\theta -\varepsilon _{\gamma }, \qquad \forall t'<t \end{aligned}$$

for one of the two choices of $\sqrt{Q_0(x)}$.

The great advantage of relaxing (110)–(111) is that we are able to deform the integration paths. More precisely, we have the following Lemma.

#### Lemma A.8

Suppose that $\Gamma _{k,k'}$ is not-empty. For any $\gamma \in \Gamma _{k,k'}$ such that $q \notin \gamma$, and any $\theta \in [0,\frac{\pi }{2}[$, there exists a $\gamma _{\theta } \in \Gamma _{k,k'}^{\theta }$ satisfying the following properties:

- $\gamma _{\theta }$ is homotopic to $\gamma$ in ${\mathbb {P}}^1\setminus \lbrace \lbrace Q_0(x)=0 \rbrace \cup \lbrace q \rbrace \rbrace$;
- there exist $0<t_1<t_2<1$ such that $\arg \gamma _{\theta }(t)=\frac{2\pi k}{5}$ for all $t\in ]0,t_1]$ and $\arg \gamma _{\theta }(t)=\frac{2\pi k'}{5}$ for all $t\in [t_2,1[$.

#### Proof

The easy proof is left to the reader. $\square$

We have introduced the integration curves which we will use to define the integral Eq. (106) and to prove Theorem A.1. Before dealing with the analysis of (106), we need a last preparatory lemma.

#### Lemma A.9

Let $\beta (x)dx$ be one of the forms considered in Lemma A.4: namely $\beta$ is either ${\widetilde{\alpha }}_1$, or $\alpha _k$ $k\ge 2$, or ${\widehat{F}}_k$, $k\ge 0$. Then

$$\begin{aligned} \int _0^1 |\beta (\gamma (t)) {\dot{\beta }}(t)| dt < \infty , \quad \forall \gamma \in \Gamma ^{\theta }_{k,k'} \text{ such } \text{ that } q \notin \gamma . \end{aligned}$$

#### Proof

It follows from Lemma A.4 and Property (3) of the paths $\Gamma ^{\theta }_{k,k'}$. $\square$

We now prove the fundamental estimate underlying the complex WKB method.

#### Proposition A.10

Fix a $\theta \in [0,\frac{\pi }{2}[$, a $\gamma \in \Gamma ^{\theta }_{k,k'}$ such that $q \notin \gamma$, a $t_0 \in ]0,1[$ and the branch of $\sqrt{Q_0(x)}$ in such a way that $\lim _{t\rightarrow 0}{\text {Re}}\int _{t}^{t_0} \sqrt{Q_0(\gamma (t))}{\dot{\gamma }}(t) dt =\infty$.

For any $\hbar _{\theta }>0$, there is a sequence of positive constants $C_{m}, m\ge 0$ - depending on $\theta , \gamma$ - and a unique sequence of solutions $y_{k,m}(x)$ of the deformed cubic oscillator satisfying the following inequality

112 $$\begin{aligned} \sup _{t \in [0,1]} \left| \frac{y_{k,m}(\gamma (t))}{Y_m(\gamma (t),\gamma (t_0))}-1 \right| \le C_{m} |\hbar |^{m+1} , \quad \forall \hbar \in S_{\theta ,\hbar _\theta } \end{aligned}$$

where $Y_m(x;\gamma (t_0))$ is the formal WKB solution defined by formula (107) with $x=\gamma (t),x'=\gamma (t_0)$.

Moreover, the solutions $y_{k,m}$ satisfy the following properties

1. $\lim _{|x| \rightarrow \infty }y_{k,m}(e^{i\frac{2\pi k}{5}}|x|)=0$. Equivalently, $y_{k,m}(x)$ is subdominant in the k-th Stokes sector.
2. $$y_{k,m}(x)=D_m \, y_{k,0}(x)$$, with $$D_m=\exp \big (- \sum _{k=2}^{m+1}\hbar ^k \int _{0}^{t_0} \alpha _k(\gamma (t)) \dot{\gamma (t)} dt \big )$$.
3. $\lim _{|x| \rightarrow \infty }|y_{k,m}(e^{i\frac{2\pi k'}{5}}|x|)|=\infty$. Equivalently, $y_{k,m}(x)$ is dominant in the k’-th Stokes Sector.

#### Proof

We introduce an order relation on $\gamma$: $v\le x$ if $v=\gamma (s), x=\gamma (t)$ and $s\le t$. We use the following convention: $\int _{\gamma (s),\gamma }^{\gamma (t)} f(v) dv:= \int _{s}^t f(\gamma (t')) {\dot{\gamma }}(t') dt'$, and $\int _{\gamma (s),\gamma }^{\gamma (t)}| f(v) dv|:= \int _{s}^t |f(\gamma (t')) {\dot{\gamma }}(t')| dt' .$

According to the general theory we provide the solution $y_{k,m}$ (hence we prove its existence) by analysing the integral Eq. (106) for the ratio $u(x):=\frac{y_{k,m}(x)}{Y_m(x)}$. For convenience we rewrite the integral equation in the following form

113 $$\begin{aligned} u(x)=1- \hbar \int _{\infty ,\gamma }^x{\widehat{K}}_m(x,v) {\widehat{F}}_m(v) u(v) dv , \end{aligned}$$

where ${\widehat{K}}_m(x,v)=\hbar ^{-1}\sqrt{Q_0(v)} \int _{v,\gamma }^x \frac{ Y_m^2(v)}{Y_m^{2}(r)} dr$, and ${\widehat{F}}_m(v)=\frac{F_m(v)}{\sqrt{Q_0(v)}}$ with $F_m(x)$ as defined in (109).

We divide the analysis of the integral equation (113) in two steps

1. We show the estimate: For any given $\theta <\frac{\pi }{2}$, if $|\hbar |$ is smaller than an arbitrary, but fixed, constant $\hbar _{\theta } >0$, there exists a $C_m>0$ such that $|{\widehat{K}}_m(x,v)|\le C_m$ for all $x,v \in \gamma , x\le v$.
2. We use the above estimate on $|{\widehat{K}}_m(x,v)|$ to study the integral equation and prove the thesis.

Step 1. In order to estimate ${\widehat{K}}_m(x,v)$ we need to control the integral $\int _{v,\gamma }^x \frac{ Y_m^2(v)}{Y_m^{2}(r)} dr$, where $Y_m$ is the formal WKB solution. If $m\ge 1$, the integral $\int _{v,\gamma }^x \frac{ Y_m^2(v)}{Y_m^{2}(r)} dr$ cannot be computed in close form. To overcome this difficulty we factorise $Y_m(x)$ as $Y(x)T_m(x)$, where *Y* is an unbounded function such that $\int Y^{-2}(r) dr$ can be computed in closed form, and $T_m$ is a bounded function (with bounded derivatives).

Explicitly, we make the following choice

$$\begin{aligned} Y(x)= & {} \exp {\big ( \int _{x',\gamma }^x \hbar ^{-1} \sqrt{Q_0(w)} -\frac{Q_0'(w)}{4Q_0(w)}dw \big )} , \\ T_m(x)= & {} \exp {\big ( \int _{x',\gamma }^x {\widetilde{\alpha }}_1(w) + \sum _{l=1}^{m+1}\hbar ^l \alpha _l(w) dw \big )}, \end{aligned}$$

where $x'=\gamma (t_0)$, and the forms $\alpha$ are as in (99).

We notice that $Y^{-2}(r)= \frac{\hbar }{2}\frac{d}{dr} e^{-\frac{2}{\hbar }\int _{x'}^r \sqrt{Q_0(w)} dw}$ and we integrate by parts to obtain

$$\begin{aligned} {\widehat{K}}_m(x,v)= \frac{1}{2} \left( e^{-\frac{2}{\hbar }\int _v^x \sqrt{Q_0(w)} dw}\frac{T^{2}_m(v)}{T^2_m(x)}-1 \right) - \frac{1}{2} \int _v^x e^{-\frac{2}{\hbar }\int _v^r Q(w) dw} \frac{d}{d r} \frac{T_m^2(v)}{T_m^{2}(r)} dr \end{aligned}$$

Due to Lemma A.4, the functions $T_m(x), T_m^{-1}(x)$ as well as all their derivatives are uniformly bounded on $\gamma$, provided $|\hbar |$ is bounded. It follows that

114 $$\begin{aligned} |{\widehat{K}}_m(x,v)|\le C_{m,1} \left( 1+ |e^{-\frac{2}{\hbar }\int _v^x \sqrt{Q_0(r)} dr}| + \int _{v,\gamma }^x |e^{-\frac{2}{\hbar }\int _{v,\gamma }^r \sqrt{ Q_0(r')} dr'} dr| \right) ,\nonumber \\ \end{aligned}$$

where $C_{m,1}$ is a sufficiently high positive constant (in the third term we have used the Hölder inequality).

By definition of $\Gamma _{k,k'}^{\theta }$ there exists an $\varepsilon _{\gamma }>0$ such that

115 $$\begin{aligned} |\arg \int _{v}^{x} \sqrt{Q_0(\gamma (s)})ds |\le \frac{\pi }{2}-\theta -\varepsilon _{\gamma }, \quad \forall v,x \in \gamma (]0,1[) \text{ such } \text{ that } v\le x .\nonumber \\ \end{aligned}$$

From the above inequality it follows directly that $|e^{-\frac{2}{\hbar }\int _v^x \sqrt{Q(r)} dr}|\le 1$ for all $v,x \in \gamma (]0,1[)$ such that $v\le x$.

We complete Step 1 by showing that the inequality (115) implies that also the third term in (114) is uniformly bounded by a constant $C_2$. To be more precise we show that there exists a $C_{2}<\infty$ such that

116 $$\begin{aligned} E(x,v):= \int _{v}^x |e^{-\frac{2}{\hbar }\int _{v}^{r} \sqrt{Q(r')} dr'} dr| \le C_{2} \text{ if } v\le x. \end{aligned}$$

We notice that, due to (115), under the function $w(x)=\int _{x'}^x \sqrt{Q_0(r)}dr$, the curve $\gamma$ is mapped onto a curve which is diffeomorphic to its projection on to the real axis. We call *g* such a curve and we parametrise it by its real part; explicitly, with $x={\text {Re}}w(\gamma (t))$, ${\text {Re}}g(x)=x$, ${\text {Im}}g(x)={\text {Im}}w(\gamma (t))$. Using $x={\text {Re}}(w(\gamma (t))$ as the new variable of integration in (116), we transform the problem of bounding *E*(*x*, *v*) into the equivalent problem: prove that there exists a $C_2'>0$ such that

117 $$\begin{aligned} {\widetilde{E}}(x,y):= \int _{y}^x \big |e^{-\frac{2}{\hbar }\big ( g(x')-g(y)\big )} \frac{\frac{d g(x')}{d x'}}{\sqrt{Q_0\big (\Phi (x')\big )}}\big | dx' \le C_2' , \qquad \forall y\le x \in {\mathbb {R}},\nonumber \\ \end{aligned}$$

where $\Phi$ is the inverse of *w* composed with *g*. The functions $\big |Q_0^{-\frac{1}{2}}\big (\Phi (x)\big )\big |$ and $|\frac{d g(x}{d x}|$ are bounded. Indeed, $Q_0^{-\frac{1}{2}}(\Phi ({\text {Re}}(x)))$ decays as $|x| \rightarrow \infty$ (one can show as $O(|x|^{-\frac{2}{5}})$); moreover $|\frac{d g(x)}{dx}|$ converges to 1 as $|x| \rightarrow \infty$ by definition of $\Gamma _{k,k'}^{\theta }$ (Property (P3) of the steepest descent paths). Hence if we show that $\int _{y}^x \big |e^{-\frac{2}{\hbar }\big ( g(x')-g(y)\big )}\big |dx'$ is smaller than a constant $C_2''$ for all $y\le x$, (117) follows by the Hölder inequality. To this aim, we notice that (115) implies that $\big |e^{-\frac{2}{\hbar }\big ( g(x')-g(y)\big )}\big |\le e^{-\frac{2 \cos {(\frac{\pi }{2}-\varepsilon _{\gamma })}}{|\hbar |}(x'-y)}$ for all $y\le x'$; integrating the right hand side we obtain that

$$\begin{aligned} \int _y^x \big |e^{-\frac{2}{\hbar }\big ( g(x')-g(y)\big )}\big | dx \le \frac{|\hbar _{\theta }|}{2 \sin (\varepsilon _{\gamma }) }, \qquad \forall y\le x , \end{aligned}$$

which completes the proof of Step 1.

Step 2. We denote by ${\mathcal {C}}_{\gamma }$ the space of continuous functions supported on $\gamma$ endowed with the supremum norm $\Vert f\Vert _{\infty }$. In this space we define the linear operator $K_m$ by the formula

$$\begin{aligned} K_m[f](x)=-\hbar \int _{\infty ,\gamma }^x {\widehat{K}}_m(x,v) {\widehat{F}}_m(v) f(v) dv \end{aligned}$$

which allows us to write the integral Eq. (113) in the compact form $u=1+ K_m[u]$. As we will show, this integral equation admits a (unique) continuous solution, which is of the form $u=\sum _{N=0}^{\infty }K_m^N [1]$, where 1 is the constant function 1 on $\gamma$ and $K_m^N$ is the *N*-th iterate of *K*.

After Step 1., for every $0<\theta <\frac{\pi }{2}$ there exist a sequence of positive constants $\hbar _{\theta },{\widehat{C}}_m,m\ge 0$ such that $|{\widehat{K}}_m(x,s)|\le {\widehat{C}}_m<\infty$ for all $\hbar \in S_{\theta ,\hbar _{\theta }}$. Furthermore, due to Lemma A.9, there exists another sequence of positive constants $\rho _m,m\ge 0$ such that $\int _{0}^1 |{\widehat{F}}_m(v) d v | \le |\hbar |^{m} \rho _{\gamma }$ for all $\hbar \in S_{\theta ,\hbar _{\theta }}$. Using once again the Hölder inequality together with the two estimates above, we immediately obtain that the operator $K_m$ is bounded as indeed its operator norm $\Vert K_m\Vert$ is less or equal than $|\hbar |^{m+1}\widehat{C_m} \rho _{m}$. Namely

$$\begin{aligned} \Vert K_m [f]\Vert _{\infty }\le |\hbar |^{m+1} \widehat{C_m} \rho _{m}\Vert f\Vert _{\infty } \text{ for } \text{ every } \text{ bounded } \text{ function } f . \end{aligned}$$

It is a basic fact of integral equations of Volterra type that $\Vert K_m^N\Vert = \frac{\Vert K_m\Vert ^N}{N!}$ (in fact $K_m^N [f]$ is defined as an integral on the *N* dimensional simplex whose volume is $\frac{1}{N!}$; for a detailed proof see e.g. [9, §79]). It follows that the series $u=\sum _{N=0}^{\infty }K_m^N[1]$ converges in ${\mathcal {C}}_{\gamma }$, and that $\Vert u-1\Vert _{\infty }\le e^{|\hbar |^{m+1}\widehat{C_m} \rho _{m}}-1$, for every $\hbar \in S_{\theta ,\hbar _{\theta }}$. Therefore the function $y_{k,m}(x):=u(x) Y_m(x)$ is a solution of the deformed cubic which satisfies the estimate (112).

We complete the proof by proving properties (1,2,3) of $y_{k,m}$, as well as its uniqueness.

1. By construction $\lim _{t \rightarrow 0}Y_m(\gamma (t);\gamma (t_0))=0$ and $\gamma$ is asymptotic to the ray of argument $e^{i\frac{2\pi k}{5}}$ for $t\rightarrow 0$. It follows that $y_{k,m}$ is subdominant in the $k-th$ Stokes sector. It is well-known (see e.g. [27]) that in any given Stokes sector, the subdominant solution is uniquely defined up to a scale. Hence property (1) implies hat $y_{k,m}$ is the unique solution satisfying (112).
2. For the same reason $y_{k,m}=D_m y_{k,0}$ for some $D_m \in {\mathbb {C}}^*$. Since
  $$\begin{aligned} \lim _{t \rightarrow 0}\frac{Y_m(\gamma (t);\gamma (t_0))}{Y_{0}(\gamma (t);\gamma (t_0))}=e^{-\sum _{k=2}^{m+1}\hbar ^k \int _0^{t_0}\alpha _k(\gamma (t)) {\dot{\gamma }}(t) dt}, \end{aligned}$$
  the thesis follows.
3. The thesis follows from the fact that $\lim _{t \rightarrow 1}|Y_m(\gamma (t);\gamma (t_0))|=\infty$.

$$\square $$

#### Remark A.11

One of the hypothesis of the proposition above is that the lower integration point $x'$ in the definition of $Y_m(x;x')$ belongs to the curve $\gamma$. However this condition can be dropped. Choose any other point $x''$ in the complex plane, which is not a root of $Q_0(x)$, and a path $\gamma '$ connecting $x''$ to $x'$. The function ${\hat{y}}_{k,m}=e^{\hbar ^{-1}\sum _{k=0}^{m+1}\int _{x''}^{x'} \alpha _k(x) dx} y_{k,m}(x)$ is a new solution of the deformed cubic oscillator and $\frac{{\hat{y}}_{k,m}(x)}{Y_m(\gamma (t);x'')}$ satisfies the estimate (112), since $\frac{{\hat{y}}_{k,m}(x)}{Y_m(\gamma (t);x'')}= \frac{y_{k,m}(x)}{Y_m(x;x')}$ by construction.

We have the following Corollary.

#### Corollary A.12

Let $(q,p), p \ne 0$ be the point of $X_s$ used to define the potentials $Q_1,Q_2$. We have that

118 $$\begin{aligned} \text{ res}_{(q,\pm p)} \alpha _k(x) dx =0, \; \forall k \ge 2 . \end{aligned}$$

#### Proof

Instead of considering the forms $\alpha _k dx$ as meromorphic differentials on $X_s$, we can consider them as multi-valued meromorphic differentials on ${\mathbb {C}}$. The thesis is then equivalent to $\text{ res}_{x=q} \alpha _k(x) dx =0,\forall k \ge 2$ for both branches of $\sqrt{Q_0(x)}$. We prove this statement here.

We fix a branch of $\sqrt{Q_0}$. We suppose—without loss of generality^6^ - that, there exists a pair $(k,k')$ and a $\gamma _q \in \Gamma _{k,k'}$ such that $q \in \gamma _q$, and $\lim _{t\rightarrow 0}{\text {Re}}\int _{t}^{t_0} \sqrt{Q_0(\gamma _q(t))}\dot{\gamma _q}(t) dt =\infty$.

We can then choose two paths $\gamma ,\gamma '$ in $\Gamma _{k,k'}$, which are not homotopic in ${\mathbb {C}}\setminus \lbrace q \rbrace$; see Remark A.6(ii).

We fix a $\theta \in [0,\frac{\pi }{2}[$. According to Lemma A.8, $\gamma ,\gamma '$ can be deformed to two paths $\gamma _{\theta },\gamma '_{\theta } \in \Gamma _{k,k'}^{\theta }$ such that $\gamma _{\theta }(t)=\gamma '_{\theta }(t)$ as $t\rightarrow 0$, and as $t\rightarrow 1$. These are by construction non-homotopic paths in ${\mathbb {P}}^1 \setminus \lbrace q \rbrace$. Since $\gamma _{\theta },\gamma '_{\theta }$ coincide for large vaue of |*x*|, $\gamma _{\theta }-\gamma '_{\theta }$ defines a non-trivial closed loop in ${\mathbb {C}}\setminus \lbrace q \rbrace$. Let $t_0$ small enough so that $\gamma _{\theta }(t_0)=\gamma '_{\theta }(t_0)$. After Proposition A.10 it follows that there are positive constants $\hbar _{\theta }$ and $C_m, m \ge 0$ such that for all $\hbar \in S_{\theta ,\hbar _{\theta }}$

119 $$\begin{aligned} \sup _{t \in [0,1]}&\left| \frac{y_{k,m}(\gamma _{\theta }(t))}{Y_m(\gamma _{\theta }(t);\gamma _{\theta }(t_0))}-1 \right| \le C_{m} |\hbar |^{m+1} , \quad \sup _{t \in [0,1]}&\left| \frac{y_{k,m}(\gamma _{\theta }'(t))}{Y_m(\gamma '_{\theta }(t);\gamma '_{\theta }(t_0))}-1 \right| \le C_{m} |\hbar |^{m+1}.\nonumber \\ \end{aligned}$$

Here $Y_m$ is the m-th WKB approximation defined in (107). Recall, from the main text, that every non-trivial solution of the deformed cubic is two valued and the point $x=q$ is its branch point. By construction $\gamma _{\theta },\gamma '_{\theta }$ are not homotopic in ${\mathbb {C}}\setminus \lbrace q \rbrace$, hence $\frac{y_{k,m}(\gamma _{\theta }(t))}{y_{k,m}(\gamma '_{\theta }(t))}=-1$ as $t \rightarrow 1$. Moreover we have that

$$\begin{aligned} \frac{Y_{m}(\gamma _{\theta }(t);\gamma _{\theta }(t_0))}{Y_{m}(\gamma '_{\theta }(t);\gamma '_{\theta }(t_0))}= - \exp {\left( 2\pi i \sum _{k=2}^{m+1} \hbar ^{k-1} \text{ res}_q\alpha _{k}(x) dx\right) } , \; \text{ as } t \rightarrow 1, \end{aligned}$$

since $e^{ 2\pi i\oint _{\gamma _{\theta }-\gamma '_{\theta }} \hbar \alpha _0(x)+ \alpha _{1}(x) dx}=-1$, by explicit computation. Because of the above identities, the inequalities (119) imply that for all $m\ge 0$ there exists a ${\widetilde{C}}_m$ such that

$$\begin{aligned} \left| \exp \big (2 \pi i\sum _{k=2}^{m+1} \hbar ^{k-1} \text{ res}_q\alpha _{k}(x) dx\big )-1 \right| \le {\widetilde{C}}_m |\hbar |^{m+1} , \text{ as } \hbar \rightarrow 0. \end{aligned}$$

It immediately follows that $\text{ res}_{x=q} \alpha _k(x) dx =0, \forall k \ge 2$, for the chosen branch of $\sqrt{Q_0}$. $\square$

### Lifting WKB solutions to $X_s$

By hypothesis the potential $Q_0(x)$ is saddle free, from which it follows that there exists a *k* such that $\Gamma _{k,k\pm 2}$ is not empty, see Remark A.6 (iv). Without losing in generality, we suppose that $\Gamma _{0,\pm 2} \ne \emptyset$ (the other cases are obtained by a rotation). Hence we are in the situation depicted in Fig. 6, and we can fix the roots $x_0,x_1,x_{-1}$ of $Q_0(x)$, as depicted in the same Figure.

We choose 3 branch-cuts of the function $\sqrt{Q_0(x)}$: the $j-th$ cut, $j=-1,0,1$ connects the roots $x_j$ with the point at $\infty$ and and it asymptotic to the ray $\pi + j \frac{2\pi }{5}$, see Fig. 7 below. The elliptic curve $X_s$ is thus realised the Riemann surface of the function $\sqrt{Q_0(x)}$, and we name the lower sheet the one fixed by the requirement $\lim _{x \rightarrow +\infty }{\text {Re}}\sqrt{Q_0(x)}=+\infty$. To represent a curve in $X_s$ as a curve in the two-sheeted covering, we draw a solid line when the curve belong to the upper sheet, and a dashed line otherwise.

After Proposition A.10, the subdominant solutions $y_k, k \in {\mathbb {Z}}/5{\mathbb {Z}}$ are well-approximated on paths $\gamma \in \Gamma ^{\theta }_{k,k'}$ by the m-th WKB approximation (107), namely

$$\begin{aligned} Y_{m}(x;x')= \exp \left\{ \hbar ^{-1} \sum _{k=0}^{m+1} \hbar ^k \int _{x',\gamma }^x \alpha _k(s) ds \right\} . \end{aligned}$$

provided the branch of $\sqrt{Q_0}$ is chosen in such a way that

120 $$\begin{aligned} \lim _{t\rightarrow 0}{\text {Re}}\int _{t}^{t_0} \sqrt{Q_0(\gamma (t))}{\dot{\gamma }}(t) dt =\infty . \end{aligned}$$

Since the formal WKB solutions are written in terms of abelian differential on $X_s$, they are naturally defined on the lift of $\gamma$ to $X_s$, which we call ${\widehat{\gamma }}$. This is not only natural, but also very convenient since there is a unique way of lifting $\gamma$ that enforces condition (120). In fact, taking into consideration our choice of the branch-cuts of $\sqrt{Q_0}$, the lift of any path $\gamma$ belonging to $\Gamma ^{\theta }_{k,k'}$ is defined as follows:

- If $k=0$, ${\widehat{\gamma }}$ lies on the upper sheet for *t* small. In fact, by definition, $\lim _{x \rightarrow +\infty } {\text {Re}}\sqrt{Q_0(x)}=-\infty$, if *x* belongs to the upper sheet.
- If $k\ne 0$, ${\widehat{\gamma }}$ lies on the lower sheet for *t* small.

We finish this Section by analysing the cycles $\gamma _{1,2} \in H_1(X_s^\circ \setminus B,{\mathbb {Z}})^-$ defined in Fig. 7. Their image in $H_1(X_s,{\mathbb {Z}})$ coincide with the cycles $\gamma _{1,2}$ provided by the WKB triangulation, as defined in Section 6 of the Main Text. This indeed is equivalent to the point (i) of the following Lemma.

#### Lemma A.13

The paths $\gamma _1,\gamma _2 \in H_1(X_s^\circ \setminus B,{\mathbb {Z}})^-$ defined in Fig. 7 satisfy the following normalisation

(i) $\int _{\gamma _1} \sqrt{Q_0(x)} dx =2\int _{x_{1}}^{x_0} \sqrt{Q_0(x)} dx$ and $\int _{\gamma _2} \sqrt{Q_0(x)} dx =2\int _{x_{0}}^{x_{-1}} \sqrt{Q_0(x)} dx$ where the right hand side is computed in the upper sheet.
(ii) $${\text {Im}}\int _{\gamma _i}\sqrt{Q_0(x)}dx>0,\, i=1,2$$.
(iii) $$\langle \gamma _1,\gamma _2\rangle =1$$.

#### Proof

(i) and (iii) are self-evident. (ii) We prove ${\text {Im}}\int _{\gamma _1}\sqrt{Q_0(x)}dx>0$, and leave the other case to the reader. Recall the following facts from Section 6.2 of the Main Text:

- $x_1$ and $x_0$ belong to the closure of the simply connecetd domain—we denote by *H*—which is foliated by the horizontal trajectories belonging to $\Gamma _{0,2}$.
- The map $x \mapsto \int _{x_0}^x\sqrt{Q_0(u)}du$ is a conformal map of *H* into a a horizontal strip.
- There is a path *l* connecting $x_1$ with $x_0$ such that the angle between any $\gamma \in \Gamma _{0,2}$ and *l* is a fixed, positive number $\pi \theta , \theta \in ]0,1[$.

If *x* belongs to the upper-sheet then ${\text {Re}}\int _{x_0}^{x}\sqrt{Q_0(u)}du$ increases along $\gamma$ for any $\gamma \in \Gamma _{0,2}$. Since $\int _{x_0}^x\sqrt{Q_0(u)}du$ is conformal, it follows that ${\text {Im}}\int \sqrt{Q_0(x)}dx$ increases along the line *l* connecting $x_1$ with $x_0$. The thesis follows. $\square$

### Proof of the Theorem A.1

The proof of the Theorem is based on the Proposition A.10 and on the computation of the WKB approximation of cross-ratios of asymptotic values, that the author developed in [27, 28].

As it was explained in Sect. A.2 above, the hypothesis that the potential $Q_0(x)$ is saddle free is equivalent to the property that there exists a *k* such that $\Gamma _{k,k\pm 2}$ is not empty. Moreover, we can always reduce to the case that $\Gamma _{0,\pm 2} \ne \emptyset$, hence we are in the situation depicted in Fig. 6 above.

For an arbitrary basis $\lbrace y, {\widetilde{y}} \rbrace$ of solutions to the deformed cubic oscillators, one defines the single-valued meromorphic function $f(x)=\frac{y(x)}{{\widetilde{y}}(x)}$. The function *f* has 5 asymptotic values, $a_k, k \in {\mathbb {Z}}/5{\mathbb {Z}}$, defined by the formula

121 $$\begin{aligned} a_k(\hbar )=\lim _{x\rightarrow + \infty }f(|x|e^{i \frac{2 \pi k}{5}}) \in {\mathbb {P}}^1 , \end{aligned}$$

which is independent on the curves along which the limit is taken.

According to the main text, see Eq. (93), the Fock-Goncharov co-ordinates are defined as cross-ratio of the asymptotic values

122 $$\begin{aligned} X_1(\hbar ):={\text {CR}}(a_0,a_1,a_2,a_{-2}) \quad X_2(\hbar ):={\text {CR}}(a_0,a_2,a_{-2}, a_{-1}) . \end{aligned}$$

Here ${\text {CR}}(a,b,c,d)=\frac{(a-b)(c-d)}{(a-d)(b-c)}$, is the cross-ratio.

In what follows we prove the thesis, namely Eq. (100), for the co-ordinate $X_2$. The proof for the co-ordinate $X_1$ can obtained by repeating the very same steps, and it is therefore omitted.

#### The apparent singularity

Here we prove a generalization of formula (121), which is useful in the presence of one apparent singularity.

We fix a point $x' \in {\mathbb {C}}$ such that $x' \ne q$ and two local linearly independent solutions Moreover we fix two solutions $y,{\widetilde{y}}$. Suppose that we have two paths, $\gamma ,{\widetilde{\gamma }}$, that connects $x'$ to $e^{i\frac{2\pi k}{5}}\infty$, that do not cross $x=q$, and that coincide for large *x*, so that $\gamma -{\widetilde{\gamma }}$ can be thought as a Jordan curve (i.e. a simple closed curve) on ${\mathbb {C}}\setminus \lbrace q \rbrace$. Denoting by $y_{\gamma }(x),{\widetilde{y}}_{{\widetilde{\gamma }}}(x)$ the analytic continuation of $y,{\widetilde{y}}$ along these paths, we obtain the following expression for the asymptotic value $a_k$, which we will need below

123 $$\begin{aligned} a_k=(-1)^{s(\gamma -{\widetilde{\gamma }})}\lim _{x\rightarrow + \infty } \frac{y_{\gamma }(xe^{i \frac{2 k \pi }{5}})}{{\widetilde{y}}_{{\widetilde{\gamma }}}(xe^{i \frac{2 \pi k}{5}})} . \end{aligned}$$

Here *s* is the winding number of $\gamma -{\widetilde{\gamma }}$ around *q*.

The above formula is a consequence of (121) and the following fact: for every non trivial solution, the point $x=q$ is a branch point and the monodromy about *q* is $-1$.

#### The Fock-Goncharov co-ordinates in the small $\hbar$ limit

In order to compute the asymptotic expansion of $X_2(\hbar )$ we need to choose a basis of solutions with a known asymptotic expansion, and then compute the corresponding asymptotic values $a_k$ . Our choice (the only possible) is $\lbrace y_0,y_{-2}\rbrace$ where $y_0$ is the solution subdominant at $+\infty$ and $y_{-2}$ is the solution subdominant at $e^{-\frac{4\pi }{5}i} \infty$.

Notice that $\lbrace y_0,y_{-2}\rbrace$ may in general fail to form a basis of solutions. They do however form a basis, whenever $\hbar$ is small enough. Indeed, let us fix a $\theta \in [0,\frac{\pi }{2}[$. By hypothesis $\Gamma _{0,-2}$ is not empty. Therefore, according to Proposition A.10(3), there exists a $\hbar _{\theta }>0$ such that $\lim _{x \rightarrow + \infty }|y_0(x^{-\frac{4\pi }{5}}i)|=\infty$, for all $\hbar \in S_{\theta ,\hbar _\theta }$. This implies that the solution $y_0$ and the solution $y_{-2}$ are linearly independent. Hence $a_0=0$, $a_{-2}=\infty$, and formula (122) reduces to

124 $$\begin{aligned} X_2(\hbar )=-\frac{a_{2}(\hbar )}{a_{-1}(\hbar )} , \quad \forall \hbar \in S_{\theta ,\hbar _\theta } . \end{aligned}$$

#### Integration paths used in the proof

By hypothesis on the potential $Q_0$, the sets $\Gamma _{0,-2}$,$\Gamma _{0,2}$ are not empty, and the sets $\Gamma _{-2,-1}$,$\Gamma _{-2,2}$, and $\Gamma _{0,-1}$ are not empty for every potential $Q_0$, see Remark A.6.

According to Lemma A.8, for every $\theta \in [0,\frac{\pi }{2}[$, we can choose paths $\gamma _{0,\pm 2} \in \Gamma ^{\theta }_{0,\pm 2},\gamma _{0,-1} \in \Gamma ^{\theta }_{0,1}, \gamma _{-2,0} \in \Gamma ^{\theta }_{-2,0},\gamma _{-2,2}\in \Gamma ^{\theta }_{-2,2}$ satisfying the following properties

1. $\gamma _{0,2}(t)=\gamma _{0,-2}(t)=\gamma _{0,-1}(t)$ for $t \in [0,t_0]$, with $t_0>0$. We denote by $x'=\gamma _{0,2}(t_0)$ the (last) intersection point;
2. $$\gamma _{-2,0}(t)=\gamma _{0,-2}(1-t)$$;
3. $\gamma _{-2,2}(t)=\gamma _{-2,0}(t)=\gamma _{-2,-1}(t)$ for $t \in [0,t_1]$, with $t_1$ small enough. We denote by $x''=\gamma _{-2,0}(t_1)$ the (last) intersection point
4. $$\gamma _{-2,1}(t)=\gamma _{0,-1}(t)$$ as $$t \rightarrow 1$$;

After Proposition A.10, a subdominant solution in the 0-th Sector, $y_0(x)$, is well-approximated by the m-th WKB function

125 $$\begin{aligned} Y^{(0)}_{m}(x;x')= \exp \left\{ \hbar ^{-1} \sum _{k=0}^{m+1} \int _{x',\gamma }^x \hbar ^k \alpha _k(s) ds \right\} , \quad \forall x \in \gamma _{0,2} \cup \gamma _{0,-2} \cup \gamma _{0,-1},\nonumber \\ \end{aligned}$$

Here the integration path $\gamma$ is -depending on *x* - $\gamma _{0,2}$ or $\gamma _{0,-2}$ or $\gamma _{0,-1}$, and the suffix (0) stands to remind that the branch of $\sqrt{Q_0}$ is chosen in such a way that $\lim _{t\rightarrow 0}{\text {Re}}\int _{t}^{t_0} \sqrt{Q_0(\gamma _{0,2}(t))}{\dot{\gamma }}_{0,2}(t) dt =\infty$. More precisely: there is a $\hbar _{\theta }>0$ and a sequence of positive constants $C_{m,\theta }$ such that

126 $$\begin{aligned} \left| \frac{y_0(x)}{Y^{(0)}_{m}(x;x')}-1\right| \le C_{m,\theta } |\hbar |^{m+1}, \quad x \in \gamma _{0,2} \cup \gamma _{0,-2} \cup \gamma _{0,-1}, \hbar \in S_{\theta ,\hbar _{\theta }} . \end{aligned}$$

The same hold for the subdominant solutions in the Sector $-2$. There are are positive constants ${\bar{C}}_{m,\theta }$ and a subdominant solution $y_{-2}(x)$ such that

127 $$\begin{aligned} \left| \frac{y_{-2}(x)}{Y^{(-2)}_{m}(x;x')}-1\right| \le {\bar{C}}_{m,\theta } |\hbar |^{m+1}, \quad x \in \gamma _{-2,0} \cup \gamma _{-2,-1} \cup \gamma _{-2,2}, \hbar \in S_{\theta ,\hbar _{\theta }}. \end{aligned}$$

where

128 $$\begin{aligned} Y^{(-2)}_{m}(x;x')= & {} \exp {\left\{ \hbar ^{-1} \sum _{k=0}^{m+1} -\int _{x'',\gamma _{-2,0}}^{x'} \hbar ^k \alpha _k(s) ds + \int _{x'',\gamma }^x \hbar ^k \alpha _k(s) ds \right\} } ,\, \nonumber \\&\quad \forall x \in \gamma _{0,2} \cup \gamma _{0,-2} \cup \gamma _{0,-1}. \end{aligned}$$

In the above formula the integration path $\gamma$ is -depending on *x* - $\gamma _{-2,0}$ or $\gamma _{-2,-1}$ or $\gamma _{-2,2}$, and the suffix (2) stands to remind that the branch of $\sqrt{Q_0}$ is chosen in such a way that $\lim _{t\rightarrow 0}{\text {Re}}\int _{t}^{t_1} \sqrt{Q_0(\gamma _{-2,0}(t))}{\dot{\gamma }}_{-2,0}(t) dt =\infty$.

As it was explained in the Sect. A.5, the choice of the branch of $\sqrt{Q_0}$ can be enforced by lifting the integration paths to $X_s$, which we described as a two-sheeted covering of the Riemann sphere.^7^ The lift is defined as follows: the lift of $\gamma _{0,2}, \gamma _{0,-2},\gamma _{0,-1}$ belongs to the upper (solid) sheet for $x \rightarrow +\infty$, the lift of $\gamma _{-2,0},\gamma _{-2,-1},\gamma _{-2,2}$ belongs to the lower (dashed) sheet as $x \rightarrow e^{-\frac{4\pi }{5}i} \infty$. Denoting by ${\widehat{\gamma }}_{k,k'}$ the lift of $\gamma _{k,k'}$, for any of the paths introduced, the situation is as illustrated in the *Bacalhau Diagram*, Fig. 8.

#### Computation of the Fock-Goncharov co-ordinates in WKB approximation

We can compute $a_{-1}(\hbar )$ in the WKB approximation using formulas (123,125,126,127, 128). After formula (123), we have

$$\begin{aligned} a_{-1}(\hbar )=(-1)^{s_{-}} \lim _{t\rightarrow 1}\frac{y_0(\gamma _{0,-1}(t))}{y_{-2}(\gamma _{-2,-1}(t))} , \quad s_{-}=s(\gamma _{0,-1}-\gamma _{-2,-1}+\gamma _{-2,0}). \end{aligned}$$

Defining

129 $$\begin{aligned} \epsilon _{-}(\hbar ):=\lim _{t \rightarrow 1}\frac{y_0(\gamma _{0,-1}(t))}{Y^{(0)}_m({\widehat{\gamma }}_{0,-1}(t);x')} \frac{Y^{(-2)}_m({\widehat{\gamma }}_{-2,-1}(t);x')}{y_{-2}(\gamma _{-2,-1}(t))}-1 , \end{aligned}$$

we obtain

$$\begin{aligned} \lim _{t\rightarrow 1}\frac{y_0(\gamma _{0,-1}(t))}{y_{-2}(\gamma _{-2,-1}(t))}= \left( \lim _{t\rightarrow 1}\frac{Y^{(0)}_m({\widehat{\gamma }}_{0,-1}(t);x')}{Y^{(-2)}_m({\widehat{\gamma }}_{-2,-1}(t);x')}\right) \big (1+\epsilon _{-}(\hbar )\big ) \end{aligned}$$

After formulae (125,128), we have that

130 $$\begin{aligned} \lim _{t\rightarrow 1}\frac{Y_0(\gamma _{0,-1}(t);x')}{Y_{-2}(\gamma _{-2,-1}(t);x')}= \exp \left( \hbar ^{-1}\sum _{k=0}^{m+1} \hbar ^{k} \int _{\gamma _2^-}\alpha _k(x) dx\right) , \end{aligned}$$

where $\gamma _2^-$ is the lift -which is not closed- of the closed path $\gamma _{0,-1}-\gamma _{-2,-1}+\gamma _{-2,0}$, as depicted in Fig. 9. Finally, after (126,127), we have that there exists a sequence of positive constants $C_{m,\theta }$ such that

$$\begin{aligned} \left| \epsilon _{-}(\hbar )\right| \le C^-_{m,\theta } |\hbar |^{m+1} , \quad \forall \hbar \in S_{\theta ,\hbar _{\theta }} , \end{aligned}$$

where $\epsilon _{-}(\hbar )$ is the constant defined in (129).

We can use the same strategy to compute $a_2(\hbar )$ to obtain the following statement: There exists a sequence of constants $\hbar _{\theta },C^+_{m,\theta }$ such that

131 $$\begin{aligned}&(-1)^{s_+} a_2(\hbar ) \exp \left( -\hbar ^{-1}\sum _{k=0}^{m+1} \hbar ^{k} \int _{\gamma _2^+}\alpha _k(x) dx\right) =\big (1+\epsilon _{+}(\hbar )\big ), \nonumber \\&\quad \text{ with } \left| \epsilon _{+}\right| \le C_{m,\theta }^+ |\hbar |^{m+1}, \; \forall \hbar \in S_{\theta ,\hbar _{\theta }}. \end{aligned}$$

Here $s_+=s(\gamma _{0,2}-\gamma _{-2,2}+\gamma _{-2,0})$ and $\gamma _2^+$ is the lift of $\gamma _{0,2}-\gamma _{-2,2}+\gamma _{-2,0}$, as depicted in Fig. 9.

We notice that

132 $$\begin{aligned} \gamma _2^+-\gamma _2^-=-\gamma _2 \text{ in } H_1(X_s^{\circ }\setminus B,{\mathbb {Z}})^- \end{aligned}$$

where $\gamma _2^+,\gamma _2^-$ are the curves defined in Fig. 9 and $\gamma _2$ is the basis element of $H_1(X_s^{\circ }\setminus B,{\mathbb {Z}})^-$ defined in Fig. 7. Combining (130), (131), and (132), we obtain the following result: For every $\theta \in [0,\frac{\pi }{2}$, there exist $\hbar _\theta >0$ and a sequence of positive constants $C_{m,\theta }>0,m\ge 0$ such that

133 $$\begin{aligned} X_2(\hbar ) e^{\hbar ^{-1}\int _{\gamma _2}\sqrt{Q_0(x)}dx}= - (-1)^{(s_++s_-)} \big (1+ \epsilon _2(\hbar )\big ) \exp \left( -\hbar ^{-1}\sum _{k=1}^{m+1} \hbar ^{k} \int _{\gamma _2}\alpha _k(x) dx\right) ,\nonumber \\ \end{aligned}$$

where $|\epsilon _{2}(\hbar )|\le C_m |\hbar |^{m+1}$, for all $\hbar \in S_{\theta ,\hbar _{\theta }}$.

We are left to show that Eq. (133) is equivalent to Eq. (100) (for the index $i=2$). Comparing the two equations, we see that they are equivalent if and only if

134 $$\begin{aligned} -(-1)^{(s_++s_-)}\exp \left( -\int _{\gamma _2}\alpha _1(x) dx\right) =\exp \left( - \int _{\gamma _2}{\widetilde{\alpha }}_1(x)+\frac{1}{2(x-q)} dx\right) \nonumber \\ \end{aligned}$$

where ${\widetilde{\alpha }}_1(x)=\alpha _1(x)+\frac{Q'_0(x)}{4Q_0(x)}$ as per (99). This is indeed the case. In fact, by the residue theorem we have that $\int _{\gamma _2}\frac{dx}{2(x-q)}=i \pi \sigma$ where $\sigma$ is the winding number of the projection of $\gamma _2$ around *q*, and $\int _{\gamma _2}\frac{Q'(x)}{4 Q(x)} dx=-i \pi$.

##### Remark A.14

The co-ordinates $X_1,X_2$ are strictly related to the Stokes multipliers of the cubic oscillator. These are defined as follows: For every $k \in {\mathbb {Z}}/5{\mathbb {Z}}$ one chooses a normalisation of the subdominant solutions $y_k,y_{k\pm 1}$ of Eq. (98), see [26] for the precise definition, in such a way that

$$\begin{aligned} y_{k+1}(x)=y_{k-1}(x)+\sigma _k \, y_k(x) \end{aligned}$$

for some uniquely defined $\sigma _k \in {\mathbb {C}}$, which are the Stokes multipliers.

It was proven in [26, §2] that each Stokes multiplier can be expressed as the cross-ratio of 4 asymptotic values, namely

135 $$\begin{aligned} \sigma _k=i {\text {CR}}(a_{k-1}a_{k+1}, a_{k+2},a_{k-2}) . \end{aligned}$$

Now assume that the potential $Q_0$ is saddle-free. It follows that there is a unique $l \in {\mathbb {Z}}/ 5{\mathbb {Z}}$ such that the sets of horizontal trajectories $\Gamma _{l,l\pm 2}$ are not empty. Comparing (135) with (93) we obtain

136 $$\begin{aligned} X_1= \big ( -i \sigma _{l-1}\big )^{-1} \, , \quad X_2= -i \sigma _{l+1} . \end{aligned}$$

## Footnotes

1 In fact this is a slight over-simplification: it is necessary to slightly enlarge the space ${\text {Quad}}(g,m)$ by allowing the zeroes of $\phi$ to collide with any of the poles of order $m_i=2$: see [7, Section 6] for details.

2 Since the preprint version of this paper appeared, these structures have been related to hyperkähler geometry [8].

3 For the general notion of a (possibly non-finite) BPS structure the condition (ii) is replaced with a weaker condition called the support property: see [4, Section 2] for details.

4 For the purposes of comparison with the general situation of [7] involving the hat-homology group $H_1(X_s^\circ ,\mathbb {Z})^-$, note that the group $H_1(X_s,\mathbb {Z})$ coincides with its $-1$ eigenspace under the action of the covering involution of (90); indeed the $+1$ eigenspace can be identified with the first homology of the quotient $\mathbb {P}^1$, which vanishes; moreover, puncturing $X_s$ at the inverse image of the pole $\infty \in \mathbb {P}^1$ also leaves the first homology unchanged.
